# Modeling anorexia nervosa: transcriptional insights from human iPSC-derived neurons

**DOI:** 10.1038/tp.2017.37

**Published:** 2017-03-14

**Authors:** P D Negraes, F R Cugola, R H Herai, C A Trujillo, A S Cristino, T Chailangkarn, A R Muotri, V Duvvuri

**Affiliations:** 1 Department of Pediatrics/Rady Children's Hospital San Diego, Department of Cellular and Molecular Medicine, Stem Cell Program, School of Medicine, University of California San Diego, La Jolla, CA, USA; 2 Graduate Program in Health Sciences, School of Medicine, Pontifícia Universidade Católica do Paraná, Curitiba, Brazil; 3 The University of Queensland Diamantina Institute, Translational Research Institute, Brisbane, QLD, Australia; 4 National Center for Genetic Engineering and Biotechnology, Virology and Cell Technology Laboratory, Pathum Thani, Thailand; 5 Department of Pediatrics and Psychiatry, School of Medicine, University of California San Diego, La Jolla, CA, USA

## Abstract

Anorexia nervosa (AN) is a complex and multifactorial disorder occurring predominantly in women. Despite having the highest mortality among psychiatric conditions, it still lacks robust and effective treatment. Disorders such as AN are most likely syndromes with multiple genetic contributions, however, genome-wide studies have been underpowered to reveal associations with this uncommon illness. Here, we generated induced pluripotent stem cells (iPSCs) from adolescent females with AN and unaffected controls. These iPSCs were differentiated into neural cultures and subjected to extensive transcriptome analysis. Within a small cohort of patients who presented for treatment, we identified a novel gene that appears to contribute to AN pathophysiology, *TACR1* (tachykinin 1 receptor). The participation of tachykinins in a variety of biological processes and their interactions with other neurotransmitters suggest novel mechanisms for how a disrupted tachykinin system might contribute to AN symptoms. Although *TACR1* has been associated with psychiatric conditions, especially anxiety disorders, we believe this report is its first association with AN. Moreover, our human iPSC approach is a proof-of-concept that AN can be modeled *in vitro* with a full human genetic complement, and represents a new tool for understanding the elusive molecular and cellular mechanisms underlying the disease.

## Introduction

Anorexia nervosa (AN) is a multifactorial neurodevelopmental disorder of unknown etiology affecting around 1% of the population.^1^ With a stereotypic post-pubertal onset, this complex neuropsychiatric condition is primarily seen in female adolescents and young women between 15–19 years old.^2, 3, 4^ Anorexia presents with distorted body image and food restriction to the point of severe emaciation or death, likely resulting from a highly anxiogenic response to intake of palatable foods.^5^ Although exhibiting the highest mortality rates among psychiatric illnesses, with patients facing consequences such as high chronicity and morbidity, there are no treatments to reverse AN symptoms.^6, 7, 8^ Understanding the pathophysiology of AN has lagged behind other psychiatric disorders, consequently, eating disorders in general and AN in particular, have been viewed as non-biologically based problems of vanity, poor parenting or pertaining to specific groups of individuals.^9^ Several studies suggest that genes contribute significantly to neurobiological vulnerabilities, accounting for approximately 50–75% of the risk for AN.^4, 10, 11^ However, the identity of specific genes underlying the disease remains largely unexplored and the mechanisms are poorly understood. AN appears to aggregate in families and relatives of affected individuals seem to have an elevated risk of obsessive-compulsive personality disorder.^12^ It is also suggested that first degree relatives carry a relative risk of 11.3 compared to the general population,^12, 13^ with mothers showing increased rates of affective, substance use and anxiety disorders.^14^ In addition, various genetic studies have implicated a network of risk-conferring genes involved in serotonin and dopamine neurotransmission.^15, 16, 17, 18, 19, 20^ Functional neuroimaging studies corroborated these hypotheses^19, 21, 22, 23, 24^ by revealing a dysfunctional dopaminergic (DA) reward circuit in AN.

Despite progress in assigning circuits to neuropsychiatric disorders, disease-specific molecular and cellular phenotypic data for regions of interest is either limited, skewed by chronic illness in post-mortem samples or absent. This problem can now be addressed by reprogramming somatic cells into a pluripotent state by ectopic expression of specific genes.^25^ Such induced pluripotent stem cells (iPSCs) can be differentiated into neurons and have been generated for several complex neurological conditions.^26, 27, 28, 29, 30, 31^ Using this approach, we are able to test hypotheses and to evaluate whether the captured genome from human individuals afflicted with AN alters cellular phenotypes as predicted by mechanistic models of the disease. An AN-iPSC model may address human-specific effects and avoid some aspects of the well-known limitations of animal models such as the absence of a human genetic background.^32^

Here, we examined the expression profile of human AN-derived neurons after generating iPSCs from AN patients. We accessed not only targeted gene expression levels but we also performed whole transcriptome-based bioinformatics to reveal AN-risk conferring transcripts/genes that could be associated with disease initiation and progression. Our study represents a proof-of-concept that AN can be modeled *in vitro*. Although no predicted differences at neurotransmitters levels were observed, we identified a disruption of the tachykinin system that might contribute to AN pathophysiology before other phenotypes become prominent. While we believe that further validation using a larger cohort of patients is important, our work brings a novel technological advancement to the field of eating disorders. These findings could transform our ability to study how AN risk-conferring genetic variations perturb molecular pathways and cellular networks, highlighting potential approaches for new therapies.

## Materials and methods

### Participant ascertainment

We isolated and derived fibroblasts from seven females with AN, and four healthy female controls. The biopsied individuals, control CTL1 and patient AN1, are sisters. Fibroblast donations were solicited from patients who were receiving treatment at the outpatient clinic of the UCSD Eating Disorders Treatment and Research Center. For inclusion, patients had to be female and meet Diagnostic and Statistical Manual of Mental Disorders, 4th edition (DSM-IV)^33^ criteria for AN, except for the amenorrhea criterion. AN individuals exhibited a stereotypical, post-pubertal onset of weight loss from self-reducing food intake, fear of weight gain while underweight, with or without compensatory behaviors such as over-exercise and purging. In order to reduce a major source of heterogeneity, AN patients were selected for severity of symptoms and presence of serious medical and behavioral consequences of AN-related behaviors. This was feasible because all AN participants were recruited at a tertiary center for specialized, intensive eating disorders treatment, while receiving treatment under one of the authors. These recruitment characteristics ensured that the AN cohort not only met DSM-IV criteria but also met more stringent and homogeneous medical necessity criteria for severity of illness. AN individuals in our cohort had a relentless drive to lose weight that had actually resulted in weight loss. Treatment with their outpatient providers and efforts at home to restore weight was met with severe emotional dysregulation and/or medical abnormalities from worsening eating disordered behaviors. Furthermore, we were able to clearly assess these symptoms as the parents of the patients were seeking treatment for their adolescent daughters proximal to onset of their illness. AN individuals who over-exercised as the only compensatory method as well as others who used purging via self-induced vomiting, laxatives, among others, were included. Donations from healthy controls were solicited from siblings of AN patients along with unaffected individuals participating in other ongoing research studies. For inclusion, controls had to be female and should have never met an Axis I diagnosis per DSM-IV. Briefly, fibroblasts were obtained from skin punch biopsy performed by a Staff Dermatologist at the UCSD Outpatient Dermatology Clinic. Written informed consent was acquired from volunteers and their parents (when applicable), and all human research protocols were approved by the UCSD Institutional Review Board.

### Generation of iPSCs

Skin fibroblasts from 8 individuals, that is, 4 AN patients and 4 controls, were reprogrammed into iPSCs. Cells were transduced with four retroviral constructs (*OCT4*, *SOX2*, *KLF4* and *c-MYC*), as previously described.^25, 30^ Forming iPSCs were transferred to inactivated mouse embryonic fibroblast (MEF) feeders in human embryonic stem cell (hESC) medium (DMEM/F12 (Corning, Life Technologies, Carlsbad, CA, USA), 20% Knockout Serum Replacement (KSR; Life Technologies), 20 ng ml^−1^ basic fibroblast growth factor (bFGF; Life Technologies), 2 mm Glutamax (Gibco, Life Technologies), 1% non-essential aminoacids (NEAA; Sigma-Aldrich, St Louis, MO, USA), 0.1 mm β-mercaptoethanol (Sigma-Aldrich) and 1 mm of valproic acid (Sigma-Aldrich). hESC-like colonies, characterized by their compact morphology with a high nucleus-to-cytoplasm ratio, were manually dissected and passaged to pre-coated Matrigel (BD Biosciences, San Jose, CA, USA) plates in mTeSR1 medium (Stem Cell Technologies, Vancouver, BC, Canada). A minimum of 2 iPSC clones from each individual were selected for expansion.

### Karyotyping

Standard G-banding karyotype from fibroblasts and iPSC clones was performed by the Stem Cell Core Facility at USC (Los Angeles, CA, USA), in collaboration with the Children's Hospital Los Angeles.

### Teratoma formation

Feeder-free fully grown iPSCs were dissociated with collagenase for 3–4 min at 37 °C and re-suspended in PBS and Matrigel (1:1). Next, 1–3 × 10^6^ cells were injected subcutaneously in the lower hind leg and near the ankle of immune compromised mice. After 8 weeks, the tumor was dissected, fixed in 4% paraformaldehyde and paraffin embedded. For histological studies, sections of 5 μm thickness were stained with hematoxylin and eosin, and analyzed for the presence of the three different germ layer tissues. Protocols were approved by the UCSD Institutional Animal Care and Use Committee.

### Embryoid body formation

Assessment of the tri-lineage differentiation capacity of iPSCs was performed *in vitro* through embryoid body (EB) formation. Briefly, iPSCs were mechanically dissociated and cultured in suspension for 14 days in non-adherent dishes to form EBs in DMEM-F12, 20% FBS. The presence of the three germ layers was investigated by gene expression (Supplementary Table S1).

### Differentiation of iPSCs into cortical neurons

IPSC medium was changed to N2 medium comprising DMEM/F12 with l-Glutamine and 15 mm HEPES, 1 × N2 NeuroPlex (Gemini Bio-Products, West Sacramento, CA, USA), 1 μm dorsomorphin (R&D System, Minneapolis, MN, USA) and 10 μm SB431542 (Stemgent, Lexington, MA, USA) for 2 days. Next, cells were grown in suspension for 7 days in N2 medium. The formed EBs were gently dissociated and plated onto Matrigel-coated dishes using neural induction (NI) medium (DMEM/F12 with l-Glutamine and 15 mm HEPES, 0.5 × N2 NeuroPlex, 1 × Gem21 NeuroPlex (Gemini Bio-Products) and 20 ng ml^−1^ bFGF)). Rosettes that emerged were manually picked, dissociated with Accutase (Life Technologies) and re-plated onto 10 μg ml^−1^ Poly-l-ornithine and 5 μg ml^−1^ laminin-coated (from Sigma-Aldrich and Life Technologies, respectively) plates. Homogeneous populations of neural progenitor cells (NPC) were expanded using NI medium. The differentiation into neurons was performed upon bFGF withdrawal and addition of 5 μM ROCK inhibitor (Y-27632, Calbiochem, La Jolla, CA, USA). Cells were cultivated for 4 weeks with media changes every 3–4 days.

### Immunocytochemistry

Cells were fixed with paraformaldehyde, permeabilized and blocked in 3% bovine serum albumin and 0.1% Triton X-100. Primary antibodies were incubated overnight at 4 °C; secondary antibodies were incubated for 1 h at room temperature. Nuclei were stained with DAPI solution (1 μg ml^−1^). Slides were mounted using Prolong Gold antifade reagent (Life Technologies). Images were captured using a Zeiss microscope (Carl Zeiss, Jena, Germany). For antibodies specifications, refer to Supplementary Table S2.

### Gene expression analysis

Total RNA was reverse transcribed with QuantiTect Reverse Transcription Kit (Qiagen, Valencia, CA, USA). To investigate the expression of pluripotency and the three germ layer genes, cDNA from iPSCs and EBs was amplified by PCR. qRT-PCR was also performed for selected genes using specific primers and iQ SYBR Green supermix (Bio-Rad Laboratories, Irvine, CA, USA). The expression of each target gene and the reference gene (*B2M*, beta-2-microglobulin and/or *GAPDH,* Glyceraldehyde-3-phosphate dehydrogenase) was measured in technical triplicates for each reaction. The relative expression quantification was normalized using the 2^(−ΔΔCt)^ method. The AN and control groups were compared by Student's *t*-test. For primer sequences, refer to Supplementary Table S1. The Human Neurotransmitter Receptors RT^2^ Profiler PCR Array (#PAHS-060Z; SABiosciences, Qiagen, Valencia, CA, USA) was used for the quantitative gene expression analysis of neuronal cultures, as suggested by the manufacturer.

### Western blotting

Twenty micrograms of total protein extracts were separated in Bolt 4–12% Bis-Tris Plus Gel (Life Technologies) and transferred onto a nitrocellulose membrane using Thermo Fisher's iBlot2 dry blotting system. After blocking (Rockland Immunochemicals, VWR International, Arlington Heights, IL, USA), membranes were incubated with primary antibodies overnight at 4 °C and then, with secondary antibodies for 1 h at room temperature. Proteins were detected using Odyssey CLx infrared imaging system (LI-COR Biosciences, Lincoln, NE, USA).

### Transcriptome analysis

RNA-sequencing (RNA-seq) analysis was performed in 10 μg of total RNA using Illumina Hiseq-2000 (Illumina, San Diego, CA, USA). Raw sequenced RNA-seq libraries were filtered for high-quality reads based on read average quality, and per position nucleotide detection using NGS QC Toolkit software.^34^ High-quality reads were mapped to the human reference genome (Hg19) using Star aligner,^35^ producing compressed binary BAM files. Next, binary BAM files were subjected to HTSeq software package^36^ to account for absolute number of mapped reads per annotated transcript (Ensembl GRCh37 annotation) in Hg19, generating a count data matrix. This matrix was then normalized by a read counting approach followed by a negative binomial distribution and Fischer's exact statistical test for differential expression (DE) analysis using the R Bioconductor package DESeq.^37^ For statistical significance calculation between samples, false discovery rate based on Benjamini & Hochberg method^38^ was applied over the DE genes, with DE transcripts having *P*-value<0.05. Count data was used to calculate Euclidian distance between each pair of samples, including biological replicates, which were clustered based on shorter distances. In addition, count data matrix was also used to create a heatmap together with a hierarchical clustering-based dendrogram, and a two-component-based Principal Component Analysis (PCA), to show relative relationship between sequenced AN and control samples in a 2D coordinate space. For molecular network and functional pathway annotation robustness, we considered in the analysis only those genes common to both annotation databanks, ENSEMBL (GRCh38.82) and HUGO Gene Nomenclature Committee (HGNC). There are 35 644 genes registered in the HGNC database and 60 619 genes in the ENSEMBL gene annotation database, with 35 588 genes commonly identified in both databanks.

### Gene regulatory networks and functional pathways analyses

The protein–protein interaction network (PIN) was constructed by using the protein-coding differentially expressed genes (DEGs) and their direct neighbors (or first-degree interactors). The PIN data were retrieved from the Biogrid database (v 3.4.127).^39^ The statistical analysis of network properties and structure was carried out using Python programming language and igraph package (igraph.sourceforge.net). Network visualization and annotation were performed using Cytoscape^40^ (http://cytoscape.org/). ClueGO^41^ was used to find functional pathways enriched in AN-PIN based on functional annotation databases such as Gene Ontology^42^ (GO; http://geneontology.org/) and Reactome^43^ (http://www.reactome.org). For statistical analysis of PINs, we used a computational method published elsewhere^44^ to measure two structural properties: average shortest path length and density of the network. We tested whether the distribution of those structural properties was similar between AN-PIN and random PIN. The non-parametric Kolmogorov-Smirnov test as implemented in R (www.r-project.org) was used to test whether the structural properties of AN-PIN were similar to random PINs (null hypothesis). The transcriptional profiles of specific genes in the developing human brain were investigated using available RNA sequencing data from BrainSpan database (http://www.brainspan.org).^45^ These data were generated across 13 developmental stages in 8–16 brain structures.

## Results

### Reprogramming somatic cells from AN patients and controls into iPSCs

**Figure 1:**
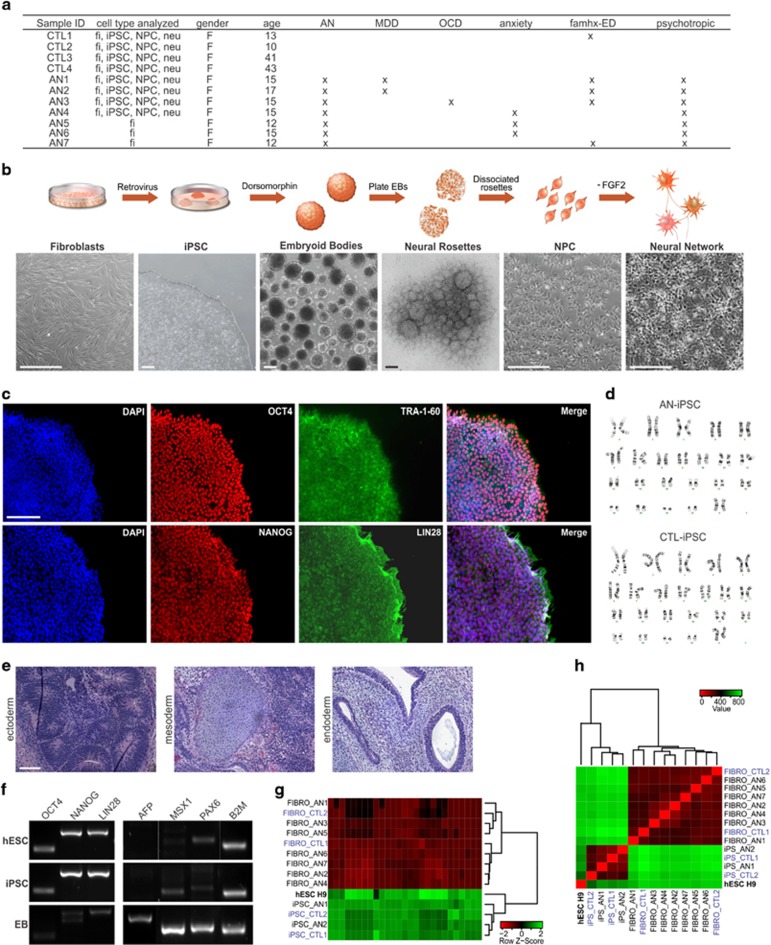
Generation and characterization of iPSCs from AN patients. (**a**) Patient profile summary. (**b**) Schematic view and representative images showing the morphological changes observed during the primary fibroblast cell reprogramming, neural induction and differentiation processes. Scale bars represent 200 μm. (**c**) Representative immunofluorescence images illustrating the expression of pluripotency markers in the generated iPSCs, including OCT4, NANOG, TRA-1–60 and LIN28. Scale bar represents 100 μm. (**d**) Representative images of G-banding karyotype analysis from cell chromosomes demonstrating the genetic stability of iPSCs; no karyotypic abnormalities were induced by the reprogramming process. (**e**) Representative images of hematoxylin and eosin staining of teratomas derived from iPSCs showing tissues from the three germ layers. Scale bar represents 100 μm. (**f**) Expression of pluripotency and three germ layer markers in iPSCs and EBs, respectively, assessed by RT-PCR (*OCT4*, *NANOG* and *LIN28*—pluripotency; *AFP*—endoderm; *MSX1*—mesoderm; *PAX6*—ectorderm). The H9-hESC was used as a control for pluripotency and differentiation capability; *B2M* was used as reference gene. (**g**) Cluster analysis showing correlation coefficients of RNA-seq transcripts from iPSCs and hESC, and a distinguished gene expression profile from primary fibroblast cells (FIBRO). A panel of human pluripotency-related genes (isoform level; Supplementary Table S3) was considered. (**h**) Heatmap and hierarchical clustering-based dendrogram of hESC, iPSCs and fibroblasts for AN and control samples. Considering the entire cellular transcriptome expression profile of evaluated cells, two subgroups were identified: iPSCs, with a molecular signature similar to that exhibited by hESCs, and fibroblasts with a completely different expression profile. In **g** and **h**, colors indicate the range of each gene's expression, with least expression shown in red and highest expression shown in green. AN, anorexia nervosa; anxiety, patient showed/treated for anxiety; CTL, control (unaffected individual); famhx-ED, first, second or third degree relative with a history of an eating disorder; fi, fibroblast; iPSC, induced pluripotent stem cell; MDD, major depressive disorder; neu, neurons; NPC, neural progenitor cell; OCD, obsessive compulsive disorder; psychotropic, patient was prescribed at least one psychotropic medication.

All AN patients included in this study had met the DSM-IV criteria for AN; control individuals (CTL) had never met any Axis I diagnosis. AN1 and CTL1 are siblings. The phenotypic characterization of AN subjects is presented in Figure 1a. A schematic view of our protocol for iPSC reprogramming and differentiation is shown in Figure 1b, and the cell types used in each experiment is detailed in Supplementary Table S3. Two iPSC clones from 4 AN patients and 4 controls, in a total of 16 cell lines, were mechanically expanded for at least 10 passages and tested for the expression of the pluripotency markers *OCT4* (also known as *POU5F1*), *TRA-1-60*, *NANOG* and *LIN28* (Figure 1c; Supplementary Figure 1a, b). Apart from sample AN2, which exhibited an extra structurally abnormal chromosome (ESAC) that was also present in the original fibroblasts, all iPSC clones derived had maintained a normal karyotype when analyzed by G-banding (Figure 1d; Supplementary Figure S1c).

The ability of clones to differentiate into three germ layers was evaluated *in vivo* and *in vitro*. Teratomas showed derivatives from all three embryonic germ layers (ecto-, meso- and endoderm), confirming that the iPSCs were pluripotent and able to differentiate into complex tissues *in vivo* (Figure 1e). The EBs generated in suspension were tested by RT-PCR and we observed that the expression of endogenous pluripotency markers characteristic for hESC were exhibited in all reprogrammed iPSCs (*OCT4*, *NANOG* and *LIN28*), while genes related with the three germ layers were only identified in the EBs (*AFP*, α-fetoprotein—endoderm; *MSX1*, Msh homeobox 1—mesoderm; *PAX6*, paired box homeotic gene 6—ectoderm; Figure 1f). In addition, considering a panel of 8 different genes (and their isoforms) associated with pluripotency, we compared the iPSCs and their fibroblasts counterparts used for the reprogramming process with a hESC line (H9; Supplementary Table S4). The high throughput RNA-seq and bioinformatics data analysis of these cells showed that the iPSC clones were indistinguishable from hESC lines and were also very similar to each other, but distinct from primary fibroblast cells (Figure 1g). Next, by investigating the global transcriptome expression signatures of these cell types we identified two subgroups, iPSC/hESC and fibroblasts, which have completely distinct expression profiles and yet a high degree of similarity within each subgroup when examined for pluripotency-related gene markers (Figure 1h). Together, these data show that the generated iPSC lines successfully re-established pluripotency at the molecular and cellular levels.

### Differentiation of AN and control iPSCs into neurons

**Figure 2:**
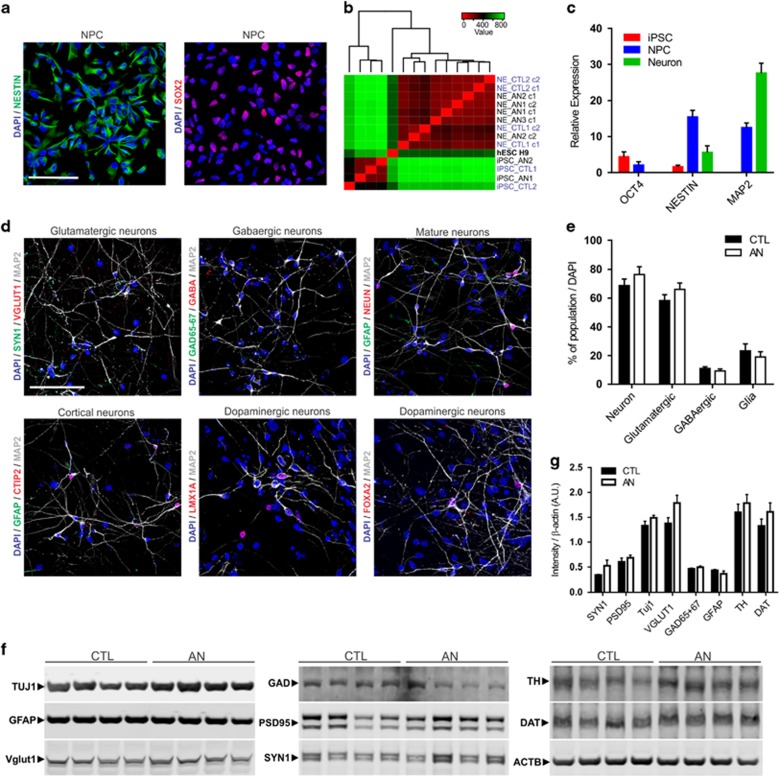
Derivation of neural progenitor cells and neurons from AN-iPSCs. (**a**) Representative images showing that the neural progenitor stage-specific markers NESTIN and SOX2 are expressed by AN iPSC-derived NPCs. Scale bar represents 100 μm. (**b**) Cluster analysis showing that after 4 weeks of differentiation neurons (NE) exhibit a molecular signature distinct from that of their iPSC counterparts. Colors indicate the range of each gene's expression, with least expression shown in red and highest expression shown in green. (**c**) Gene expression changes observed during the differentiation process measured by qRT-PCR using stage-specific markers for iPSCs (*OCT4*), NPCs (*NESTIN*) and neurons (*MAP2*). The expression levels of each gene were quantified, normalized to *B2M* (reference gene), and the results are presented as mean±s.e.m. (*n*⩾8 for each group). (**d**) Representative immunofluorescence images of cells after neuronal differentiation. IPSC-derived neural cultures express neuronal (MAP2, NEUN) and glial (GFAP) markers, together with specific cortical proteins (CTIP2). Excitatory (VGLUT1 and SYN1) and inhibitory (GAD65-67 and GABA) neuronal proteins are also observed in the generated neural population. The presence of LMX1A and FOXA2 among the neuronal cells, although low, is an evidence of the dopaminergic neuronal fate. Scale bar represents 100 μm. (**e**) Quantification of the percentage of MAP2^+^ (neuron), VGLUT1^+^ (glutamatergic), GABA^+^ (GABAergic) and GFAP^+^ (glia) labeled cells is presented as mean±s.e.m. (*n*⩾8 for each group). (**f**) Representative western blotting of control and AN-derived neural proteins that were lysed and immunoblot for neuronal (TUJ1, VGLUT1, GAD65-67, PSD95, SYN1 and TH) and glial (GFAP) markers, along with the dopamine transporter (DAT); β-ACTIN was used as housekeeping control (reference). (**g**) Quantification of proteins in AN and control neural cultures assessed by Western blot analysis; β-ACTIN was used for normalization (*n*=8 for each group). No differences were observed between control and affected samples (*P*<0.05, Student's *t*-test). AN, anorexia nervosa; iPSC, induced pluripotent stem cell; NPC, neural progenitor cells.

For all 16 iPSC clones used in this study, dissociated rosettes formed a homogenous population of NPCs after a few passages, that is positive for early precursor markers such as NESTIN, MUSASHI1 and SOX2 (Figure 2a, Supplementary Figure S1d–f). Next, we compared the global molecular signatures from iPSC and neuronal cultures after 4 weeks of differentiation. Two different gene expression profiles were observed, confirming that we were able to efficiently differentiate the generated pluripotent cells into neurons (Figure 2b). The comparison of iPSC, NPC and neurons according to their levels of expression of *OCT4*, *NESTIN* and *MAP2* clearly showed that *OCT4* was highly expressed in iPSCs but not in the other cell types, while in progenitor cells and neurons *NESTIN* and MAP2 were preferentially upregulated, respectively (Figure 2c). Using the described protocol, NPC differentiation gave rise to a mature population of cortical human neurons expressing MAP2, GFAP, NEUN and CTIP2 (also known as BCL11B; cortical layer V; Figures 2d and e; Supplementary Figure S1g). Moreover, in these differentiated cultures we also detected the presence of both excitatory glutamatergic pyramidal-shaped neurons (VGLUT1 and SYN1) and inhibitory GABAergic interneurons (GAD65-67 and GABA) along with LMX1A and FOXA2 positive cells, which are selectively expressed in progenitors committed to the generation of midbrain dopaminergic neurons (Figure 2d and e; Supplementary Figure S1g-i). Western blot analysis corroborated the immunostaining results and confirmed that the overall composition of neuronal cultures does not change between controls and AN patients (Figures 2f and g; Supplementary Figure S2a, c).

### Gene expression profile of iPSC-derived neurons

**Figure 3:**
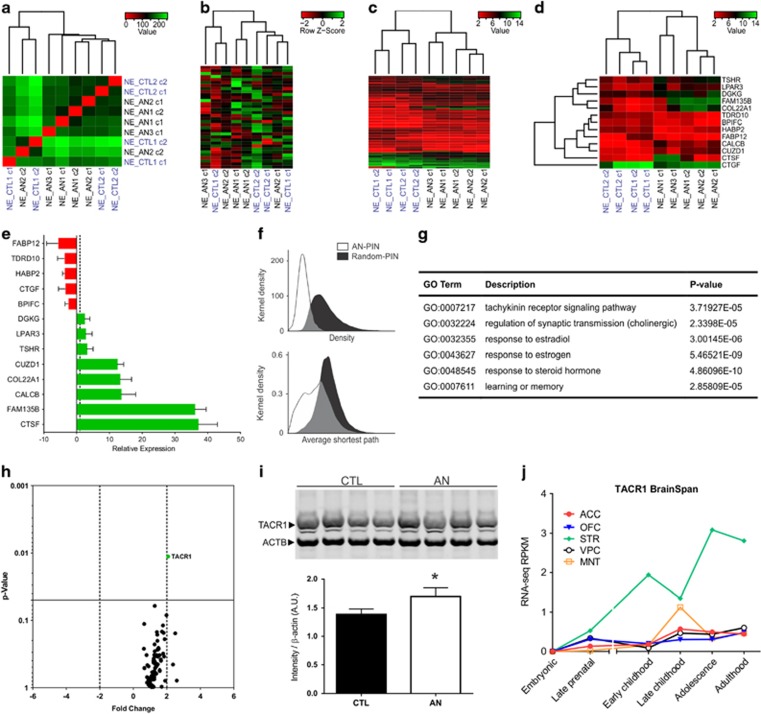
Transcriptional analysis of AN iPSC-derived neuronal cultures. (**a**) Heatmap and the hierarchical clustering-based dendrogram of samples after 4 weeks of differentiation. No significant differences are observed between iPSC-derived neurons (NE) from AN patients and controls. (**b**) Heatmap and the hierarchical clustering-based dendrogram displaying the transcriptional pattern of genes associated with neural development and differentiation in AN and control neural cultures (list of genes at Supplementary Table S5). (**c**) Heatmap and hierarchical clustering-based dendrogram of genes with minimum fold-change variation of 2 and false discovery rate (FDR)-adjusted *P*-value<0.01 between AN and control neurons. (**d**) Neuronal specific cell type-based clustering analysis of AN and control samples using 13 selected candidate genes most differentially expressed between the two subgroups (*P*<0.01). In all heatmaps (**a**–**d**), colors indicate the range of each gene's expression, with least expression shown in red and highest expression shown in green. (**e**) Validation of 13 selected candidate genes from the RNA-seq analysis by qRT-PCR. Downregulated (red histogram) or upregulated (green histogram) genes differentially expressed between AN and control neurons. Independent neuronal cultures generated from the same clones used in the RNA-seq analysis were used in this validation; *GAPDH* was used as reference gene. Error bars are represented by standard deviation. (**f**) Topological structure of AN-PIN and random-PIN showing that AN-PIN is denser and with smaller shortest path than random-PIN (Kolmogorov–Smirnov test, *P*<0.01). (**g**) Enriched GO functional pathways terms found in neurons derived from AN patients (the complete list is found in the Supplementary Table S8). (**h**) Volcano plot of PCR array analysis for human neurotransmitter receptors. Plot illustrates that although control and AN-derived neurons do not show significant differences in expression for estrogen receptors and dopamine/serotonin neurotransmitter systems constituents, the *TACR1* gene is upregulated in AN neurons (2.0-fold differential expression between the groups at *P*<0.05; Student's *t*-test). (**i**) Upper panel: representative western blotting of control and AN-derived neural proteins that were lysed and immunoblot for TACR1; β-ACTIN was used as reference. Bottom panel: quantification of TACR1 protein in AN and control neural cultures assessed by Western blot analysis. Increased levels of TACR1 were observed in AN (*P*<0.05, Student's *t*-test). Proteins were detected using Odyssey CLx infrared imaging system. (**j**) BrainSpan analysis of the *TACR1* gene in brain regions from striatal networks. RNA-seq RPKM (reads per kilobase per million) identified during the different stages of human brain development. ACC, anterior cingulate cortex; AN, anorexia nervosa; iPSC, induced pluripotent stem cell; MNT, mediodorsal nucleus of thalamus; OFC, orbital frontal cortex; STR, striatum; VPC, ventrolateral prefrontal cortex.

After 4 weeks of differentiation, the gene expression profile of AN neurons and controls was investigated. A total of 24 944 genes were found to be transcribed in the iPSC-derived neurons from AN patients and healthy individuals; 361 DEGs (156 upregulated and 205 down-regulated) were identified in AN compared to controls. The DEGs are annotated as four locus groups: 248 protein-coding genes, 50 non-coding RNAs, 57 pseudogenes and 6 others (Supplementary Table S5). The global molecular signature obtained after RNA-seq showed that both neuronal populations are very similar (Figure 3a). When we narrowed down this analysis considering only a panel of genes related to neural development and differentiation, no significant differences were observed (Figure 3b; Supplementary Table S6) suggesting that noticeable developmental anomalies might not be present in AN patients' brain. After considering genes differentially expressed between affected and unaffected subjects with a statistical significance of 95% (fold change⩾2, *P*<0.05, 545 genes; Supplementary Table S7), we increased the stringency of our analysis by including only targets with 99% significance (fold-change⩾2, *P*<0.01, 110 genes; Supplementary Table S8) and found that AN samples cluster together in a subgroup different from that of control individuals (Figure 3c). Interestingly, when 13 candidate genes were selected based on their altered expression in AN with fold-change variation of 2 or more, the clustering algorithm produced subgroups with affected and control samples that remained distinct (Figure 3d). We validated the RNA-seq results for these 13 genes by qRT-PCR and confirmed the same expression patterns observed in the transcriptome analysis (Figure 3e), suggesting that such genes might contribute to the anorexic phenotype.

To identify functional pathways and molecular interactions associated with DEGs in AN neurons, we first built a protein-protein interaction network (PIN) using the 248 protein-coding DEGs (*P*<0.01; Supplementary Table S5) and their direct neighbors (first-degree interactors). Approximately 68% of DEGs (168) were found in the protein-protein interaction database and the AN-PIN is comprised of 1492 proteins and 2254 interactions. The topological structure of AN-PIN is different from PIN built out of randomly selected genes indicating that DEGs in AN have higher connectivity than expected by chance (Kolmogorov-Smirnov test *D*=0.81, *P*<0.01), and smaller average shortest path (Kolmogorov–Smirnov test *D*=0.41, *P*<0.01; Figure 3f). These data suggest that proteins encoded by co-expressed genes in AN subjects are significantly more inter-connected and are likely to be associated with closely related molecular pathways including TACR1 (tachykinin receptor 1) signaling, regulation of synaptic transmission through the cholinergic system and response to estrogen, among others (Fisher's exact test with Bonferroni correction *P*<0.01; Figure 3g; Supplementary Table S9).

Several neurotransmitters and peptides, or their metabolites, can potentially contribute to the AN phenotype, including weight loss, feeding regulation and the reward system.^15, 22, 46, 47^ Then, based on the enriched pathways revealed by the DEGs of our sample cohort, we decided to investigate the neurotransmitter profile of AN-derived neuronal cultures using a PCR array platform. This approach was complemented by the addition of specific targets pertaining to the dopaminergic pathway, estrogen receptors and tachykinin signaling (Supplementary Table S1). Notably, both control and AN neurons are very similar except by the expression of the *TACR1* gene, also known as *NK1R* (neurokinin 1 receptor) or *SPR* (substance P receptor), which was significantly upregulated in AN (Figure 3h). In confirmatory studies, the *TACR1* gene was found upregulated after RNA-seq analysis with a significance of 95% (Supplementary Table S7), while the *TAC1* (tachykinin precursor 1) gene had a reduced expression of almost two fold-change (Supplementary Table S7 and S8) in AN neuronal samples. The protein levels of TACR1 were also increased in AN neurons compared to controls (Student's *t*-test, *P*<0.05; Figure 3i; Supplementary Figure S2a, b). Furthermore, the BrainSpan analysis of the *TACR1* gene within brain regions associated with striatal networks showed its expression peaks during adolescence, the stage of human development in which AN has its onset (Figure 3j). Although *TACR1* has previously been associated with psychiatric disorders, we believe this is the first report of its contribution to AN.

## Discussion

AN is a severe psychiatric disorder that still lacks effective treatments.^7, 8^ Several studies have been conducted aiming to identify the genetic basis of AN. Genome-wide association approaches (GWAS) have emerged as a promising tool for genetic screening. Even though single-nucleotide polymorphisms and/or copy-number variations have been identified, these findings require further analysis in independent cohorts and no study was powerful enough to robustly link specific genes with AN.^10, 48, 49, 50, 51, 52^ The difficulty in validating potential candidates likely arises from the need for large sample populations, which is particularly difficult for uncommon disorders like AN, that has an incidence far <1% of adolescents. Therefore, the genetic underlying AN susceptibility remains largely unknown. The literature in AN is mostly derived from DNA-based assays performed in blood samples or animal models. In this context, the ability to perform a transcriptome analysis to uncover genes implicated in AN pathophysiology using human neural cells could provide a major advantage toward understanding the etiology of the disorder.

Following the reprogramming of skin fibroblasts into iPSCs, we generated neural cultures that allowed us to investigate the gene expression profile of neurons derived from AN patients. The global molecular signature of neurons after RNA-seq analysis showed that AN and control cultures are, overall, very similar to each other. However, two subgroups were identified via a high-stringency analysis using differentially expressed genes with a statistical significance of 99% between affected and unaffected individuals. Among the misregulated genes found, *CTGF* (connective tissue growth factor), which is critical for normal ovarian follicle development and ovulation,^53^ was downregulated in AN samples. This result is congruent with the amenorrhea symptom typically observed in patients.^54^ The misregulation of CTGF in our cohort, despite not using amenorrhea as a diagnostic criterion, might serve as preliminary support for omission of this criterion. The lower expression of *TDRD10* (tudor domain containing 10) could also contribute to AN pathophysiology, since the TDRD family of proteins is known to be relevant for gametogenesis^55^ and anorexics became at least temporarily infertile.^56^ Interestingly, the *FABP12* (Fatty-acid Binding Protein 12) gene, which is related to lipid metabolism as other FABP members,^57^ was downregulated in AN-neural cultures, accompanied by high expression levels of *TSHR*. Since *TSHR* is detected in preadipocytes and adipocytes,^58, 59^ and was shown to participate in adipogenesis,^60^ disruption in this gene was already associated with body weight and energy consumption problems.^61^ Chen *et al.*^62^ demonstrated that increased *TSHR* inhibits *FASN* (fatty acid synthase) expression or energy storage in mature adipocytes, showing that impairments in TSH/TSHR signaling could participate in the development of obesity. In the same way, the *DGKG* (Diacylglycerol Kinase Gamma) gene, that had been associated with chronic stress^63^ and obesity,^64^ was found upregulated in AN-neurons. These findings suggest that misregulation of several genes, primarily unrelated, could contribute to the genesis and/or symptomatology of this multifactorial disease in a synergistic manner.

Despite the limitation of GWAS in finding statistical significance for risk-conferring genes in AN, a variety of studies have identified the serotonin and dopamine systems as major players in the pathophysiology of the disease.^20, 21, 22, 65, 66, 67^ However, the literature remains unsettled regarding these hypotheses.^68, 69^ Since mood and motor activity, food intake, decision-making abilities, reinforcement and reward are features directly affected by disruptions in the serotonin and/or dopamine pathways, we decided to investigate not only these systems but a broader neurotransmitter expression profile in neurons derived from AN patients. No substantial expression changes were found either among serotonin- nor among dopamine-related genes in our culture system. Notably, the expression of *TACR1* was significantly upregulated in AN. This G-protein coupled receptor (GPCR) is distributed in many areas of the human brain including the frontal cortex,^70^ the amygdala and striatum,^71^ and has high affinity for Substance P (SP), the most abundant tachykinin (or neurokinin) that participates in the regulation of a variety of biological functions.^72, 73^

Several studies have reported the contribution of *TACR1* to affective, anxiety and motivated disorders such as addictive behavior,^74^ bipolar disorder,^75, 76, 77^ attention deficit hyperactivity disorder,^75, 78^ depression,^79, 80^ fear^81, 82^ and anxiety.^73, 81, 83, 84, 85^ AN presents as a motivated disorder with anxiety^3^ induced by palatable food and is frequently comorbid with mood symptoms,^3^ especially while the patient is underweight. While *TACR1* has been implicated in a variety of animal models that recapitulate core phenotypes of AN such as heightened anxiety and hyperactivity,^78, 86, 87^ absence of *TACR1* also increases risk for high body mass index in a sex-dependent manner in mice.^88^ Conversely, increased *TACR1* expression in human females with AN presumably increases risk for low body mass index. Among its diverse biological roles, *TACR1* antagonists have been developed to treat emesis and reduction in food intake in those receiving cisplatin chemotherapy.^89^ Mechanistically, TACR1 and SP have been shown to interact with the monoaminergic system^70, 83, 90, 91^ and to modulate the reward pathway.^92, 93^ Notably, the Brainspan atlas show *TACR1* expression peaking during adolescence in the striatum, a brain region implicated in impaired reward processing in AN. The onset of AN is during adolescence and brain imaging studies reveal structural and functional abnormalities in striatal networks.^94^ The pharmacology of reversing phenotypes in TACR1 models and use of dopamine antagonists in the treatment of AN share a common mechanism of targeting the dopaminergic system. Interestingly, a study in *Caenorhabditis elegans* showed that a tachykinin neuroendocrine signalling mediates body fat loss, by connecting the neural serotonin circuit to its metabolic actions in the intestine.^95^ Thus, many aspects of the disease onset and symptoms could be associated with misregulated tackykinins, including the serotonin and dopamine imbalances^78^ observed in AN clinical studies,^19, 21, 22^ especially due to the overlap and functional interaction of monoamines and SP in the brain.^92, 96, 97, 98^

In summary, we have developed a new human cellular model of AN and performed a complete and systematic transcriptional analysis of human AN-derived neurons. Despite using a small cohort, our study identified *TACR1*, a member of the tachykinin family that has previously been associated with other psychiatric disorders, dysregulated in AN. This gene arises as an unexplored target that potentially contributes to the pathophysiology of AN and that might be compromised in patients before other neurotransmitter systems are disrupted. Nonetheless, it is noteworthy that while our approach represents an innovative tool for understanding the molecular and genetic mechanisms contributing to AN, there is a caveat since the reprogramming process can alter the epigenetic memory of cells. If such alterations participate in the disease, they could be underestimated here and should be addressed in future studies. Improvements in the efficacy of current treatments and development of new therapies have been hampered by a lack of knowledge of the neurobiology of AN. Then, identifying novel molecular pathways altered in this complex disorder will provide an opportunity for new diagnostic strategies and treatments. Our study represents a proof-of-concept that AN can be modeled *in vitro.* Although our findings should be confirmed in a broader cohort of patients in the future, we believe that some features attributed to serotonin and/or dopamine pathways could be a consequence of a mis-regulated tachykinin system, ultimately affecting mood, food intake, lipid metabolism, anxiety and reward behaviors in AN individuals.
