# Supplementary figures and images for: Modeling anorexia nervosa: transcriptional insights from human iPSC-derived neurons
Source: Transl Psychiatry. 2017 Mar 14;7(3):e1060–. doi: 10.1038/tp.2017.37 (PMC5416680; doi:10.1038/tp.2017.37)

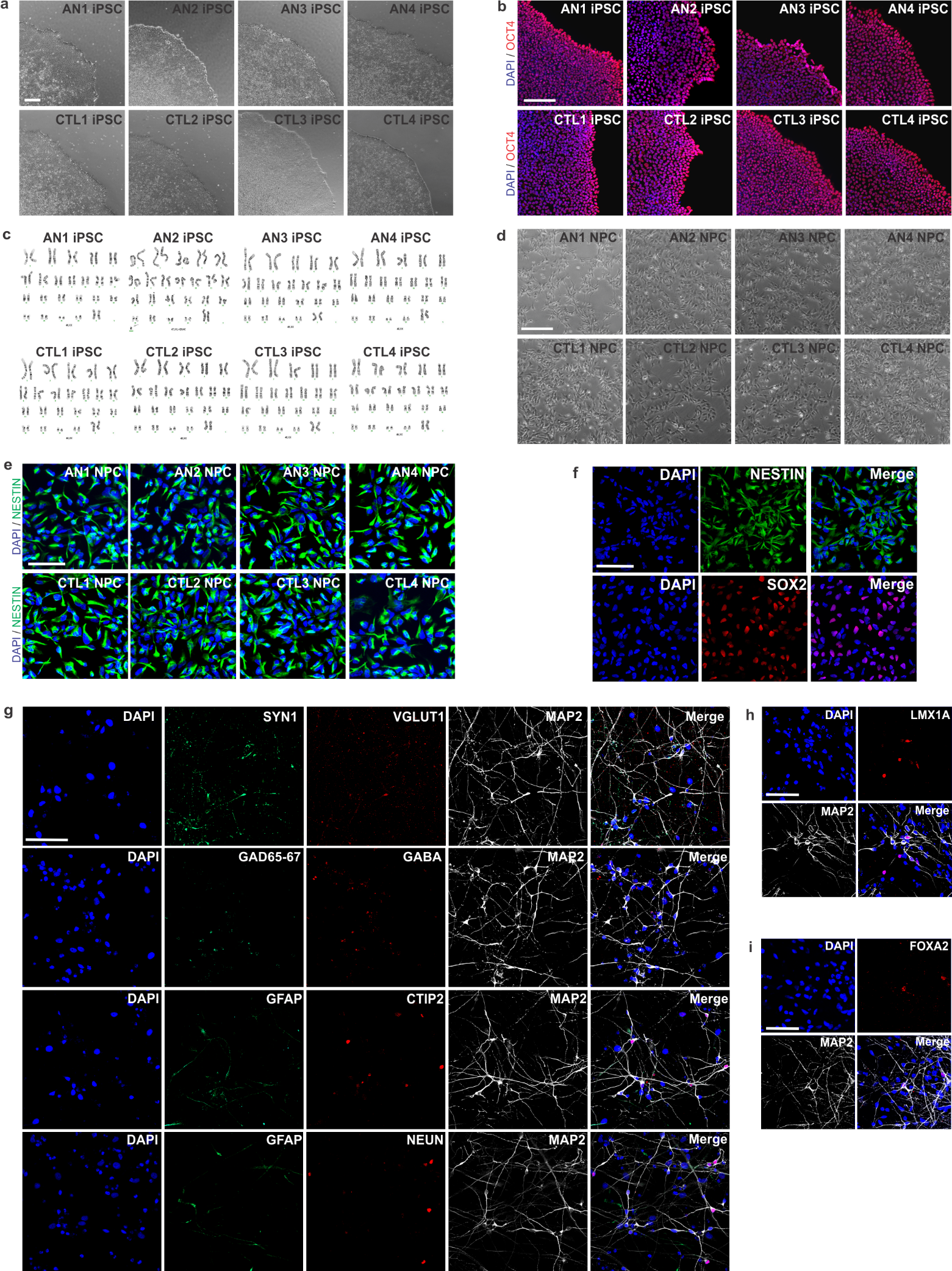


**Supplementary Figure S1** - Negraes et al., 2016


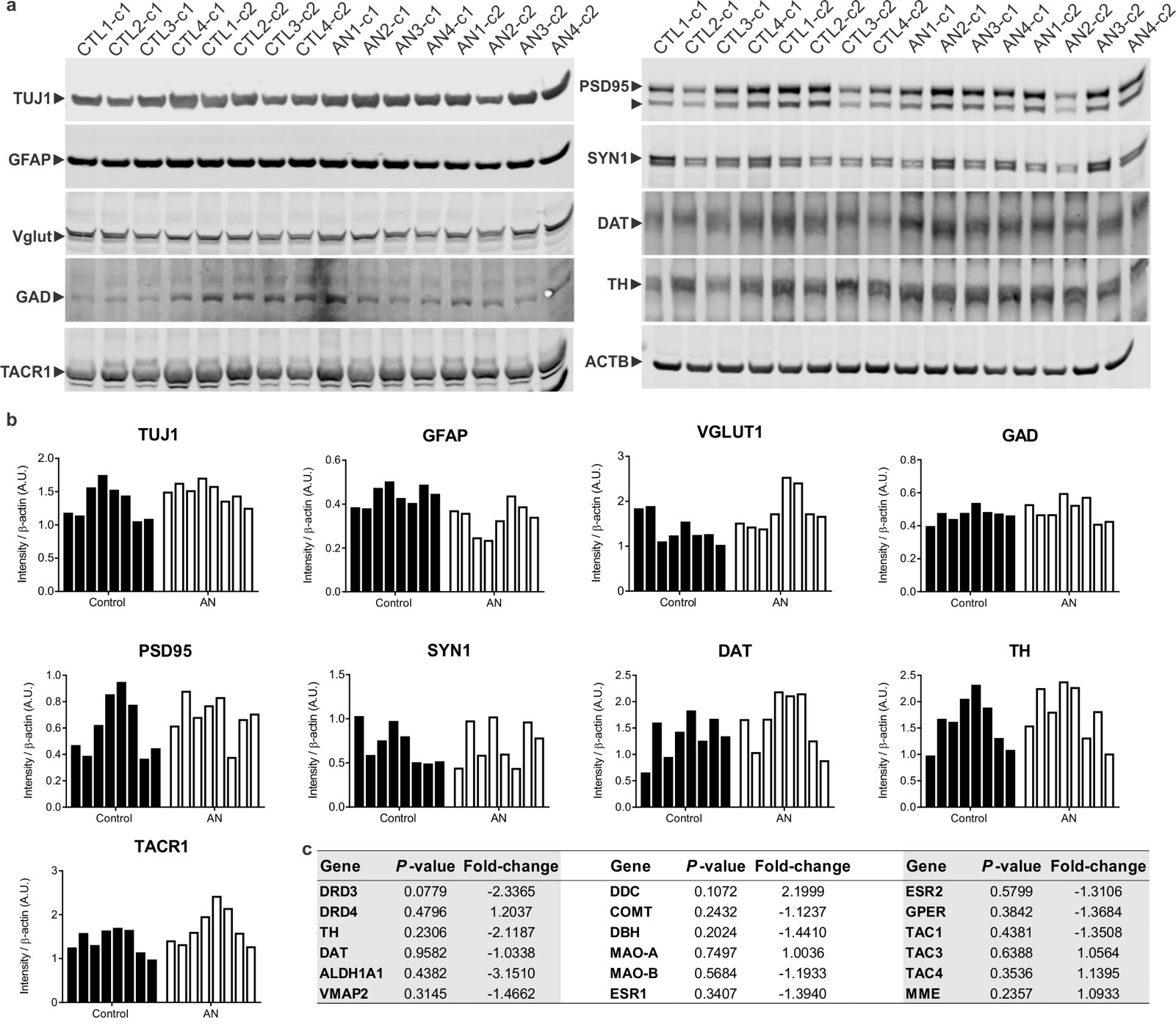


**Supplementary Figure S2** - Negraes et al., 2016

Supplement: Supplementary Figures [file tp201737x1.doc]
