# Supplementary material for: Modeling anorexia nervosa: transcriptional insights from human iPSC-derived neurons
Source: Transl Psychiatry. 2017 Mar 14;7(3):e1060–. doi: 10.1038/tp.2017.37 (PMC5416680; doi:10.1038/tp.2017.37)
Supplement: Supplementary Tables [file tp201737x2.doc]

**Supplementary Table S1.** Primer sequences used for RT-PCR and qRT-PCR gene expression analysis.

| **Gene*** | **Forward Sequence (5' → 3')** | **Reverse Sequence (5' → 3')** |
| --- | --- | --- |
| OCT4 | TCTTTCCACCAGGCCCCCGGCTC | TGCGGGCGGACATGGGGAGATCC |
| NANOG | CAGCCCCGATTCTTCCACCAGTCCC | CGGAAGATTCCCAGTCGGGTTCACC |
| LIN28 | ACGTGCGCATGGGGTTCG | TGGGCCTTCAGCGGACAT |
| AFP | GAATGCTGCAAACTGACCACGCTGGAAC | TGGCATTCAAGAGGGTTTTCAGTCTGGA |
| MSX1 | AGGACCCCGTGGATGCAGAG | GGCCATCTTCAGCTTCTCCAG |
| PAX6 | ACCCATTATCCAGATGTGTTTGCCCGAG | ATGGTGAAGCTGGGCATAGGCGGCAG |
| Mycoplasma | GGCGAATGGGTGAGTAAC | CGGATAACGCTTGCGACCT |
| NESTIN | CTGCTACCCTTGAGACACCTG | GGGCTCTGATCTCTGCATCTAC |
| MAP2 | CTCAGCACCGCTAACAGAGG | CATTGGCGCTTCGGACAAG |
| CTSF | CCCTCCAATGCCTACTCGG | CCAGCTTCTGCTCGTTCTG |
| FAM135B | AGGGTATTACCAGATCCGAGTG | CCTTTCACCACCCAAGAGTAAAT |
| CALCB | ACCCGGCCACACTCAGTAA | GGGCACGAAGTTGCTCTTCA |
| COL22A1 | CCTAGCGTTCGTGTAGAAGGA | CCCATCCGTACATAGGAACTCT |
| CUZD1 | ATGGAGCTTGTAAGAAGGCTCA | GAGTTGCAGGATCATGGCTTT |
| TSHR | TTCCCTGACCTGACCAAAGTT | ACGTCATGTAAGGGTTGTCTGT |
| LPAR3 | GCTGCCGATTTCTTCGCTG | AGCAGTCAAGCTACTGTCCAG |
| DGKG | CCTCGGTCTTCAAGCTCGG | AGAGGCGAAACATGAACTCCA |
| BPIFC | ATCCTCTCAGACCATTTACCCTG | TCAAGAGACTCAGAACCGCTTA |
| CTGF | CAGCATGGACGTTCGTCTG | AACCACGGTTTGGTCCTTGG |
| HABP2 | CACTTACCCACGCTGAGAATC | ACCGTGTTCACAGGGGTTG |
| TDRD10 | TGTATGTTGGCAATCTTCCACTG | ACATCAAGAGGGTTGAAGTCCT |
| FABP12 | TGAAGGAGCTGGGTATAGGAAG | GCTTTCTCGTTATGGTGGTTTCT |
| DRD3 | AGAAGGCAACCCAAATGGTGG | TGTCGTGGCACTGTAAAGCTC |
| DRD4 | CTGTGCTGGACGCCCTTCTTC | TTGAGGGCGCTGTTGACGTAG |
| TH | GGAAGGCCGTGCTAAACCT | GGATTTTGGCTTCAAACGTCTC |
| DAT | TGTGGGCTTCACGGTCATC | GTCCCAAAAGTGTCGTTGAGG |
| ALDH1A1 | GCACGCCAGACTTACCTGTC | CCTCCTCAGTTGCAGGATTAAAG |
| VMAT2 | CGGAAGCTCATCCTGTTCATC | CCTGGCCGTCTGGATTTCTG |
| DDC | ATTCATCTGCCCTGAGTTCCG | CCAATAGCCATTTGTGGGGAT |
| COMT | TACTGCGAGCAGAAGGAGTG | CCAGCGAAATCCACCATCC |
| DBH | TTACAACAAGTCTCTCTCGTCCT | GGCTGCCGATGAAGTGGTA |
| MAO-A | GAATCAAGAGAAGGCGAGTATCG | GGCAGCAGATAGTCCTGAAATG |
| MAO-B | GGAGCTAGGATTGGAGACCTAC | CCCTGAAGGGGTATGATTTGC |
| ESR1 | GGGAAGTATGGCTATGGAATCTG | TGGCTGGACACATATAGTCGTT |
| ESR2 | TCCATCGCCAGTTATCACATCT | CTGGACCAGTAACAGGGCTG |
| GPER | CACCAGCAGTACGTGATCGG | CATCTTCTCGCGGAAGCTGAT |
| TAC1 | TGATCTGAATTACTGGTCCGACT | TCCGGCAGTTCCTCCTTGA |
| TAC3 | ATGCTGCTATTCACAGCCATC | GGCTCCTTACAGACAGCCC |
| TAC4 | ACCTGGGTAATTGTGGCCTTG | CCTTCTGTGAACAGGCTTCTCTT |
| MME | GATCGCACTCTATGCAACCTAC | TGTTTTGGATCAGTCGAGCAG |
| B2M | GAGGCTATCCAGCGTACTCCA | CGGCAGGCATACTCATCTTTT |
| GAPDH | CATGAGAAGTATGACAACAGCCT | AGTCCTTCCACGATACCAAAGT |

*PCR Array Catalog number PAHS-060Z (Qiagen)

**Supplementary Table S2.** Antibodies used in this study.

| **Antibody** | **Dilution** | **Manufacturer** |
| --- | --- | --- |
| OCT4 | 1:500 | Abcam |
| NANOG | 1:500 | R&D Systems |
| TRA-1-60 | 1:250 | Abcam |
| LIN28 | 1:500 | Abcam |
| NESTIN | 1:500 | Millipore |
| SOX2 | 1:250 | Cell Signaling |
| MAP2 | 1:2000; 1:3000* | Abcam; Sigma* |
| SYN1 | 1:500; 1:1500* | Millipore |
| VGLUT1 | 1:500 | Synaptic Systems |
| NEUN | 1:500 | Millipore |
| CTIP2 | 1:500 | Abcam |
| GAD65-67 | 1:500; 1:1500* | Abcam |
| GABA | 1:250 | Abcam |
| LMX1A | 1:500 | Millipore |
| FOXA2 | 1:100 | Santa Cruz |
| GFAP | 1:2000; 1:3000* | Dako |
| PSD95 | 1:1500* | NeuroMab |
| TACR1 | 1:1000* | Assay Biotechnology |
| TUJ1 | 1:3000* | Covance |
| TH | 1:500* | Aviva Systems Biology |
| DAT | 1:500* | Everest Biotech |
| Β-ACTIN | 1:5000* | Abcam |

*Western Blot

**Supplementary Table S3.** Summary of cell lines used in each experiment (fibroblasts, iPSC, iPSC-derived NPC and neurons).

| **Experiments** | **AN1** | | **AN2** | | **AN3** | | **AN4** | | **AN5** | **AN6** | **AN7** | **CTL1** | | **CTL2** | | **CTL3** | | **CTL4** | |
| --- | --- | --- | --- | --- | --- | --- | --- | --- | --- | --- | --- | --- | --- | --- | --- | --- | --- | --- | --- |
|  | **c1** | **c2** | **c1** | **c2** | **c1** | **c2** | **c1** | **c2** |  |  |  | **c1** | **c2** | **c1** | **c2** | **c1** | **c2** | **c1** | **c2** |
| Fibroblast |  |  |  |  |  |  |  |  |  |  |  |  |  |  |  |  |  |  |  |
| Karyotyping | Y | | Y | | Y | | Y | | Y | Y | Y | Y | | Y | | Y | | Y | |
| RNA sequencing | Y | | Y | | Y | | Y | | Y | Y | Y | Y | | Y | | Y | | Y | |
| iPSC characterization |  |  |  |  |  |  |  |  |  |  |  |  |  |  |  |  |  |  |  |
| Immunostaining | Y | Y | Y | Y | Y | Y | Y | Y |  |  |  | Y | Y | Y | Y | Y | Y | Y | Y |
| RT-PCR | Y | Y |  | Y | Y |  | Y |  |  |  |  | Y | Y | Y |  | Y |  | Y |  |
| Teratoma assay | Y | Y | Y | Y | Y | Y | Y | Y |  |  |  | Y | Y | Y | Y | Y | Y | Y | Y |
| Karyotyping | Y | Y | Y | Y | Y | Y | Y | Y | Y | Y | Y | Y | Y | Y | Y | Y | Y | Y | Y |
| RNA sequencing | Y | Y | Y | Y | Y |  |  |  |  |  |  | Y | Y | Y | Y |  |  |  |  |
| NPC characterization |  |  |  |  |  |  |  |  |  |  |  |  |  |  |  |  |  |  |  |
| Immunostaining | Y | Y | Y | Y | Y | Y | Y | Y |  |  |  | Y | Y | Y | Y | Y | Y | Y | Y |
| RT-qPCR | Y | Y | Y | Y | Y | Y | Y | Y |  |  |  | Y | Y | Y | Y | Y | Y | Y | Y |
| Neuronal characterization |  |  |  |  |  |  |  |  |  |  |  |  |  |  |  |  |  |  |  |
| Immunostaining | Y | Y | Y | Y | Y | Y | Y | Y |  |  |  | Y | Y | Y | Y | Y | Y | Y | Y |
| qPCR Array | Y | Y | Y | Y | Y |  | Y |  |  |  |  | Y | Y | Y | Y | Y |  | Y |  |
| RT-qPCR | Y | Y | Y | Y | Y | Y | Y | Y |  |  |  | Y | Y | Y | Y | Y | Y | Y | Y |
| RNA sequencing | Y | Y | Y | Y | Y |  |  |  |  |  |  | Y | Y | Y | Y |  |  |  |  |
| RNA-seq validation (RT-qPCR) | Y | Y | Y | Y | Y |  | Y |  |  |  |  | Y | Y | Y | Y | Y |  | Y |  |
| Number of differentiations | 8 | 7 | 6 | 8 | 7 | 4 | 5 | 4 |  |  |  | 5 | 8 | 6 | 7 | 5 | 4 | 4 | 4 |

c#; iPSC clone number. Y; specified clone was used for specified experiments.

**Supplementary Table S4.** Pluripotency-related genes and corresponding splicing isoforms.

| **Ensembl Gene ID** | **Associated Gene Name** | **Description** | **Isoform Transcript ID** | **Band** |
| --- | --- | --- | --- | --- |
| ENSG00000111704 | NANOG | Nanog homeobox [Source:HGNC Symbol;Acc:HGNC:20857] | ENST00000229307 | p13.31 |
| ENSG00000131914 | LIN28A | lin-28 homolog A (C. elegans) [Source:HGNC Symbol;Acc:HGNC:15986] | ENST00000254231 | p36.11 |
| ENSG00000204531 | POU5F1 | POU class 5 homeobox 1 [Source:HGNC Symbol;Acc:HGNC:9221] | ENST00000259915 | p21.33 |
| ENSG00000128567 | PODXL | podocalyxin-like [Source:HGNC Symbol;Acc:HGNC:9171] | ENST00000322985 | q32.3 |
| ENSG00000181449 | SOX2 | SRY (sex determining region Y)-box 2 [Source:HGNC Symbol;Acc:HGNC:11195] | ENST00000325404 | q26.33 |
| ENSG00000131914 | LIN28A | lin-28 homolog A (C. elegans) [Source:HGNC Symbol;Acc:HGNC:15986] | ENST00000326279 | p36.11 |
| ENSG00000184344 | GDF3 | growth differentiation factor 3 [Source:HGNC Symbol;Acc:HGNC:4218] | ENST00000329913 | p13.31 |
| ENSG00000203909 | DPPA5 | developmental pluripotency associated 5 [Source:HGNC Symbol;Acc:HGNC:19201] | ENST00000370370 | q13 |
| ENSG00000128567 | PODXL | podocalyxin-like [Source:HGNC Symbol;Acc:HGNC:9171] | ENST00000378555 | q32.3 |
| ENSG00000204531 | POU5F1 | POU class 5 homeobox 1 [Source:HGNC Symbol;Acc:HGNC:9221] | ENST00000441888 | p21.33 |
| ENSG00000128567 | PODXL | podocalyxin-like [Source:HGNC Symbol;Acc:HGNC:9171] | ENST00000446198 | q32.3 |
| ENSG00000204531 | POU5F1 | POU class 5 homeobox 1 [Source:HGNC Symbol;Acc:HGNC:9221] | ENST00000461401 | p21.33 |
| ENSG00000128567 | PODXL | podocalyxin-like [Source:HGNC Symbol;Acc:HGNC:9171] | ENST00000465001 | q32.3 |
| ENSG00000204531 | POU5F1 | POU class 5 homeobox 1 [Source:HGNC Symbol;Acc:HGNC:9221] | ENST00000471529 | p21.33 |
| ENSG00000163530 | DPPA2 | developmental pluripotency associated 2 [Source:HGNC Symbol;Acc:HGNC:19197] | ENST00000478945 | q13.13 |
| ENSG00000128567 | PODXL | podocalyxin-like [Source:HGNC Symbol;Acc:HGNC:9171] | ENST00000482581 | q32.3 |
| ENSG00000128567 | PODXL | podocalyxin-like [Source:HGNC Symbol;Acc:HGNC:9171] | ENST00000484346 | q32.3 |
| ENSG00000128567 | PODXL | podocalyxin-like [Source:HGNC Symbol;Acc:HGNC:9171] | ENST00000487965 | q32.3 |
| ENSG00000128567 | PODXL | podocalyxin-like [Source:HGNC Symbol;Acc:HGNC:9171] | ENST00000490761 | q32.3 |
| ENSG00000204531 | POU5F1 | POU class 5 homeobox 1 [Source:HGNC Symbol;Acc:HGNC:9221] | ENST00000512818 | p21.33 |
| ENSG00000204531 | POU5F1 | POU class 5 homeobox 1 [Source:HGNC Symbol;Acc:HGNC:9221] | ENST00000513407 | p21.33 |
| ENSG00000111704 | NANOG | Nanog homeobox [Source:HGNC Symbol;Acc:HGNC:20857] | ENST00000526286 | p13.31 |
| ENSG00000111704 | NANOG | Nanog homeobox [Source:HGNC Symbol;Acc:HGNC:20857] | ENST00000526434 | p13.31 |
| ENSG00000111704 | NANOG | Nanog homeobox [Source:HGNC Symbol;Acc:HGNC:20857] | ENST00000541267 | p13.31 |

**Supplementary Table S5.** Differential gene expression annotated by HGNC and ENSEMBL for pathway analysis.

| **Ensembl ID** | **Symbol** | **Description** | **Locus**  **Group** | **Expression in**  **control** | **Expression in AN** | **Fold-Change** | **Log2 Fold-Change** | ***P*-value** |
| --- | --- | --- | --- | --- | --- | --- | --- | --- |
| ENSG00000223484 | TRPC6P | transient receptor potential cation channel, subfamily C, member 6 pseudogene | pseudogene | 0 | 3.463679452 | Inf | Inf | 0.015218044 |
| ENSG00000251957 | RNU6-1095P | RNA, U6 small nuclear 1095, pseudogene | pseudogene | 0 | 3.320734097 | Inf | Inf | 0.021302157 |
| ENSG00000151365 | THRSP | thyroid hormone responsive | protein-coding gene | 0 | 2.828603402 | Inf | Inf | 0.036240234 |
| ENSG00000185290 | NUPR1L | nuclear protein 2, transcriptional regulator | protein-coding gene | 9.833845409 | 0 | 0 | -Inf | 0.006376876 |
| ENSG00000235315 | RPL23AP69 | ribosomal protein L23a pseudogene 69 | pseudogene | 3.586494966 | 0 | 0 | -Inf | 0.010147736 |
| ENSG00000205488 | CALML3-AS1 | CALML3 antisense RNA 1 | non-coding RNA | 2.977907309 | 0 | 0 | -Inf | 0.020921536 |
| ENSG00000252982 | RN7SKP234 | RNA, 7SK small nuclear pseudogene 234 | pseudogene | 2.644234917 | 0 | 0 | -Inf | 0.030724357 |
| ENSG00000266010 | GATA6-AS1 | GATA6 antisense RNA 1 (head to head) | non-coding RNA | 9.672935374 | 0 | 0 | -Inf | 0.042373015 |
| ENSG00000174080 | CTSF | cathepsin F | protein-coding gene | 1.304443465 | 561.2440446 | 430.255553 | 8.749050002 | 0.001514888 |
| ENSG00000147724 | FAM135B | family with sequence similarity 135 member B | protein-coding gene | 5.355945952 | 1019.295681 | 190.3110468 | 7.572215497 | 2.51751E-09 |
| ENSG00000053438 | NNAT | neuronatin | protein-coding gene | 257.4962239 | 36651.90372 | 142.3395775 | 7.153193049 | 0.019222626 |
| ENSG00000175868 | CALCB | calcitonin-related polypeptide beta | protein-coding gene | 0.635621025 | 44.51751355 | 70.03782414 | 6.130062359 | 0.002391262 |
| ENSG00000134463 | ECHDC3 | enoyl-CoA hydratase domain containing 3 | protein-coding gene | 0.531723636 | 12.88395774 | 24.23055299 | 4.598755426 | 0.018005827 |
| ENSG00000138161 | CUZD1 | CUB and zona pellucida-like domains 1 | protein-coding gene | 1.343870349 | 31.22806837 | 23.23741154 | 4.538377468 | 2.67031E-05 |
| ENSG00000088320 | REM1 | RAS (RAD and GEM)-like GTP-binding 1 | protein-coding gene | 0.319849961 | 6.660861739 | 20.82495716 | 4.380241623 | 0.01161569 |
| ENSG00000169436 | COL22A1 | collagen, type XXII, alpha 1 | protein-coding gene | 34.57390112 | 609.1195691 | 17.61790106 | 4.138970151 | 0.007876202 |
| ENSG00000157211 | CDCP2 | CUB domain containing protein 2 | protein-coding gene | 0.246148376 | 4.288400038 | 17.42201231 | 4.122839365 | 0.018071215 |
| ENSG00000240184 | PCDHGC3 | protocadherin gamma subfamily C, 3 | other | 56.32550255 | 892.2043157 | 15.84014834 | 3.985513941 | 2.0773E-11 |
| ENSG00000207457 | RNU6-476P | RNA, U6 small nuclear 476, pseudogene | pseudogene | 0.458022051 | 6.87194063 | 15.00351482 | 3.90722861 | 0.004051511 |
| ENSG00000247516 | MIR4458HG | MIR4458 host gene | non-coding RNA | 15.16245411 | 173.0237163 | 11.41132663 | 3.512394617 | 4.44717E-11 |
| ENSG00000171517 | LPAR3 | lysophosphatidic acid receptor 3 | protein-coding gene | 8.784633979 | 95.15179397 | 10.83161737 | 3.437176776 | 0.002624963 |
| ENSG00000101460 | MAP1LC3A | microtubule associated protein 1 light chain 3 alpha | protein-coding gene | 22.15723358 | 232.2665267 | 10.4826501 | 3.389931583 | 0.011453076 |
| ENSG00000181234 | TMEM132C | transmembrane protein 132C | protein-coding gene | 279.4478845 | 2876.372345 | 10.29305464 | 3.363599285 | 0.000403052 |
| ENSG00000182489 | XKRX | X-linked Kx blood group related, X-linked | protein-coding gene | 2.267155785 | 23.14755061 | 10.2099515 | 3.351904108 | 0.002666228 |
| ENSG00000216863 | LY86-AS1 | LY86 antisense RNA 1 | non-coding RNA | 0.492296752 | 4.711209748 | 9.569857465 | 3.258497437 | 0.034437947 |
| ENSG00000266853 | ITM2BP1 | integral membrane protein 2B pseudogene 1 | pseudogene | 0.498621623 | 4.755753585 | 9.537800531 | 3.253656611 | 0.033921781 |
| ENSG00000125144 | MT1G | metallothionein 1G | protein-coding gene | 0.884931836 | 8.009067181 | 9.050490503 | 3.177995983 | 0.043830348 |
| ENSG00000152093 | CFC1B | cripto, FRL-1, cryptic family 1B | protein-coding gene | 9.566072074 | 85.80836374 | 8.970072886 | 3.165119708 | 7.30208E-05 |
| ENSG00000134330 | IAH1 | isoamyl acetate-hydrolyzing esterase 1 homolog | protein-coding gene | 35.35602248 | 307.3337 | 8.692541707 | 3.119778084 | 0.01290254 |
| ENSG00000081041 | CXCL2 | chemokine (C-X-C motif) ligand 2 | protein-coding gene | 1.480052692 | 12.53802241 | 8.471335161 | 3.08258937 | 0.021717388 |
| ENSG00000249267 | LINC00939 | long intergenic non-protein coding RNA 939 | non-coding RNA | 1.096805511 | 9.010579306 | 8.215293608 | 3.038312137 | 0.040387661 |
| ENSG00000149021 | SCGB1A1 | secretoglobin, family 1A, member 1 (uteroglobin) | protein-coding gene | 9.234745059 | 74.39731518 | 8.056239204 | 3.01010652 | 0.021913255 |
| ENSG00000165970 | SLC6A5 | solute carrier family 6 (neurotransmitter transporter), member 5 | protein-coding gene | 535.566389 | 4223.615076 | 7.886258665 | 2.979341031 | 0.014373032 |
| ENSG00000212396 | RNA5SP323 | RNA, 5S ribosomal pseudogene 323 | pseudogene | 0.569160773 | 4.38963451 | 7.71246846 | 2.947192684 | 0.035440332 |
| ENSG00000265787 | CYP4F35P | cytochrome P450, family 4, subfamily F, polypeptide 35, pseudogene | pseudogene | 0.569160773 | 4.242525216 | 7.454001431 | 2.898015096 | 0.049280806 |
| ENSG00000214263 | RPSAP53 | ribosomal protein SA pseudogene 53 | pseudogene | 32.81778242 | 232.48972 | 7.08426051 | 2.824617265 | 0.011925405 |
| ENSG00000106038 | EVX1 | even-skipped homeobox 1 | protein-coding gene | 9.741889147 | 68.24175424 | 7.004981601 | 2.808381261 | 0.023922803 |
| ENSG00000165409 | TSHR | thyroid stimulating hormone receptor | protein-coding gene | 33.02674385 | 229.413534 | 6.946295859 | 2.79624386 | 0.000213845 |
| ENSG00000186481 | ANKRD20A5P | ankyrin repeat domain 20 family member A5, pseudogene | pseudogene | 4.154226824 | 28.85460781 | 6.94584312 | 2.796149826 | 0.00098959 |
| ENSG00000127412 | TRPV5 | transient receptor potential cation channel, subfamily V, member 5 | protein-coding gene | 1.162192478 | 7.501558788 | 6.45466128 | 2.69034139 | 0.025836413 |
| ENSG00000180221 | TPT1P10 | tumor protein, translationally-controlled 1 pseudogene 10 | pseudogene | 0.639699922 | 4.116980786 | 6.43580004 | 2.686119502 | 0.044861096 |
| ENSG00000232818 | RPS2P32 | ribosomal protein S2 pseudogene 32 | pseudogene | 4.059817105 | 25.43101537 | 6.264079075 | 2.647102425 | 0.003529206 |
| ENSG00000233265 | MICF | MHC class I polypeptide-related sequence F (pseudogene) | pseudogene | 1.378145051 | 8.605256393 | 6.244086128 | 2.642490437 | 0.01099829 |
| ENSG00000181965 | NEUROG1 | neurogenin 1 | protein-coding gene | 90.23638257 | 560.3065891 | 6.209320156 | 2.63443532 | 0.032582824 |
| ENSG00000139865 | TTC6 | tetratricopeptide repeat domain 6 | protein-coding gene | 29.33041822 | 180.7568699 | 6.16277847 | 2.623580932 | 0.001212996 |
| ENSG00000184937 | WT1 | Wilms tumor 1 | protein-coding gene | 16.98180148 | 103.2675713 | 6.081072814 | 2.604325864 | 0.012496026 |
| ENSG00000160180 | TFF3 | trefoil factor 3 | protein-coding gene | 53.19090635 | 314.8659899 | 5.919545492 | 2.565486409 | 0.004750455 |
| ENSG00000221870 | TMEM257 | transmembrane protein 257 | protein-coding gene | 26.40759388 | 152.9742378 | 5.79281242 | 2.534263949 | 0.000109933 |
| ENSG00000230068 | CDC42-IT1 | CDC42 intronic transcript 1 | non-coding RNA | 1.134242648 | 6.450030141 | 5.686640466 | 2.507576594 | 0.035295777 |
| ENSG00000140968 | IRF8 | interferon regulatory factor 8 | protein-coding gene | 6.359109699 | 35.5393034 | 5.588723121 | 2.482518702 | 0.042360514 |
| ENSG00000109851 | DBX1 | developing brain homeobox 1 | protein-coding gene | 78.39730293 | 415.3060705 | 5.297453547 | 2.405299032 | 0.026789473 |
| ENSG00000232608 | TIMM9P2 | TIMM9 pseudogene 2 | pseudogene | 1.770780135 | 9.223264186 | 5.208588014 | 2.380892328 | 0.014434127 |
| ENSG00000167711 | SERPINF2 | serpin peptidase inhibitor, clade F (alpha-2 antiplasmin, pigment epithelium derived factor), member 2 | protein-coding gene | 20.02284039 | 103.7502581 | 5.181595421 | 2.373396374 | 0.010821316 |
| ENSG00000058866 | DGKG | diacylglycerol kinase gamma | protein-coding gene | 76.10909957 | 369.8420973 | 4.859367663 | 2.280768592 | 0.002459868 |
| ENSG00000202515 | VTRNA1-3 | vault RNA 1-3 | non-coding RNA | 1.707738545 | 8.255209436 | 4.834000766 | 2.273217702 | 0.029436538 |
| ENSG00000125618 | PAX8 | paired box 8 | protein-coding gene | 82.65998072 | 392.7873409 | 4.75184409 | 2.248487502 | 0.006062569 |
| ENSG00000183242 | WT1-AS | WT1 antisense RNA | non-coding RNA | 5.33457722 | 24.96574214 | 4.679985145 | 2.22650395 | 0.049666322 |
| ENSG00000199545 | RNA5SP195 | RNA, 5S ribosomal pseudogene 195 | pseudogene | 1.630618298 | 7.5900021 | 4.654677376 | 2.218681175 | 0.031429379 |
| ENSG00000231258 | ZSWIM5P2 | zinc finger, SWIM-type containing 5 pseudogene 2 | pseudogene | 1.51631714 | 7.031127478 | 4.63697685 | 2.213184524 | 0.038699263 |
| ENSG00000129514 | FOXA1 | forkhead box A1 | protein-coding gene | 270.5854357 | 1242.550722 | 4.592082786 | 2.199148651 | 0.037308714 |
| ENSG00000243970 | PPIEL | peptidylprolyl isomerase E-like pseudogene | pseudogene | 13.18884729 | 60.13365314 | 4.559432059 | 2.188854128 | 0.021783573 |
| ENSG00000214688 | C10orf105 | chromosome 10 open reading frame 105 | protein-coding gene | 27.34722202 | 123.1382899 | 4.502771424 | 2.170813244 | 0.00012558 |
| ENSG00000198734 | F5 | coagulation factor V (proaccelerin, labile factor) | protein-coding gene | 117.2226954 | 521.4897804 | 4.448710025 | 2.153387064 | 0.042939907 |
| ENSG00000136698 | CFC1 | cripto, FRL-1, cryptic family 1 | protein-coding gene | 24.43336313 | 108.4908796 | 4.440276152 | 2.150649404 | 0.01189628 |
| ENSG00000164530 | PI16 | peptidase inhibitor 16 | protein-coding gene | 163.5947402 | 690.8960239 | 4.223216608 | 2.078342245 | 0.022816384 |
| ENSG00000171540 | OTP | orthopedia homeobox | protein-coding gene | 463.0035178 | 1943.290604 | 4.197140041 | 2.069406601 | 0.031613775 |
| ENSG00000231459 | LINC00032 | long intergenic non-protein coding RNA 32 | non-coding RNA | 4.309127916 | 18.03789324 | 4.18597303 | 2.065563017 | 0.029448607 |
| ENSG00000233670 | PIRT | phosphoinositide-interacting regulator of transient receptor potential channels | protein-coding gene | 12.41055583 | 50.92924911 | 4.103704122 | 2.036926716 | 0.049432583 |
| ENSG00000189398 | OR7E12P | olfactory receptor, family 7, subfamily E, member 12 pseudogene | pseudogene | 6.903531819 | 28.25875364 | 4.093376315 | 2.033291304 | 0.002560858 |
| ENSG00000116157 | GPX7 | glutathione peroxidase 7 | protein-coding gene | 26.79616851 | 106.6277495 | 3.979216263 | 1.992484309 | 0.033548321 |
| ENSG00000232894 | MRPS31P2 | mitochondrial ribosomal protein S31 pseudogene 2 | pseudogene | 2.226812439 | 8.466119985 | 3.801900797 | 1.926720888 | 0.040077244 |
| ENSG00000237917 | PARP4P1 | poly(ADP-ribose) polymerase family member 4 pseudogene 1 | pseudogene | 4.379923292 | 16.57643192 | 3.784639778 | 1.920155991 | 0.009863625 |
| ENSG00000187144 | SPATA21 | spermatogenesis associated 21 | protein-coding gene | 2.057528083 | 7.685446886 | 3.735281646 | 1.901217029 | 0.049330659 |
| ENSG00000228663 | PSMD10P1 | proteasome 26S subunit, non-ATPase, 10 pseudogene 1 | pseudogene | 3.824229321 | 14.12889134 | 3.694572202 | 1.885407323 | 0.021488976 |
| ENSG00000249834 | PGBD4P3 | piggyBac transposable element derived 4 pseudogene 3 | pseudogene | 3.900020056 | 14.35098137 | 3.679719889 | 1.879595948 | 0.012831887 |
| ENSG00000196092 | PAX5 | paired box 5 | protein-coding gene | 246.3646723 | 905.8384484 | 3.67681957 | 1.878458382 | 0.037311443 |
| ENSG00000146858 | ZC3HAV1L | zinc finger CCCH-type, antiviral 1-like | protein-coding gene | 45.74239111 | 165.6387722 | 3.621121857 | 1.856436727 | 0.014978697 |
| ENSG00000142698 | C1orf94 | chromosome 1 open reading frame 94 | protein-coding gene | 11.71750719 | 42.18422534 | 3.600102365 | 1.848037928 | 0.023160185 |
| ENSG00000252755 | RNU6-703P | RNA, U6 small nuclear 703, pseudogene | pseudogene | 3.323361724 | 11.68215522 | 3.515162112 | 1.813591225 | 0.024835171 |
| ENSG00000127903 | ZNF835 | zinc finger protein 835 | protein-coding gene | 98.06923514 | 340.3939369 | 3.470955355 | 1.795332809 | 0.006025298 |
| ENSG00000231688 | RPL21P43 | ribosomal protein L21 pseudogene 43 | pseudogene | 2.510141725 | 8.682362166 | 3.458913128 | 1.79031878 | 0.041152721 |
| ENSG00000207234 | RNU6-125P | RNA, U6 small nuclear 125, pseudogene | pseudogene | 3.805353748 | 12.88381012 | 3.385706289 | 1.759456825 | 0.016864618 |
| ENSG00000241484 | ARHGAP8 | Rho GTPase activating protein 8 | protein-coding gene | 17.72922147 | 59.95131559 | 3.38149736 | 1.757662227 | 0.01040525 |
| ENSG00000108439 | PNPO | pyridoxamine 5'-phosphate oxidase | protein-coding gene | 72.5876134 | 243.0551476 | 3.348438338 | 1.743488401 | 0.007865248 |
| ENSG00000120903 | CHRNA2 | cholinergic receptor, nicotinic alpha 2 | protein-coding gene | 42.60749584 | 142.0610853 | 3.334180583 | 1.737332244 | 0.032042367 |
| ENSG00000154646 | TMPRSS15 | transmembrane protease, serine 15 | protein-coding gene | 31.08402106 | 101.3436114 | 3.260312145 | 1.705010096 | 0.012984892 |
| ENSG00000177133 | LINC00982 | long intergenic non-protein coding RNA 982 | non-coding RNA | 93.24679245 | 299.8774958 | 3.215955079 | 1.685247255 | 0.022734984 |
| ENSG00000229465 | ACTG1P11 | actin gamma 1 pseudogene 11 | pseudogene | 8.306572345 | 25.9130335 | 3.119581992 | 1.641352728 | 0.012067221 |
| ENSG00000179855 | GIPC3 | GIPC PDZ domain containing family member 3 | protein-coding gene | 4.748687444 | 14.56325833 | 3.066796563 | 1.616732468 | 0.024674319 |
| ENSG00000183837 | PNMA3 | paraneoplastic Ma antigen 3 | protein-coding gene | 292.0139902 | 887.3689496 | 3.038789165 | 1.603496582 | 0.021483558 |
| ENSG00000095777 | MYO3A | myosin IIIA | protein-coding gene | 79.88364009 | 237.4279714 | 2.972172665 | 1.57151793 | 0.010956035 |
| ENSG00000204805 | FAM27E4 | family with sequence similarity 27 member E4 | non-coding RNA | 25.18151957 | 74.60344896 | 2.962626967 | 1.566876984 | 0.004106621 |
| ENSG00000237945 | LINC00649 | long intergenic non-protein coding RNA 649 | non-coding RNA | 120.6530652 | 356.5831337 | 2.955441979 | 1.563373898 | 0.002805001 |
| ENSG00000213394 | RP4-747G18.5 | ribosomal protein SA pseudogene 46 | pseudogene | 6.868596882 | 19.29396481 | 2.80901109 | 1.49006232 | 0.027213676 |
| ENSG00000105427 | CNFN | cornifelin | protein-coding gene | 6.447755741 | 18.04682027 | 2.798930511 | 1.48487567 | 0.041184948 |
| ENSG00000174680 | GRIK1-AS1 | GRIK1 antisense RNA 1 | non-coding RNA | 7.038541473 | 19.64040315 | 2.790408101 | 1.480476134 | 0.024712459 |
| ENSG00000093072 | CECR1 | cat eye syndrome chromosome region, candidate 1 | protein-coding gene | 288.4062241 | 802.6380504 | 2.783012236 | 1.476647251 | 0.016109782 |
| ENSG00000162623 | TYW3 | tRNA-yW synthesizing protein 3 homolog | protein-coding gene | 213.3532128 | 593.6760646 | 2.782597257 | 1.476432113 | 0.006447986 |
| ENSG00000125804 | FAM182A | family with sequence similarity 182 member A | other | 51.67690913 | 143.6506436 | 2.779783969 | 1.474972768 | 0.030349519 |
| ENSG00000185985 | SLITRK2 | SLIT and NTRK like family member 2 | protein-coding gene | 1471.821744 | 4018.238583 | 2.730112257 | 1.448960273 | 0.001484301 |
| ENSG00000214289 | RPL39P5 | ribosomal protein L39 pseudogene 5 | pseudogene | 8.296572586 | 22.53628408 | 2.716336638 | 1.441662285 | 0.022605019 |
| ENSG00000248668 | OXCT1-AS1 | OXCT1 antisense RNA 1 | non-coding RNA | 11.7654475 | 31.7759964 | 2.700789443 | 1.43338117 | 0.014026229 |
| ENSG00000175772 | LINC01106 | long intergenic non-protein coding RNA 1106 | non-coding RNA | 9.129014746 | 24.40320002 | 2.673147179 | 1.418539272 | 0.019616486 |
| ENSG00000116791 | CRYZ | crystallin zeta | protein-coding gene | 415.9184037 | 1103.32662 | 2.652747774 | 1.407487509 | 0.023100333 |
| ENSG00000159648 | TEPP | testis, prostate and placenta expressed | protein-coding gene | 25.94612286 | 68.11169845 | 2.625120478 | 1.392383636 | 0.029646088 |
| ENSG00000128242 | GAL3ST1 | galactose-3-O-sulfotransferase 1 | protein-coding gene | 16.5155635 | 43.27871017 | 2.620480384 | 1.38983131 | 0.008090516 |
| ENSG00000239265 | CLRN1-AS1 | CLRN1 antisense RNA 1 | non-coding RNA | 6.828500722 | 17.82643857 | 2.610593349 | 1.384377747 | 0.045456793 |
| ENSG00000230002 | ALMS1-IT1 | ALMS1 intronic transcript 1 | non-coding RNA | 12.32028206 | 31.97435762 | 2.595261817 | 1.375880089 | 0.027294119 |
| ENSG00000140479 | PCSK6 | proprotein convertase subtilisin/kexin type 6 | protein-coding gene | 114.8525647 | 290.1450181 | 2.526238912 | 1.336991084 | 0.031456883 |
| ENSG00000172748 | ZNF596 | zinc finger protein 596 | protein-coding gene | 192.3833856 | 485.7431693 | 2.524870678 | 1.336209496 | 0.043167374 |
| ENSG00000204588 | LINC01123 | long intergenic non-protein coding RNA 1123 | non-coding RNA | 9.349302805 | 23.41909669 | 2.504903005 | 1.32475474 | 0.049893522 |
| ENSG00000237550 | UBE2Q2P6 | ribosomal protein L9 pseudogene 9 | pseudogene | 2125.391381 | 5305.825879 | 2.496399452 | 1.3198488 | 0.005569897 |
| ENSG00000197921 | HES5 | hes family bHLH transcription factor 5 | protein-coding gene | 705.7961418 | 1750.357007 | 2.479975313 | 1.310325759 | 0.01510799 |
| ENSG00000164161 | HHIP | hedgehog interacting protein | protein-coding gene | 726.5687387 | 1795.199956 | 2.470791626 | 1.304973346 | 0.025458176 |
| ENSG00000223508 | RPL23AP53 | ribosomal protein L23a pseudogene 53 | pseudogene | 63.49906127 | 156.6369623 | 2.466760282 | 1.302617523 | 0.008014409 |
| ENSG00000264109 | MIR4712 | microRNA 4712 | non-coding RNA | 55.67480859 | 136.8467414 | 2.45796519 | 1.297464484 | 0.006746515 |
| ENSG00000101438 | SLC32A1 | solute carrier family 32 (GABA vesicular transporter), member 1 | protein-coding gene | 1051.764405 | 2538.810606 | 2.413858649 | 1.271341197 | 0.030432441 |
| ENSG00000225880 | LINC00115 | long intergenic non-protein coding RNA 115 | non-coding RNA | 45.60351944 | 109.3698082 | 2.398275605 | 1.26199746 | 0.013149466 |
| ENSG00000204305 | AGER | advanced glycosylation end product-specific receptor | protein-coding gene | 99.50402354 | 237.6391317 | 2.388236407 | 1.255945654 | 0.004134302 |
| ENSG00000237672 | KRR1P1 | KRR1, small subunit (SSU) processome component, homolog (yeast) pseudogene 1 | pseudogene | 13.82134786 | 33.00679283 | 2.388102316 | 1.255864649 | 0.029311282 |
| ENSG00000075643 | MOCOS | molybdenum cofactor sulfurase | protein-coding gene | 39.69524799 | 94.73260394 | 2.386497345 | 1.254894731 | 0.01555328 |
| ENSG00000231473 | LINC00441 | long intergenic non-protein coding RNA 441 | non-coding RNA | 16.69046182 | 39.66022167 | 2.376220747 | 1.248668867 | 0.024412853 |
| ENSG00000256463 | SALL3 | spalt-like transcription factor 3 | protein-coding gene | 386.8882034 | 917.8256032 | 2.372327704 | 1.246303312 | 0.015381693 |
| ENSG00000174948 | GPR149 | G protein-coupled receptor 149 | protein-coding gene | 39.00092039 | 92.46148576 | 2.370751378 | 1.245344375 | 0.041853308 |
| ENSG00000213721 | HMGN2P30 | high mobility group nucleosomal binding domain 2 pseudogene 30 | pseudogene | 12.29309187 | 29.02481604 | 2.361067202 | 1.239439105 | 0.029633347 |
| ENSG00000127362 | TAS2R3 | taste receptor, type 2, member 3 | protein-coding gene | 21.30255388 | 50.01527249 | 2.34785335 | 1.231342299 | 0.025133509 |
| ENSG00000249835 | VCAN-AS1 | VCAN antisense RNA 1 | non-coding RNA | 23.61000427 | 54.49784722 | 2.308252324 | 1.206800939 | 0.026598043 |
| ENSG00000249437 | NAIP | NLR family, apoptosis inhibitory protein | protein-coding gene | 672.8112856 | 1537.317093 | 2.284915734 | 1.192140961 | 0.011108829 |
| ENSG00000122254 | HS3ST2 | heparan sulfate (glucosamine) 3-O-sulfotransferase 2 | protein-coding gene | 215.6866837 | 490.7076402 | 2.275094742 | 1.185926625 | 0.016308251 |
| ENSG00000139832 | RAB20 | RAB20, member RAS oncogene family | protein-coding gene | 31.16991627 | 70.66128141 | 2.266970524 | 1.180765633 | 0.043737484 |
| ENSG00000246174 | KCTD21-AS1 | KCTD21 antisense RNA 1 | non-coding RNA | 95.2773559 | 214.7840998 | 2.25430374 | 1.172681914 | 0.007353828 |
| ENSG00000239552 | HOXB-AS2 | HOXB cluster antisense RNA 2 | non-coding RNA | 111.1900199 | 250.4568269 | 2.252511756 | 1.171534635 | 0.020223407 |
| ENSG00000187726 | DNAJB13 | DnaJ heat shock protein family (Hsp40) member B13 | protein-coding gene | 16.42207663 | 36.96455723 | 2.25090639 | 1.17050606 | 0.045892198 |
| ENSG00000235436 | DPY19L2P4 | DPY19L2 pseudogene 4 | pseudogene | 121.548024 | 273.5756212 | 2.25076157 | 1.170413236 | 0.006060082 |
| ENSG00000166959 | MS4A8 | membrane-spanning 4-domains, subfamily A, member 8 | protein-coding gene | 8.73138394 | 19.48844634 | 2.231999701 | 1.158336834 | 0.04104481 |
| ENSG00000236871 | LINC00106 | long intergenic non-protein coding RNA 106 | non-coding RNA | 18.07554324 | 40.14354388 | 2.220876205 | 1.151128977 | 0.030185762 |
| ENSG00000158887 | MPZ | myelin protein zero | protein-coding gene | 16.22820369 | 35.85301831 | 2.209302951 | 1.143591262 | 0.025592176 |
| ENSG00000115353 | TACR1 | tachykinin receptor 1 | protein-coding gene | 515.7804942 | 1137.425302 | 2.205250711 | 1.140942683 | 0.043081915 |
| ENSG00000226416 | MRPL23-AS1 | MRPL23 antisense RNA 1 | non-coding RNA | 47.46984832 | 104.2545173 | 2.196226047 | 1.135026552 | 0.02189921 |
| ENSG00000177432 | NAP1L5 | nucleosome assembly protein 1-like 5 | protein-coding gene | 2212.658998 | 4846.468714 | 2.190336929 | 1.13115281 | 0.020456899 |
| ENSG00000204420 | C6orf25 | chromosome 6 open reading frame 25 | protein-coding gene | 31.74315438 | 69.00990331 | 2.174009 | 1.120357913 | 0.045405561 |
| ENSG00000101160 | CTSZ | cathepsin Z | protein-coding gene | 18.42997287 | 39.61603225 | 2.149543709 | 1.104030447 | 0.044344447 |
| ENSG00000139364 | TMEM132B | transmembrane protein 132B | protein-coding gene | 5222.764097 | 11152.03697 | 2.135274878 | 1.094421803 | 0.040971559 |
| ENSG00000239704 | CDRT4 | CMT1A duplicated region transcript 4 | protein-coding gene | 24.23436248 | 51.44189886 | 2.122684222 | 1.085889767 | 0.035660042 |
| ENSG00000137561 | TTPA | tocopherol (alpha) transfer protein | protein-coding gene | 44.33192936 | 93.02175383 | 2.098301499 | 1.06922199 | 0.031484978 |
| ENSG00000247157 | LINC01252 | long intergenic non-protein coding RNA 1252 | non-coding RNA | 26.38033104 | 54.79915384 | 2.077273168 | 1.054690948 | 0.036487897 |
| ENSG00000103184 | SEC14L5 | SEC14-like lipid binding 5 | protein-coding gene | 47.23643101 | 97.03019793 | 2.054139059 | 1.038533851 | 0.029761305 |
| ENSG00000163755 | HPS3 | Hermansky-Pudlak syndrome 3 | protein-coding gene | 598.1834484 | 1212.55809 | 2.02706727 | 1.019393967 | 0.012906439 |
| ENSG00000185522 | LMNTD2 | lamin tail domain containing 2 | protein-coding gene | 36.23468047 | 72.52847522 | 2.001631428 | 1.001176347 | 0.045074614 |
| ENSG00000118557 | PMFBP1 | polyamine modulated factor 1 binding protein 1 | protein-coding gene | 63.27698614 | 122.7478387 | 1.939849639 | 0.955944831 | 0.026474816 |
| ENSG00000182957 | SPATA13 | spermatogenesis associated 13 | protein-coding gene | 3509.871619 | 6739.494713 | 1.920154195 | 0.941222169 | 0.043372451 |
| ENSG00000145020 | AMT | aminomethyltransferase | protein-coding gene | 290.7803205 | 552.4331024 | 1.899829745 | 0.925870136 | 0.029444544 |
| ENSG00000165309 | ARMC3 | armadillo repeat containing 3 | protein-coding gene | 157.6255717 | 299.1329156 | 1.897743572 | 0.924285065 | 0.042068929 |
| ENSG00000253230 | LINC00599 | long intergenic non-protein coding RNA 599 | non-coding RNA | 1497.208506 | 2805.938543 | 1.874113412 | 0.90620826 | 0.045550459 |
| ENSG00000141314 | RHBDL3 | rhomboid, veinlet-like 3 (Drosophila) | protein-coding gene | 2197.742397 | 4114.593332 | 1.872190908 | 0.904727554 | 0.034743087 |
| ENSG00000181085 | MAPK15 | mitogen-activated protein kinase 15 | protein-coding gene | 330.0875967 | 601.4500345 | 1.822092198 | 0.865595961 | 0.044296459 |
| ENSG00000197044 | ZNF441 | zinc finger protein 441 | protein-coding gene | 873.0316742 | 1585.927166 | 1.816574602 | 0.861220614 | 0.040849866 |
| ENSG00000188783 | PRELP | proline/arginine-rich end leucine-rich repeat protein | protein-coding gene | 99.93301536 | 180.5192838 | 1.806402851 | 0.853119668 | 0.04950286 |
| ENSG00000099954 | CECR2 | cat eye syndrome chromosome region, candidate 2 | protein-coding gene | 1455.064577 | 2587.630967 | 1.778361599 | 0.830548701 | 0.036045922 |
| ENSG00000137857 | DUOX1 | dual oxidase 1 | protein-coding gene | 219.8676 | 387.8807525 | 1.76415603 | 0.818978165 | 0.046694892 |
| ENSG00000114805 | PLCH1 | phospholipase C eta 1 | protein-coding gene | 2287.606227 | 4020.843892 | 1.757664342 | 0.813659588 | 0.045065475 |
| ENSG00000159200 | RCAN1 | regulator of calcineurin 1 | protein-coding gene | 7566.869678 | 4543.019553 | 0.600382952 | -0.736045084 | 0.049849017 |
| ENSG00000166342 | NETO1 | neuropilin (NRP) and tolloid (TLL)-like 1 | protein-coding gene | 1154.078131 | 692.8187605 | 0.600322233 | -0.736190996 | 0.046508273 |
| ENSG00000145632 | PLK2 | polo-like kinase 2 | protein-coding gene | 7370.57532 | 4313.420082 | 0.585221627 | -0.772945009 | 0.037206762 |
| ENSG00000182752 | PAPPA | pregnancy-associated plasma protein A, pappalysin 1 | protein-coding gene | 822.0265643 | 478.1998803 | 0.58173288 | -0.781571247 | 0.048078292 |
| ENSG00000109686 | SH3D19 | SH3 domain containing 19 | protein-coding gene | 2033.068675 | 1173.353975 | 0.577134451 | -0.793020641 | 0.043319158 |
| ENSG00000198756 | COLGALT2 | collagen beta(1-O)galactosyltransferase 2 | protein-coding gene | 3870.174024 | 2216.365028 | 0.57267839 | -0.804202931 | 0.037838356 |
| ENSG00000180573 | HIST1H2AC | histone cluster 1, H2ac | protein-coding gene | 1744.525849 | 997.1091259 | 0.571564547 | -0.807011664 | 0.049060942 |
| ENSG00000113070 | HBEGF | heparin-binding EGF-like growth factor | protein-coding gene | 1241.791043 | 707.6233647 | 0.569840932 | -0.811368841 | 0.04312668 |
| ENSG00000177628 | GBA | glucosidase, beta, acid | protein-coding gene | 3010.503668 | 1714.122254 | 0.569380557 | -0.812534866 | 0.034205248 |
| ENSG00000119917 | IFIT3 | interferon-induced protein with tetratricopeptide repeats 3 | protein-coding gene | 513.6745517 | 292.1777893 | 0.568799424 | -0.81400809 | 0.036520884 |
| ENSG00000249158 | PCDHA11 | protocadherin alpha 11 | other | 1007.664451 | 572.7387598 | 0.568382421 | -0.81506616 | 0.046630741 |
| ENSG00000176697 | BDNF | brain-derived neurotrophic factor | protein-coding gene | 412.8502551 | 234.3536762 | 0.567648132 | -0.81693117 | 0.035113148 |
| ENSG00000144959 | NCEH1 | neutral cholesterol ester hydrolase 1 | protein-coding gene | 498.2687866 | 282.2262385 | 0.566413643 | -0.82007208 | 0.038364002 |
| ENSG00000151623 | NR3C2 | nuclear receptor subfamily 3 group C member 2 | protein-coding gene | 586.8349442 | 331.8859693 | 0.565552499 | -0.822267142 | 0.030817591 |
| ENSG00000151491 | EPS8 | epidermal growth factor receptor pathway substrate 8 | protein-coding gene | 5671.113175 | 3156.551358 | 0.556601722 | -0.845282723 | 0.02689567 |
| ENSG00000134996 | OSTF1 | osteoclast stimulating factor 1 | protein-coding gene | 360.391379 | 198.8778022 | 0.5518384 | -0.857682243 | 0.036906215 |
| ENSG00000166750 | SLFN5 | schlafen family member 5 | protein-coding gene | 1971.872151 | 1086.810537 | 0.551156694 | -0.859465558 | 0.042407481 |
| ENSG00000138759 | FRAS1 | Fraser extracellular matrix complex subunit 1 | protein-coding gene | 5735.120606 | 3137.153711 | 0.547007452 | -0.870367607 | 0.042248079 |
| ENSG00000185668 | POU3F1 | POU class 3 homeobox 1 | protein-coding gene | 1562.599065 | 852.3066376 | 0.545441666 | -0.874503183 | 0.024600456 |
| ENSG00000184261 | KCNK12 | potassium channel, two pore domain subfamily K, member 12 | protein-coding gene | 207.7294498 | 112.7301073 | 0.542677542 | -0.881832888 | 0.032014576 |
| ENSG00000185669 | SNAI3 | snail family zinc finger 3 | protein-coding gene | 208.7882742 | 112.1490817 | 0.537142625 | -0.896622883 | 0.045095454 |
| ENSG00000254221 | PCDHGB1 | protocadherin gamma subfamily B, 1 | other | 308.7015413 | 164.658563 | 0.533390803 | -0.906735145 | 0.025199711 |
| ENSG00000149972 | CNTN5 | contactin 5 | protein-coding gene | 2015.050444 | 1073.897729 | 0.532938385 | -0.907959347 | 0.048589764 |
| ENSG00000064601 | CTSA | cathepsin A | protein-coding gene | 3459.146124 | 1838.363951 | 0.531450215 | -0.911993545 | 0.019578855 |
| ENSG00000132329 | RAMP1 | receptor (G protein-coupled) activity modifying protein 1 | protein-coding gene | 247.6350989 | 130.2683812 | 0.526049747 | -0.926728857 | 0.028301656 |
| ENSG00000144452 | ABCA12 | ATP binding cassette subfamily A member 12 | protein-coding gene | 115.0498002 | 60.40910665 | 0.525069201 | -0.929420522 | 0.033424604 |
| ENSG00000158406 | HIST1H4H | histone cluster 1, H4h | protein-coding gene | 130.175625 | 67.97973906 | 0.522215577 | -0.937282604 | 0.047699628 |
| ENSG00000087253 | LPCAT2 | lysophosphatidylcholine acyltransferase 2 | protein-coding gene | 470.9264492 | 242.8901535 | 0.515770889 | -0.955197747 | 0.03225245 |
| ENSG00000254122 | PCDHGB7 | protocadherin gamma subfamily B, 7 | other | 3877.758177 | 1996.607476 | 0.514887052 | -0.957672104 | 0.022767099 |
| ENSG00000183935 | HTR7P1 | 5-hydroxytryptamine (serotonin) receptor 7 pseudogene 1 | pseudogene | 433.0303312 | 221.9085064 | 0.51245488 | -0.964503109 | 0.018650773 |
| ENSG00000156486 | KCNS2 | potassium voltage-gated channel, modifier subfamily S, member 2 | protein-coding gene | 895.9776158 | 457.0014739 | 0.51005903 | -0.971263872 | 0.042953179 |
| ENSG00000231752 | EMBP1 | embigin pseudogene 1 | pseudogene | 68.9709051 | 35.17308704 | 0.509969921 | -0.971515938 | 0.048383756 |
| ENSG00000184492 | FOXD4L1 | forkhead box D4-like 1 | protein-coding gene | 116.3265291 | 58.42264626 | 0.502229773 | -0.99358054 | 0.036794533 |
| ENSG00000196083 | IL1RAP | interleukin 1 receptor accessory protein | protein-coding gene | 997.6500525 | 500.3707533 | 0.501549368 | -0.995536381 | 0.025343181 |
| ENSG00000116745 | RPE65 | retinal pigment epithelium-specific protein 65kDa | protein-coding gene | 1024.936705 | 513.990893 | 0.501485497 | -0.995720116 | 0.040663026 |
| ENSG00000072952 | MRVI1 | murine retrovirus integration site 1 homolog | protein-coding gene | 702.0509587 | 351.2014812 | 0.500250697 | -0.999276824 | 0.013517458 |
| ENSG00000231028 | LINC00271 | long intergenic non-protein coding RNA 271 | non-coding RNA | 76.79407423 | 38.30629594 | 0.498818383 | -1.00341346 | 0.026935911 |
| ENSG00000185567 | AHNAK2 | AHNAK nucleoprotein 2 | protein-coding gene | 6674.460295 | 3299.787676 | 0.494390187 | -1.016277986 | 0.020285393 |
| ENSG00000225206 | MIR137HG | MIR137 host gene | non-coding RNA | 133.3681908 | 65.86500146 | 0.493858401 | -1.017830643 | 0.033544658 |
| ENSG00000182253 | SYNM | synemin | protein-coding gene | 5396.562314 | 2652.059457 | 0.491434973 | -1.024927563 | 0.039926899 |
| ENSG00000163803 | PLB1 | phospholipase B1 | protein-coding gene | 227.6811169 | 111.6538551 | 0.490395763 | -1.02798158 | 0.013043853 |
| ENSG00000075429 | CACNG5 | calcium channel, voltage-dependent, gamma subunit 5 | protein-coding gene | 357.8277173 | 174.5955772 | 0.487931954 | -1.035248129 | 0.030198228 |
| ENSG00000161249 | DMKN | dermokine | protein-coding gene | 79.57335055 | 38.79797256 | 0.487574952 | -1.036304083 | 0.027513663 |
| ENSG00000214960 | ISPD | isoprenoid synthase domain containing | protein-coding gene | 110.2364988 | 53.47866771 | 0.485126689 | -1.043566543 | 0.031001919 |
| ENSG00000197445 | C16orf47 | chromosome 16 open reading frame 47 | other | 54.65465586 | 26.16814666 | 0.478790805 | -1.06253265 | 0.027169354 |
| ENSG00000071282 | LMCD1 | LIM and cysteine-rich domains 1 | protein-coding gene | 490.7783404 | 234.4904336 | 0.477792955 | -1.065542513 | 0.030278948 |
| ENSG00000171488 | LRRC8C | leucine-rich repeat containing 8 family member C | protein-coding gene | 772.8671972 | 368.3634476 | 0.476619332 | -1.069090624 | 0.008320898 |
| ENSG00000113749 | HRH2 | histamine receptor H2 | protein-coding gene | 72.59004594 | 34.57576579 | 0.476315524 | -1.070010525 | 0.033420091 |
| ENSG00000197406 | DIO3 | deiodinase, iodothyronine, type III | protein-coding gene | 380.2283794 | 180.3125512 | 0.474221707 | -1.076366392 | 0.010186479 |
| ENSG00000185742 | C11orf87 | chromosome 11 open reading frame 87 | protein-coding gene | 4265.305901 | 2021.020887 | 0.473827888 | -1.077564981 | 0.007979133 |
| ENSG00000180318 | ALX1 | ALX homeobox 1 | protein-coding gene | 98.97698501 | 46.78003559 | 0.472635488 | -1.081200137 | 0.029343984 |
| ENSG00000183671 | GPR1 | G protein-coupled receptor 1 | protein-coding gene | 566.7295154 | 266.7767607 | 0.47073031 | -1.087027344 | 0.037315655 |
| ENSG00000138207 | RBP4 | retinol binding protein 4, plasma | protein-coding gene | 61.08047711 | 28.74786699 | 0.470655574 | -1.087256415 | 0.018426638 |
| ENSG00000163235 | TGFA | transforming growth factor alpha | protein-coding gene | 131.0778485 | 61.64035179 | 0.47025758 | -1.088476897 | 0.018245225 |
| ENSG00000175040 | CHST2 | carbohydrate (N-acetylglucosamine-6-O) sulfotransferase 2 | protein-coding gene | 3409.593211 | 1599.450535 | 0.469103038 | -1.09202325 | 0.017524468 |
| ENSG00000196353 | CPNE4 | copine IV | protein-coding gene | 1639.187576 | 758.8385426 | 0.462935758 | -1.111116093 | 0.010536611 |
| ENSG00000118508 | RAB32 | RAB32, member RAS oncogene family | protein-coding gene | 705.2863136 | 325.2042278 | 0.461095333 | -1.116863031 | 0.041896152 |
| ENSG00000203724 | C1orf53 | chromosome 1 open reading frame 53 | protein-coding gene | 51.55612747 | 23.69687544 | 0.459632571 | -1.121447058 | 0.035258161 |
| ENSG00000162722 | TRIM58 | tripartite motif containing 58 | protein-coding gene | 219.4291629 | 100.3488026 | 0.457317529 | -1.128731876 | 0.031927842 |
| ENSG00000166592 | RRAD | Ras-related associated with diabetes | protein-coding gene | 41.39254151 | 18.83786908 | 0.455102982 | -1.135735055 | 0.018579649 |
| ENSG00000228203 | RNF144A-AS1 | RNF144A antisense RNA 1 | non-coding RNA | 638.0390663 | 289.2393581 | 0.453325468 | -1.141380881 | 0.00907533 |
| ENSG00000170122 | FOXD4 | forkhead box D4 | protein-coding gene | 112.8998218 | 51.16664927 | 0.453203986 | -1.141767545 | 0.018192005 |
| ENSG00000179344 | HLA-DQB1 | major histocompatibility complex, class II, DQ beta 1 | protein-coding gene | 106.1559096 | 47.98369384 | 0.452011518 | -1.145568559 | 0.044489107 |
| ENSG00000189280 | GJB5 | gap junction protein beta 5 | protein-coding gene | 17.70117223 | 7.9464056 | 0.448919738 | -1.155470565 | 0.049285646 |
| ENSG00000244405 | ETV5 | ets variant 5 | protein-coding gene | 2248.181415 | 990.0855866 | 0.440393991 | -1.183133309 | 0.00514616 |
| ENSG00000160307 | S100B | S100 calcium binding protein B | protein-coding gene | 1956.826915 | 859.7887491 | 0.439379049 | -1.186462016 | 0.004848701 |
| ENSG00000238243 | OR2W3 | olfactory receptor, family 2, subfamily W, member 3 | protein-coding gene | 88.07177221 | 38.31823416 | 0.435079631 | -1.200648618 | 0.042940176 |
| ENSG00000106066 | CPVL | carboxypeptidase, vitellogenic-like | protein-coding gene | 636.7176901 | 274.2404472 | 0.43070964 | -1.215212481 | 0.046393366 |
| ENSG00000183773 | AIFM3 | apoptosis inducing factor, mitochondria associated 3 | protein-coding gene | 81.48398838 | 35.04607436 | 0.43009768 | -1.217263746 | 0.010760765 |
| ENSG00000235847 | LDHAP7 | lactate dehydrogenase A pseudogene 7 | pseudogene | 151.5527146 | 65.00709558 | 0.428940489 | -1.22115059 | 0.014709819 |
| ENSG00000164761 | TNFRSF11B | tumor necrosis factor receptor superfamily member 11b | protein-coding gene | 239.1172592 | 100.0779678 | 0.418530925 | -1.256593867 | 0.043503469 |
| ENSG00000081148 | IMPG2 | interphotoreceptor matrix proteoglycan 2 | protein-coding gene | 151.6962843 | 63.45216937 | 0.418284269 | -1.257444354 | 0.009433782 |
| ENSG00000060140 | STYK1 | serine/threonine/tyrosine kinase 1 | protein-coding gene | 192.0537097 | 80.2867504 | 0.418043216 | -1.258276004 | 0.017990784 |
| ENSG00000187678 | SPRY4 | sprouty RTK signaling antagonist 4 | protein-coding gene | 1944.51286 | 811.7987235 | 0.417481797 | -1.2602148 | 0.045923327 |
| ENSG00000206341 | HLA-H | major histocompatibility complex, class I, H (pseudogene) | pseudogene | 611.4556475 | 252.9436454 | 0.413674559 | -1.27343186 | 0.015695468 |
| ENSG00000131471 | AOC3 | amine oxidase, copper containing 3 | protein-coding gene | 217.8253456 | 89.35519017 | 0.410214844 | -1.285548397 | 0.030345003 |
| ENSG00000156466 | GDF6 | growth differentiation factor 6 | protein-coding gene | 145.3810642 | 58.91981275 | 0.405278453 | -1.303014621 | 0.008055791 |
| ENSG00000081052 | COL4A4 | collagen, type IV, alpha 4 | protein-coding gene | 67.65009055 | 27.12863445 | 0.401014015 | -1.318275436 | 0.007541179 |
| ENSG00000139278 | GLIPR1 | GLI pathogenesis-related 1 | protein-coding gene | 782.7826077 | 312.9929747 | 0.399846613 | -1.322481426 | 0.007195258 |
| ENSG00000183346 | C10orf107 | chromosome 10 open reading frame 107 | protein-coding gene | 84.96038212 | 33.92657367 | 0.399322282 | -1.324374519 | 0.040111808 |
| ENSG00000237892 | KLF7-IT1 | KLF7 intronic transcript 1 | non-coding RNA | 23.92470204 | 9.459320937 | 0.39537884 | -1.338692433 | 0.03321988 |
| ENSG00000235448 | LURAP1L-AS1 | LURAP1L antisense RNA 1 | non-coding RNA | 53.39658844 | 20.99657808 | 0.393219468 | -1.346593346 | 0.007362522 |
| ENSG00000075073 | TACR2 | tachykinin receptor 2 | protein-coding gene | 46.06328973 | 17.8640444 | 0.387815211 | -1.366558706 | 0.007886936 |
| ENSG00000260910 | LINC00565 | long intergenic non-protein coding RNA 565 | non-coding RNA | 85.05190552 | 32.90085 | 0.386832603 | -1.370218702 | 0.003118473 |
| ENSG00000181215 | C4orf50 | chromosome 4 open reading frame 50 | protein-coding gene | 226.4738011 | 86.73857198 | 0.382996053 | -1.38459857 | 0.022577902 |
| ENSG00000246898 | LINC00920 | long intergenic non-protein coding RNA 920 | non-coding RNA | 77.80265738 | 29.73673473 | 0.382207186 | -1.387573192 | 0.033307829 |
| ENSG00000238021 | ARMC4P1 | armadillo repeat containing 4 pseudogene 1 | pseudogene | 48.36002198 | 18.4685328 | 0.3818967 | -1.388745642 | 0.010097942 |
| ENSG00000135046 | ANXA1 | annexin A1 | protein-coding gene | 3617.876821 | 1363.239796 | 0.376806581 | -1.408103932 | 0.024053162 |
| ENSG00000139410 | SDSL | serine dehydratase-like | protein-coding gene | 344.7910071 | 129.7747583 | 0.376386726 | -1.409712345 | 0.015036715 |
| ENSG00000227825 | SLC9A7P1 | solute carrier family 9, subfamily A (NHE7, cation proton antiporter 7), member 7 pseudogene 1 | pseudogene | 14.45773756 | 5.420357031 | 0.374910459 | -1.41538202 | 0.029070032 |
| ENSG00000182057 | OGFRP1 | opioid growth factor receptor pseudogene 1 | pseudogene | 14.72387606 | 5.497166132 | 0.373350476 | -1.421397525 | 0.03736774 |
| ENSG00000164619 | BMPER | BMP binding endothelial regulator | protein-coding gene | 387.575725 | 144.1556026 | 0.371941774 | -1.426851305 | 0.044770519 |
| ENSG00000112812 | PRSS16 | protease, serine 16 | protein-coding gene | 115.4458424 | 42.32724727 | 0.366641591 | -1.447557646 | 0.001492773 |
| ENSG00000188039 | NWD1 | NACHT and WD repeat domain containing 1 | protein-coding gene | 235.8799844 | 85.72504441 | 0.363426531 | -1.460264352 | 0.041544258 |
| ENSG00000158748 | HTR6 | 5-hydroxytryptamine (serotonin) receptor 6, G protein-coupled | protein-coding gene | 24.05946451 | 8.68986274 | 0.361182716 | -1.469199239 | 0.013624784 |
| ENSG00000242349 | NPPA-AS1 | NPPA antisense RNA 1 | non-coding RNA | 24.3973707 | 8.796261073 | 0.360541354 | -1.471763348 | 0.008123616 |
| ENSG00000104361 | NIPAL2 | NIPA-like domain containing 2 | protein-coding gene | 417.716048 | 150.4664735 | 0.360212336 | -1.473080505 | 0.041933701 |
| ENSG00000081923 | ATP8B1 | ATPase, aminophospholipid transporter, class I, type 8B, member 1 | protein-coding gene | 72.89431944 | 25.9069043 | 0.35540361 | -1.492469758 | 0.029218353 |
| ENSG00000177459 | ERICH5 | glutamate rich 5 | protein-coding gene | 100.1347496 | 35.56312336 | 0.355152667 | -1.493488777 | 0.016115805 |
| ENSG00000126861 | OMG | oligodendrocyte myelin glycoprotein | protein-coding gene | 613.0083817 | 215.2619086 | 0.35115655 | -1.509813748 | 0.0011905 |
| ENSG00000144115 | THNSL2 | threonine synthase-like 2 | protein-coding gene | 763.0640544 | 267.2822894 | 0.350275036 | -1.513439922 | 0.00540395 |
| ENSG00000197355 | UAP1L1 | UDP-N-acetylglucosamine pyrophosphorylase 1 like 1 | protein-coding gene | 954.8628586 | 331.0179697 | 0.346665457 | -1.528384005 | 0.001131664 |
| ENSG00000164344 | KLKB1 | kallikrein B1 | protein-coding gene | 23.4630209 | 8.102005988 | 0.345309584 | -1.534037718 | 0.013252289 |
| ENSG00000188993 | LRRC66 | leucine rich repeat containing 66 | protein-coding gene | 82.15148204 | 28.33095575 | 0.344862382 | -1.53590733 | 0.001717247 |
| ENSG00000138772 | ANXA3 | annexin A3 | protein-coding gene | 132.2553538 | 45.43548979 | 0.343543671 | -1.541434589 | 0.034852495 |
| ENSG00000128564 | VGF | VGF nerve growth factor inducible | protein-coding gene | 1107.710188 | 380.1864169 | 0.343218308 | -1.542801582 | 0.000204313 |
| ENSG00000170345 | FOS | FBJ murine osteosarcoma viral oncogene homolog | protein-coding gene | 902.2141594 | 308.0266695 | 0.341411921 | -1.550414661 | 0.029469826 |
| ENSG00000100285 | NEFH | neurofilament, heavy polypeptide | protein-coding gene | 1090.203562 | 366.04294 | 0.335756507 | -1.574512735 | 0.026393968 |
| ENSG00000164106 | SCRG1 | stimulator of chondrogenesis 1 | protein-coding gene | 1403.500244 | 469.4670566 | 0.33449731 | -1.579933485 | 0.047541192 |
| ENSG00000179256 | SMCO3 | single-pass membrane protein with coiled-coil domains 3 | protein-coding gene | 18.33525782 | 6.000467937 | 0.327263898 | -1.611473636 | 0.026878628 |
| ENSG00000139549 | DHH | desert hedgehog | protein-coding gene | 11.90127421 | 3.865451717 | 0.324793098 | -1.622407121 | 0.031338707 |
| ENSG00000255277 | ABCC6P2 | ATP binding cassette subfamily C member 6 pseudogene 2 | pseudogene | 23.07935355 | 7.464299607 | 0.32341892 | -1.628524015 | 0.039875015 |
| ENSG00000104689 | TNFRSF10A | tumor necrosis factor receptor superfamily member 10a | protein-coding gene | 24.44266031 | 7.901138756 | 0.323251997 | -1.629268812 | 0.029940834 |
| ENSG00000092068 | SLC7A8 | solute carrier family 7 (amino acid transporter light chain, L system), member 8 | protein-coding gene | 1409.400717 | 448.4896773 | 0.318213033 | -1.651935169 | 0.01644057 |
| ENSG00000118785 | SPP1 | secreted phosphoprotein 1 | protein-coding gene | 1955.722104 | 619.5140756 | 0.316769992 | -1.65849242 | 0.004696785 |
| ENSG00000042062 | FAM65C | family with sequence similarity 65 member C | protein-coding gene | 270.5514708 | 85.10291826 | 0.314553523 | -1.668622575 | 0.048785026 |
| ENSG00000260799 | KRT8P50 | keratin 8 pseudogene 50 | pseudogene | 9.605507998 | 3.002979386 | 0.312630981 | -1.677467343 | 0.038912684 |
| ENSG00000172965 | MIR4435-2HG | MIR4435-2 host gene | non-coding RNA | 423.9911733 | 131.6692364 | 0.310547117 | -1.687115922 | 0.031789216 |
| ENSG00000115956 | PLEK | pleckstrin | protein-coding gene | 12.37291952 | 3.782843224 | 0.305735701 | -1.709643068 | 0.02290163 |
| ENSG00000268758 | ADGRE4P | adhesion G protein-coupled receptor E4, pseudogene | pseudogene | 10.10550787 | 3.055782998 | 0.30238787 | -1.725527828 | 0.033974941 |
| ENSG00000269067 | ZNF728 | zinc finger protein 728 | protein-coding gene | 160.0355716 | 48.09215019 | 0.300509129 | -1.734519278 | 0.00379116 |
| ENSG00000124249 | KCNK15 | potassium channel, two pore domain subfamily K, member 15 | protein-coding gene | 8.460605355 | 2.530906928 | 0.29914017 | -1.741106437 | 0.038185879 |
| ENSG00000237194 | SNAI1P1 | snail family zinc finger 1 pseudogene 1 | pseudogene | 13.63604612 | 4.035376303 | 0.295934486 | -1.756650267 | 0.028076367 |
| ENSG00000214922 | HLA-F-AS1 | HLA-F antisense RNA 1 | non-coding RNA | 13.23152925 | 3.901289489 | 0.294847966 | -1.761956852 | 0.027147308 |
| ENSG00000250331 | LINC01340 | long intergenic non-protein coding RNA 1340 | non-coding RNA | 19.16290982 | 5.643515082 | 0.29450199 | -1.763650711 | 0.007705855 |
| ENSG00000113600 | C9 | complement component 9 | protein-coding gene | 9.941830373 | 2.920275237 | 0.293736176 | -1.767407138 | 0.033966724 |
| ENSG00000157765 | SLC34A2 | solute carrier family 34 (type II sodium/phosphate cotransporter), member 2 | protein-coding gene | 87.30599499 | 25.10668964 | 0.287571199 | -1.798008902 | 0.023022455 |
| ENSG00000229585 | RPL21P44 | ribosomal protein L21 pseudogene 44 | pseudogene | 12.02373316 | 3.434697321 | 0.285659809 | -1.807630024 | 0.03965468 |
| ENSG00000154065 | ANKRD29 | ankyrin repeat domain 29 | protein-coding gene | 407.1410961 | 116.066735 | 0.285077424 | -1.810574301 | 0.032432102 |
| ENSG00000259378 | DCAF13P3 | DDB1 and CUL4 associated factor 13 pseudogene 3 | pseudogene | 26.64009436 | 7.560427965 | 0.283798843 | -1.817059386 | 0.003093099 |
| ENSG00000091664 | SLC17A6 | solute carrier family 17 (vesicular glutamate transporter), member 6 | protein-coding gene | 7836.69017 | 2208.499828 | 0.281815381 | -1.82717774 | 0.007710461 |
| ENSG00000232629 | HLA-DQB2 | major histocompatibility complex, class II, DQ beta 2 | protein-coding gene | 26.36645867 | 7.290369716 | 0.276501665 | -1.854639928 | 0.029157235 |
| ENSG00000144671 | SLC22A14 | solute carrier family 22, member 14 | protein-coding gene | 19.94772532 | 5.514336311 | 0.276439354 | -1.85496508 | 0.022346156 |
| ENSG00000141934 | PPAP2C | phospholipid phosphatase 2 | protein-coding gene | 69.17356313 | 18.97167188 | 0.274261886 | -1.86637395 | 0.000694695 |
| ENSG00000110975 | SYT10 | synaptotagmin 10 | protein-coding gene | 405.4373625 | 110.0603048 | 0.271460687 | -1.881184815 | 0.036490593 |
| ENSG00000222041 | LINC00152 | long intergenic non-protein coding RNA 152 | non-coding RNA | 204.6684097 | 55.52532592 | 0.27129407 | -1.88207058 | 0.028728555 |
| ENSG00000134007 | ADAM20 | ADAM metallopeptidase domain 20 | protein-coding gene | 20.45884023 | 5.476658285 | 0.267691532 | -1.901356592 | 0.007435009 |
| ENSG00000100311 | PDGFB | platelet-derived growth factor beta polypeptide | protein-coding gene | 687.0097382 | 182.9183322 | 0.266252896 | -1.909130879 | 0.037319385 |
| ENSG00000112499 | SLC22A2 | solute carrier family 22 (organic cation transporter), member 2 | protein-coding gene | 9.61265029 | 2.553768959 | 0.26566752 | -1.91230624 | 0.036701176 |
| ENSG00000171345 | KRT19 | keratin 19, type I | protein-coding gene | 23.06707524 | 6.085240141 | 0.263806316 | -1.922448989 | 0.016421044 |
| ENSG00000137801 | THBS1 | thrombospondin 1 | protein-coding gene | 6591.95897 | 1719.053494 | 0.260780369 | -1.939092824 | 0.042925447 |
| ENSG00000147883 | CDKN2B | cyclin-dependent kinase inhibitor 2B (p15, inhibits CDK4) | protein-coding gene | 526.1058416 | 135.8829697 | 0.258280671 | -1.952988416 | 0.037100964 |
| ENSG00000006128 | TAC1 | tachykinin, precursor 1 | protein-coding gene | 9908.007864 | 2494.84013 | 0.251800378 | -1.989647643 | 0.002477374 |
| ENSG00000063515 | GSC2 | goosecoid homeobox 2 | protein-coding gene | 8.486052985 | 2.102406971 | 0.247748509 | -2.013051722 | 0.028751214 |
| ENSG00000102760 | RGCC | regulator of cell cycle | protein-coding gene | 291.6418315 | 72.20841801 | 0.247592801 | -2.013958727 | 0.025367345 |
| ENSG00000188501 | LCTL | lactase like | protein-coding gene | 142.4357523 | 34.95457568 | 0.245405912 | -2.02675809 | 0.039131427 |
| ENSG00000169618 | PROKR1 | prokineticin receptor 1 | protein-coding gene | 141.5696542 | 34.59683846 | 0.244380328 | -2.032799941 | 0.038849268 |
| ENSG00000183571 | PGPEP1L | pyroglutamyl-peptidase I-like | protein-coding gene | 8.189868391 | 1.976815803 | 0.24137333 | -2.05066182 | 0.034550197 |
| ENSG00000197980 | LEKR1 | leucine, glutamate and lysine rich 1 | protein-coding gene | 9.660078146 | 2.307748877 | 0.238895467 | -2.065548618 | 0.024052263 |
| ENSG00000259207 | ITGB3 | integrin beta 3 | protein-coding gene | 339.7858317 | 80.7969046 | 0.237787739 | -2.072253768 | 0.011571393 |
| ENSG00000133105 | RXFP2 | relaxin/insulin-like family peptide receptor 2 | protein-coding gene | 13.073358 | 3.018615384 | 0.230898242 | -2.114670904 | 0.02581266 |
| ENSG00000168685 | IL7R | interleukin 7 receptor | protein-coding gene | 12.87317563 | 2.919161508 | 0.226763123 | -2.140742052 | 0.037686797 |
| ENSG00000234883 | MIR155HG | MIR155 host gene | non-coding RNA | 8.229913889 | 1.844092078 | 0.224071856 | -2.157966642 | 0.045965778 |
| ENSG00000120156 | TEK | TEK tyrosine kinase, endothelial | protein-coding gene | 754.6999363 | 167.677725 | 0.222178003 | -2.170212104 | 0.042064223 |
| ENSG00000137757 | CASP5 | caspase 5 | protein-coding gene | 5.918278079 | 1.307647724 | 0.220950707 | -2.17820355 | 0.034299117 |
| ENSG00000232177 | MTND4P24 | mitochondrially encoded NADH:ubiquinone oxidoreductase core subunit 4 pseudogene 24 | pseudogene | 68.07952596 | 14.89031008 | 0.218719356 | -2.192847194 | 0.021004213 |
| ENSG00000255222 | SETP17 | SET pseudogene 17 | pseudogene | 7.201500957 | 1.566846169 | 0.217572167 | -2.200434084 | 0.032273306 |
| ENSG00000237512 | UNC5B-AS1 | UNC5B antisense RNA 1 | non-coding RNA | 12.10290058 | 2.608319918 | 0.215511968 | -2.214160108 | 0.005719645 |
| ENSG00000118523 | CTGF | connective tissue growth factor | protein-coding gene | 7619.724875 | 1640.699927 | 0.215322725 | -2.215427504 | 0.004610977 |
| ENSG00000180353 | HCLS1 | hematopoietic cell-specific Lyn substrate 1 | protein-coding gene | 16.8558744 | 3.574424698 | 0.2120581 | -2.237468505 | 0.038839102 |
| ENSG00000236090 | LDHAP3 | lactate dehydrogenase A pseudogene 3 | pseudogene | 64.28398496 | 13.49022532 | 0.209853595 | -2.25254492 | 0.000358855 |
| ENSG00000178222 | RNF212 | ring finger protein 212 | protein-coding gene | 112.5114775 | 23.31149447 | 0.207192146 | -2.270958779 | 0.0167786 |
| ENSG00000143248 | RGS5 | regulator of G-protein signaling 5 | protein-coding gene | 539.042985 | 110.8348923 | 0.2056142 | -2.281988191 | 0.01172852 |
| ENSG00000163661 | PTX3 | pentraxin 3 | protein-coding gene | 5040.195479 | 1035.371274 | 0.205422841 | -2.283331491 | 0.047671926 |
| ENSG00000226278 | PSPHP1 | phosphoserine phosphatase pseudogene 1 | pseudogene | 200.2678637 | 40.10248172 | 0.200244218 | -2.320167511 | 0.040656873 |
| ENSG00000167157 | PRRX2 | paired related homeobox 2 | protein-coding gene | 114.2413685 | 22.2644839 | 0.194889856 | -2.359269092 | 0.030346863 |
| ENSG00000239605 | C2orf61 | chromosome 2 open reading frame 61 | protein-coding gene | 7.542472102 | 1.458962 | 0.193432867 | -2.370095148 | 0.015471207 |
| ENSG00000235884 | LINC00941 | long intergenic non-protein coding RNA 941 | non-coding RNA | 7.054914483 | 1.355085885 | 0.192076869 | -2.380244303 | 0.04141072 |
| ENSG00000223489 | NEFHP1 | neurofilament, heavy polypeptide pseudogene 1 | pseudogene | 14.76340234 | 2.819570362 | 0.190983778 | -2.388477989 | 0.003207999 |
| ENSG00000187758 | ADH1A | alcohol dehydrogenase 1A (class I), alpha polypeptide | protein-coding gene | 14.06499596 | 2.683831283 | 0.190816357 | -2.389743252 | 0.041577708 |
| ENSG00000148702 | HABP2 | hyaluronan binding protein 2 | protein-coding gene | 19.67066349 | 3.632935831 | 0.184688017 | -2.436837832 | 0.000278269 |
| ENSG00000171227 | TMEM37 | transmembrane protein 37 | protein-coding gene | 44.86469261 | 8.084558894 | 0.180198691 | -2.472339561 | 0.009763147 |
| ENSG00000130176 | CNN1 | calponin 1, basic, smooth muscle | protein-coding gene | 352.1815394 | 61.99826078 | 0.176040632 | -2.506019638 | 0.01903944 |
| ENSG00000184459 | BPIFC | BPI fold containing family C | protein-coding gene | 9.408578781 | 1.642865767 | 0.174613595 | -2.517762205 | 0.008810324 |
| ENSG00000230313 | HCG24 | HLA complex group 24 (non-protein coding) | non-coding RNA | 15.45139629 | 2.686009824 | 0.173836058 | -2.524200729 | 0.049168726 |
| ENSG00000169291 | SHE | Src homology 2 domain containing E | protein-coding gene | 43.58391487 | 7.409744559 | 0.170010991 | -2.556300077 | 0.011295696 |
| ENSG00000249231 | CASC16 | cancer susceptibility candidate 16 (non-protein coding) | non-coding RNA | 4.908385089 | 0.816414072 | 0.166330485 | -2.587875486 | 0.035414816 |
| ENSG00000152936 | LMNTD1 | lamin tail domain containing 1 | protein-coding gene | 22.92945722 | 3.799083681 | 0.165685722 | -2.593478809 | 0.004182218 |
| ENSG00000251450 | RASGRF2-AS1 | RASGRF2 antisense RNA 1 | non-coding RNA | 8.484319464 | 1.367413944 | 0.161169549 | -2.633348902 | 0.013995857 |
| ENSG00000149418 | ST14 | suppression of tumorigenicity 14 | protein-coding gene | 5.388387367 | 0.8432644 | 0.156496618 | -2.675796615 | 0.031457288 |
| ENSG00000232560 | LINC01549 | long intergenic non-protein coding RNA 1549 | non-coding RNA | 12.31446061 | 1.858151246 | 0.15089181 | -2.728413596 | 0.002952257 |
| ENSG00000152580 | IGSF10 | immunoglobulin superfamily member 10 | protein-coding gene | 1145.312459 | 169.1696245 | 0.147706089 | -2.75919879 | 0.047429995 |
| ENSG00000188095 | MESP2 | mesoderm posterior bHLH transcription factor 2 | protein-coding gene | 6.783773511 | 0.918666618 | 0.135421181 | -2.884474693 | 0.049022417 |
| ENSG00000163239 | TDRD10 | tudor domain containing 10 | protein-coding gene | 21.41486565 | 2.796522145 | 0.130587891 | -2.936906966 | 0.00021248 |
| ENSG00000188883 | KLRG2 | killer cell lectin-like receptor subfamily G, member 2 | protein-coding gene | 4.599855359 | 0.574678959 | 0.124934137 | -3.000760363 | 0.039056618 |
| ENSG00000223949 | ROR1-AS1 | ROR1 antisense RNA 1 | non-coding RNA | 34.48493911 | 3.670441731 | 0.10643608 | -3.231940815 | 0.04164237 |
| ENSG00000188133 | TMEM215 | transmembrane protein 215 | protein-coding gene | 79.27850279 | 8.04790112 | 0.101514292 | -3.300245232 | 0.02602191 |
| ENSG00000081051 | AFP | alpha-fetoprotein | protein-coding gene | 22.69948113 | 2.103134639 | 0.092651221 | -3.432046203 | 0.039896094 |
| ENSG00000235529 | AGAP1-IT1 | AGAP1 intronic transcript 1 | non-coding RNA | 4.471170576 | 0.37597326 | 0.084088328 | -3.571950623 | 0.027094224 |
| ENSG00000228078 | HLA-U | major histocompatibility complex, class I, U (pseudogene) | pseudogene | 4.884770384 | 0.37597326 | 0.076968461 | -3.699588785 | 0.011980041 |
| ENSG00000139151 | PLCZ1 | phospholipase C zeta 1 | protein-coding gene | 4.990401293 | 0.349122932 | 0.069958889 | -3.837348802 | 0.022982572 |
| ENSG00000189068 | VSTM1 | V-set and transmembrane domain containing 1 | protein-coding gene | 4.244558008 | 0.191559653 | 0.045130648 | -4.469748699 | 0.013541684 |
| ENSG00000213130 | EEF1DP5 | eukaryotic translation elongation factor 1 delta pseudogene 5 | pseudogene | 16.28093187 | 0.537558084 | 0.033017648 | -4.920618819 | 0.026534531 |
| ENSG00000238245 | MYO5BP2 | myosin VB pseudogene 2 | pseudogene | 11.97172263 | 0.351133705 | 0.029330257 | -5.091466464 | 0.012183643 |
| ENSG00000227582 | ADGRF5P1 | adhesion G protein-coupled receptor F5 pseudogene 1 | pseudogene | 70.89139466 | 2.042797778 | 0.028815878 | -5.116992213 | 0.01017281 |
| ENSG00000113263 | ITK | IL2-inducible T-cell kinase | protein-coding gene | 25.87311275 | 0.730231466 | 0.028223564 | -5.146955985 | 0.042029314 |
| ENSG00000205184 | SLC10A5P1 | SLC10A5 pseudogene 1 | pseudogene | 6.344271402 | 0.159574052 | 0.025152463 | -5.31315651 | 0.024902339 |
| ENSG00000230838 | AC093850.2 | long intergenic non-protein coding RNA 1614 | non-coding RNA | 40.92751456 | 0.755968065 | 0.018470901 | -5.758601957 | 0.03216057 |
| ENSG00000197416 | FABP12 | fatty acid binding protein 12 | protein-coding gene | 19.70587237 | 0.349122932 | 0.017716695 | -5.818746683 | 0.009123694 |
| ENSG00000251521 | IMPA1P | inositol(myo)-1(or 4)-monophosphatase 1 pseudogene | pseudogene | 110.3172462 | 1.646887312 | 0.014928648 | -6.065772696 | 0.00279095 |

**Supplementary Table S6.** Neuron-related genes and corresponding splicing isoforms codes.

| **Ensembl Gene ID** | **Associated Gene Name** | **Description** | **Band** |
| --- | --- | --- | --- |
| ENSG00000022355 | GABRA1 | gamma-aminobutyric acid (GABA) A receptor, alpha 1 [Source:HGNC Symbol;Acc:HGNC:4075] | q34 |
| ENSG00000067715 | SYT1 | synaptotagmin I [Source:HGNC Symbol;Acc:HGNC:11509] | q21.2 |
| ENSG00000081189 | MEF2C | myocyte enhancer factor 2C [Source:HGNC Symbol;Acc:HGNC:6996] | q14.3 |
| ENSG00000091664 | SLC17A6 | solute carrier family 17 (vesicular glutamate transporter), member 6 [Source:HGNC Symbol;Acc:HGNC:16703] | p14.3 |
| ENSG00000101958 | GLRA2 | glycine receptor, alpha 2 [Source:HGNC Symbol;Acc:HGNC:4327] | p22.2 |
| ENSG00000104327 | CALB1 | calbindin 1, 28kDa [Source:HGNC Symbol;Acc:HGNC:1434] | q21.3 |
| ENSG00000104435 | STMN2 | stathmin 2 [Source:HGNC Symbol;Acc:HGNC:10577] | q21.13 |
| ENSG00000113327 | GABRG2 | gamma-aminobutyric acid (GABA) A receptor, gamma 2 [Source:HGNC Symbol;Acc:HGNC:4087] | q34 |
| ENSG00000119042 | SATB2 | SATB homeobox 2 [Source:HGNC Symbol;Acc:HGNC:21637] | q33.1 |
| ENSG00000119125 | GDA | guanine deaminase [Source:HGNC Symbol;Acc:HGNC:4212] | q21.13 |
| ENSG00000122254 | HS3ST2 | heparan sulfate (glucosamine) 3-O-sulfotransferase 2 [Source:HGNC Symbol;Acc:HGNC:5195] | p12.2 |
| ENSG00000124140 | SLC12A5 | solute carrier family 12 (potassium/chloride transporter), member 5 [Source:HGNC Symbol;Acc:HGNC:13818] | q13.12 |
| ENSG00000125851 | PCSK2 | proprotein convertase subtilisin/kexin type 2 [Source:HGNC Symbol;Acc:HGNC:8744] | p12.1 |
| ENSG00000132639 | SNAP25 | synaptosomal-associated protein, 25kDa [Source:HGNC Symbol;Acc:HGNC:11132] | p12.2 |
| ENSG00000133636 | NTS | neurotensin [Source:HGNC Symbol;Acc:HGNC:8038] | q21.31 |
| ENSG00000135333 | EPHA7 | EPH receptor A7 [Source:HGNC Symbol;Acc:HGNC:3390] | q16.1 |
| ENSG00000136999 | NOV | nephroblastoma overexpressed [Source:HGNC Symbol;Acc:HGNC:7885] | q24.12 |
| ENSG00000144355 | DLX1 | distal-less homeobox 1 [Source:HGNC Symbol;Acc:HGNC:2914] | q31.1 |
| ENSG00000145934 | TENM2 | teneurin transmembrane protein 2 [Source:HGNC Symbol;Acc:HGNC:29943] | q34 |
| ENSG00000146469 | VIP | vasoactive intestinal peptide [Source:HGNC Symbol;Acc:HGNC:12693] | q25.2 |
| ENSG00000147246 | HTR2C | 5-hydroxytryptamine (serotonin) receptor 2C, G protein-coupled [Source:HGNC Symbol;Acc:HGNC:5295] | q23 |
| ENSG00000147571 | CRH | corticotropin releasing hormone [Source:HGNC Symbol;Acc:HGNC:2355] | q13.1 |
| ENSG00000147676 | MAL2 | mal, T-cell differentiation protein 2 (gene/pseudogene) [Source:HGNC Symbol;Acc:HGNC:13634] | q24.12 |
| ENSG00000152784 | PRDM8 | PR domain containing 8 [Source:HGNC Symbol;Acc:HGNC:13993] | q21.21 |
| ENSG00000155926 | SLA | Src-like-adaptor [Source:HGNC Symbol;Acc:HGNC:10902] | q24.22 |
| ENSG00000164600 | NEUROD6 | neuronal differentiation 6 [Source:HGNC Symbol;Acc:HGNC:13804] | p14.3 |
| ENSG00000165434 | PGM2L1 | phosphoglucomutase 2-like 1 [Source:HGNC Symbol;Acc:HGNC:20898] | q13.4 |
| ENSG00000171951 | SCG2 | secretogranin II [Source:HGNC Symbol;Acc:HGNC:10575] | q36.1 |
| ENSG00000174576 | NPAS4 | neuronal PAS domain protein 4 [Source:HGNC Symbol;Acc:HGNC:18983] | q13.2 |
| ENSG00000180616 | SSTR2 | somatostatin receptor 2 [Source:HGNC Symbol;Acc:HGNC:11331] | q25.1 |
| ENSG00000181656 | GPR88 | G protein-coupled receptor 88 [Source:HGNC Symbol;Acc:HGNC:4539] | p21.2 |
| ENSG00000182836 | PLCXD3 | phosphatidylinositol-specific phospholipase C, X domain containing 3 [Source:HGNC Symbol;Acc:HGNC:31822] | p13.1 |
| ENSG00000186297 | GABRA5 | gamma-aminobutyric acid (GABA) A receptor, alpha 5 [Source:HGNC Symbol;Acc:HGNC:4079] | q12 |
| ENSG00000186487 | MYT1L | myelin transcription factor 1-like [Source:HGNC Symbol;Acc:HGNC:7623] | p25.3 |

**Supplementary Table S7.** DEG genes (fold-change ≥ 2, *P*-value < 0.05) found in iPSC-derived neurons from AN compared to unaffected controls. The *TACR1* gene expression is highlighted in grey.

| **Ensembl ID** | **Symbol** | **Description** | **Expression in control** | **Expression in AN** | **Fold-Change** | **Log2 (Fold-Change)** | ***P*-value** |
| --- | --- | --- | --- | --- | --- | --- | --- |
| ENSG00000185290 | NUPR1L | nuclear protein, transcriptional regulator, 1-like | 9.833845409 | 0 | 0 | -inf | 0.006376876 |
| ENSG00000205488 | CALML3-AS1 | CALML3 antisense RNA | 2.977907309 | 0 | 0 | -inf | 0.020921536 |
| ENSG00000235315 | RPL23AP69 | ribosomal protein L23a pseudogene 69 | 3.586494966 | 0 | 0 | -inf | 0.010147736 |
| ENSG00000238298 |  |  | 2.978980595 | 0 | 0 | -inf | 0.026348986 |
| ENSG00000242613 | RP11-174O3.4 |  | 6.693144478 | 0 | 0 | -inf | 0.017020024 |
| ENSG00000252982 | RN7SKP234 | RNA, 7SK small nuclear pseudogene 234 | 2.644234917 | 0 | 0 | -inf | 0.030724357 |
| ENSG00000266010 | GATA6-AS1 | GATA6 antisense RNA 1 (head to head) | 9.672935374 | 0 | 0 | -inf | 0.042373015 |
| ENSG00000260409 | RP11-403B2.7 |  | 30.24548953 | 0.189548881 | 0.006267013 | -7.318006263 | 0.003677783 |
| ENSG00000249780 | RP11-352E6.2 |  | 60.12390041 | 0.460145093 | 0.007653281 | -7.029705949 | 0.003511925 |
| ENSG00000240661 | RP11-174O3.3 |  | 33.26027299 | 0.273720714 | 0.008229659 | -6.924951583 | 0.002783834 |
| ENSG00000235130 |  |  | 21.8739851 | 0.186424379 | 0.008522653 | -6.874481733 | 0.015074356 |
| ENSG00000247765 | RP11-32B5.7 |  | 27.80904939 | 0.349122932 | 0.012554292 | -6.315675494 | 0.007127375 |
| ENSG00000251521 | IMPA1P | inositol(myo)-1(or 4)-monophosphatase 1 pseudogene | 110.3172462 | 1.646887312 | 0.014928648 | -6.065772696 | 0.00279095 |
| ENSG00000197416 | FABP12 | fatty acid binding protein 12 | 19.70587237 | 0.349122932 | 0.017716695 | -5.818746683 | 0.009123694 |
| ENSG00000230838 | LINC01614 | long intergenic non-protein coding RNA 1614 | 40.92751456 | 0.755968065 | 0.018470901 | -5.758601957 | 0.03216057 |
| ENSG00000205184 | SLC10A5P1 | SLC10A5 pseudogene 1 | 6.344271402 | 0.159574052 | 0.025152463 | -5.31315651 | 0.024902339 |
| ENSG00000113263 | ITK | IL2-inducible T-cell kinase | 25.87311275 | 0.730231466 | 0.028223564 | -5.146955985 | 0.042029314 |
| ENSG00000227582 | ADGRF5P1 | adhesion G protein-coupled receptor F5 pseudogene 1 | 70.89139466 | 2.042797778 | 0.028815878 | -5.116992213 | 0.01017281 |
| ENSG00000238245 | MYO5BP2 | myosin VB pseudogene 2 | 11.97172263 | 0.351133705 | 0.029330257 | -5.091466464 | 0.012183643 |
| ENSG00000213130 | EEF1DP5 | eukaryotic translation elongation factor 1 delta pseudogene 5 | 16.28093187 | 0.537558084 | 0.033017648 | -4.920618819 | 0.026534531 |
| ENSG00000189068 | VSTM1 | V-set and transmembrane domain containing | 4.244558008 | 0.191559653 | 0.045130648 | -4.469748699 | 0.013541684 |
| ENSG00000227496 | RP11-145A3.1 |  | 12.38934356 | 0.574678959 | 0.04638494 | -4.43019971 | 0.003112029 |
| ENSG00000261573 | RP11-553K8.5 |  | 3.647447406 | 0.189548881 | 0.051967543 | -4.266245333 | 0.035101406 |
| ENSG00000204894 | RP11-208G20.2 |  | 26.99190732 | 1.58604061 | 0.058759857 | -4.089025308 | 0.018663971 |
| ENSG00000257156 | RP11-13A1.3 |  | 3.18335671 | 0.189548881 | 0.059543714 | -4.069906984 | 0.044252725 |
| ENSG00000237531 | RP11-309M23.1 |  | 5.663145809 | 0.381108534 | 0.06729626 | -3.893329857 | 0.048444664 |
| ENSG00000139151 | PLCZ1 | phospholipase C, zeta 1 [Source:HGNC Symbol;Acc:HGNC:19218] | 4.990401293 | 0.349122932 | 0.069958889 | -3.837348802 | 0.022982572 |
| ENSG00000228078 | HLA-U | major histocompatibility complex, class I, U (pseudogene) | 4.884770384 | 0.37597326 | 0.076968461 | -3.699588785 | 0.011980041 |
| ENSG00000232694 |  |  | 6.665294052 | 0.542693358 | 0.081420768 | -3.618459367 | 0.033327777 |
| ENSG00000227116 | RP3-471C18.1 |  | 6.122241145 | 0.508696984 | 0.083089995 | -3.589181425 | 0.011566553 |
| ENSG00000235529 | AGAP1-IT1 | AGAP1 intronic transcript 1 | 4.471170576 | 0.37597326 | 0.084088328 | -3.571950623 | 0.027094224 |
| ENSG00000234663 | AC104820.2 |  | 3.811579578 | 0.351133705 | 0.092122884 | -3.440296607 | 0.040252796 |
| ENSG00000081051 | AFP | alpha-fetoprotein | 22.69948113 | 2.103134639 | 0.092651221 | -3.432046203 | 0.039896094 |
| ENSG00000234665 | RP11-262H14.3 |  | 40.34466562 | 3.996768559 | 0.099065601 | -3.335471995 | 0.008778453 |
| ENSG00000188133 | TMEM215 | transmembrane protein 215 | 79.27850279 | 8.04790112 | 0.101514292 | -3.300245232 | 0.02602191 |
| ENSG00000223949 | ROR1-AS1 | ROR1 antisense RNA 1 | 34.48493911 | 3.670441731 | 0.10643608 | -3.231940815 | 0.04164237 |
| ENSG00000222032 | AC112721.2 |  | 51.03862965 | 6.180531684 | 0.121095173 | -3.045786737 | 0.044735099 |
| ENSG00000267642 | RP11-258B16.1 |  | 5.73210826 | 0.695121363 | 0.12126801 | -3.043729068 | 0.022110263 |
| ENSG00000222022 | AC112721.1 |  | 27.80980924 | 3.434230131 | 0.123489885 | -3.017535224 | 0.048814847 |
| ENSG00000188883 | KLRG2 | killer cell lectin-like receptor subfamily G, member 2 | 4.599855359 | 0.574678959 | 0.124934137 | -3.000760363 | 0.039056618 |
| ENSG00000231429 | RP11-343N15.2 |  | 8.990339244 | 1.142211872 | 0.127048807 | -2.97654527 | 0.003511457 |
| ENSG00000163239 | TDRD10 | tudor domain containing 10 | 21.41486565 | 2.796522145 | 0.130587891 | -2.936906966 | 0.00021248 |
| ENSG00000188095 | MESP2 | mesoderm posterior bHLH transcription factor 2 | 6.783773511 | 0.918666618 | 0.135421181 | -2.884474693 | 0.049022417 |
| ENSG00000152580 | IGSF10 | immunoglobulin superfamily, member 10 | 1145.312459 | 169.1696245 | 0.147706089 | -2.75919879 | 0.047429995 |
| ENSG00000225007 | AC000067.1 |  | 5.658051045 | 0.836118353 | 0.147774975 | -2.758526116 | 0.02157232 |
| ENSG00000232560 | LINC01549 | long intergenic non-protein coding RNA 1549 | 12.31446061 | 1.858151246 | 0.15089181 | -2.728413596 | 0.002952257 |
| ENSG00000149418 | ST14 | suppression of tumorigenicity 14 (colon carcinoma) | 5.388387367 | 0.8432644 | 0.156496618 | -2.675796615 | 0.031457288 |
| ENSG00000251450 | RASGRF2-AS1 | RASGRF2 antisense RNA 1 | 8.484319464 | 1.367413944 | 0.161169549 | -2.633348902 | 0.013995857 |
| ENSG00000247498 | RP11-392P7.6 |  | 33.74842631 | 5.440557662 | 0.161209225 | -2.632993789 | 0.039173817 |
| ENSG00000227053 | RP11-395B7.4 |  | 4.602357559 | 0.760206295 | 0.165177582 | -2.597910194 | 0.045162992 |
| ENSG00000152936 | LMNTD1 | lamin tail domain containing 1 | 22.92945722 | 3.799083681 | 0.165685722 | -2.593478809 | 0.004182218 |
| ENSG00000249231 | CASC16 | cancer susceptibility candidate 16 (non-protein coding) | 4.908385089 | 0.816414072 | 0.166330485 | -2.587875486 | 0.035414816 |
| ENSG00000169291 | SHE | Src homology 2 domain containing E | 43.58391487 | 7.409744559 | 0.170010991 | -2.556300077 | 0.011295696 |
| ENSG00000230313 | HCG24 | HLA complex group 24 (non-protein coding) | 15.45139629 | 2.686009824 | 0.173836058 | -2.524200729 | 0.049168726 |
| ENSG00000184459 | BPIFC | BPI fold containing family C | 9.408578781 | 1.642865767 | 0.174613595 | -2.517762205 | 0.008810324 |
| ENSG00000130176 | CNN1 | calponin 1, basic, smooth muscle | 352.1815394 | 61.99826078 | 0.176040632 | -2.506019638 | 0.01903944 |
| ENSG00000171227 | TMEM37 | transmembrane protein 37 | 44.86469261 | 8.084558894 | 0.180198691 | -2.472339561 | 0.009763147 |
| ENSG00000148702 | HABP2 | hyaluronan binding protein 2 | 19.67066349 | 3.632935831 | 0.184688017 | -2.436837832 | 0.000278269 |
| ENSG00000187758 | ADH1A | alcohol dehydrogenase 1A (class I), alpha polypeptide | 14.06499596 | 2.683831283 | 0.190816357 | -2.389743252 | 0.041577708 |
| ENSG00000223489 | NEFHP1 | neurofilament, heavy polypeptide pseudogene 1 | 14.76340234 | 2.819570362 | 0.190983778 | -2.388477989 | 0.003207999 |
| ENSG00000235884 | LINC00941 | long intergenic non-protein coding RNA 941 | 7.054914483 | 1.355085885 | 0.192076869 | -2.380244303 | 0.04141072 |
| ENSG00000239605 | C2orf61 | chromosome 2 open reading frame 61 | 7.542472102 | 1.458962 | 0.193432867 | -2.370095148 | 0.015471207 |
| ENSG00000167157 | PRRX2 | paired related homeobox 2 | 114.2413685 | 22.2644839 | 0.194889856 | -2.359269092 | 0.030346863 |
| ENSG00000226278 | PSPHP1 | phosphoserine phosphatase pseudogene 1 | 200.2678637 | 40.10248172 | 0.200244218 | -2.320167511 | 0.040656873 |
| ENSG00000226539 | AC012512.1 |  | 6.58883404 | 1.319975782 | 0.20033526 | -2.319511728 | 0.039127926 |
| ENSG00000163661 | PTX3 | pentraxin 3, long | 5040.195479 | 1035.371274 | 0.205422841 | -2.283331491 | 0.047671926 |
| ENSG00000143248 | RGS5 | regulator of G-protein signaling 5 | 539.042985 | 110.8348923 | 0.2056142 | -2.281988191 | 0.01172852 |
| ENSG00000178222 | RNF212 | ring finger protein 212 | 112.5114775 | 23.31149447 | 0.207192146 | -2.270958779 | 0.0167786 |
| ENSG00000254872 | RP13-870H17.3 |  | 10.73500283 | 2.242880631 | 0.208931536 | -2.258897827 | 0.010387483 |
| ENSG00000236090 | LDHAP3 | lactate dehydrogenase A pseudogene 3 | 64.28398496 | 13.49022532 | 0.209853595 | -2.25254492 | 0.000358855 |
| ENSG00000180353 | HCLS1 | hematopoietic cell-specific Lyn substrate 1 | 16.8558744 | 3.574424698 | 0.2120581 | -2.237468505 | 0.038839102 |
| ENSG00000118523 | CTGF | connective tissue growth factor | 7619.724875 | 1640.699927 | 0.215322725 | -2.215427504 | 0.004610977 |
| ENSG00000237512 | UNC5B-AS1 | UNC5B antisense RNA 1 | 12.10290058 | 2.608319918 | 0.215511968 | -2.214160108 | 0.005719645 |
| ENSG00000255222 | SETP17 | SET pseudogene 17 | 7.201500957 | 1.566846169 | 0.217572167 | -2.200434084 | 0.032273306 |
| ENSG00000232177 | MTND4P24 | MT-ND4 pseudogene 24 | 68.07952596 | 14.89031008 | 0.218719356 | -2.192847194 | 0.021004213 |
| ENSG00000137757 | CASP5 | caspase 5, apoptosis-related cysteine peptidase | 5.918278079 | 1.307647724 | 0.220950707 | -2.17820355 | 0.034299117 |
| ENSG00000120156 | TEK | TEK tyrosine kinase, endothelial | 754.6999363 | 167.677725 | 0.222178003 | -2.170212104 | 0.042064223 |
| ENSG00000234883 | MIR155HG | MIR155 host gene | 8.229913889 | 1.844092078 | 0.224071856 | -2.157966642 | 0.045965778 |
| ENSG00000168685 | IL7R | interleukin 7 receptor | 12.87317563 | 2.919161508 | 0.226763123 | -2.140742052 | 0.037686797 |
| ENSG00000133105 | RXFP2 | relaxin/insulin-like family peptide receptor 2 | 13.073358 | 3.018615384 | 0.230898242 | -2.114670904 | 0.02581266 |
| ENSG00000259207 | ITGB3 | integrin, beta 3 (platelet glycoprotein IIIa, antigen CD61) | 339.7858317 | 80.7969046 | 0.237787739 | -2.072253768 | 0.011571393 |
| ENSG00000267882 | RP4-569M23.5 |  | 7.613671487 | 1.815230979 | 0.238417297 | -2.068439187 | 0.035944576 |
| ENSG00000197980 | LEKR1 | leucine, glutamate and lysine rich 1 | 9.660078146 | 2.307748877 | 0.238895467 | -2.065548618 | 0.024052263 |
| ENSG00000183571 | PGPEP1L | pyroglutamyl-peptidase I-like | 8.189868391 | 1.976815803 | 0.24137333 | -2.05066182 | 0.034550197 |
| ENSG00000223651 | RP5-1121H13.1 |  | 10.06760895 | 2.443997796 | 0.242758515 | -2.042406195 | 0.021087617 |
| ENSG00000169618 | PROKR1 | prokineticin receptor 1 | 141.5696542 | 34.59683846 | 0.244380328 | -2.032799941 | 0.038849268 |
| ENSG00000188501 | LCTL | lactase-like | 142.4357523 | 34.95457568 | 0.245405912 | -2.02675809 | 0.039131427 |
| ENSG00000102760 | RGCC | regulator of cell cycle | 291.6418315 | 72.20841801 | 0.247592801 | -2.013958727 | 0.025367345 |
| ENSG00000063515 | GSC2 | goosecoid homeobox 2 | 8.486052985 | 2.102406971 | 0.247748509 | -2.013051722 | 0.028751214 |
| ENSG00000258230 | RP11-511H9.3 |  | 7.429343633 | 1.855523094 | 0.249755993 | -2.0014088 | 0.038627248 |
| ENSG00000006128 | TAC1 | tachykinin, precursor 1 | 9908.007864 | 2494.84013 | 0.251800378 | -1.989647643 | 0.002477374 |
| ENSG00000147883 | CDKN2B | cyclin-dependent kinase inhibitor 2B (p15, inhibits CDK4) | 526.1058416 | 135.8829697 | 0.258280671 | -1.952988416 | 0.037100964 |
| ENSG00000137801 | THBS1 | thrombospondin 1 | 6591.95897 | 1719.053494 | 0.260780369 | -1.939092824 | 0.042925447 |
| ENSG00000228313 |  |  | 10.50007529 | 2.760794662 | 0.26293094 | -1.927244177 | 0.029541618 |
| ENSG00000171345 | KRT19 | keratin 19, type I | 23.06707524 | 6.085240141 | 0.263806316 | -1.922448989 | 0.016421044 |
| ENSG00000112499 | SLC22A2 | solute carrier family 22 (organic cation transporter), member 2 | 9.61265029 | 2.553768959 | 0.26566752 | -1.91230624 | 0.036701176 |
| ENSG00000100311 | PDGFB | platelet-derived growth factor beta polypeptide | 687.0097382 | 182.9183322 | 0.266252896 | -1.909130879 | 0.037319385 |
| ENSG00000134007 | ADAM20 | ADAM metallopeptidase domain 20 | 20.45884023 | 5.476658285 | 0.267691532 | -1.901356592 | 0.007435009 |
| ENSG00000254768 | CTD-2140G10.2 |  | 194.463804 | 52.46706336 | 0.269803749 | -1.8900177 | 0.001032923 |
| ENSG00000222041 | LINC00152 | long intergenic non-protein coding RNA 152 | 204.6684097 | 55.52532592 | 0.27129407 | -1.88207058 | 0.028728555 |
| ENSG00000110975 | SYT10 | synaptotagmin X | 405.4373625 | 110.0603048 | 0.271460687 | -1.881184815 | 0.036490593 |
| ENSG00000141934 | PLPP2 | phospholipid phosphatase 2 | 69.17356313 | 18.97167188 | 0.274261886 | -1.86637395 | 0.000694695 |
| ENSG00000144671 | SLC22A14 | solute carrier family 22, member 14 | 19.94772532 | 5.514336311 | 0.276439354 | -1.85496508 | 0.022346156 |
| ENSG00000232629 | HLA-DQB2 | major histocompatibility complex, class II, DQ beta 2 | 26.36645867 | 7.290369716 | 0.276501665 | -1.854639928 | 0.029157235 |
| ENSG00000259937 | RP11-438D14.2 |  | 17.8691845 | 4.987634513 | 0.279119314 | -1.84104614 | 0.013941848 |
| ENSG00000091664 | SLC17A6 | solute carrier family 17 (vesicular glutamate transporter), member 6 | 7836.69017 | 2208.499828 | 0.281815381 | -1.82717774 | 0.007710461 |
| ENSG00000227365 | AC008060.5 |  | 6.43643551 | 1.824000594 | 0.283386758 | -1.819155748 | 0.03942798 |
| ENSG00000259378 | DCAF13P3 | DDB1 and CUL4 associated factor 13 pseudogene 3 | 26.64009436 | 7.560427965 | 0.283798843 | -1.817059386 | 0.003093099 |
| ENSG00000154065 | ANKRD29 | ankyrin repeat domain 29 | 407.1410961 | 116.066735 | 0.285077424 | -1.810574301 | 0.032432102 |
| ENSG00000229585 | RPL21P44 | ribosomal protein L21 pseudogene 44 | 12.02373316 | 3.434697321 | 0.285659809 | -1.807630024 | 0.03965468 |
| ENSG00000157765 | SLC34A2 | solute carrier family 34 (type II sodium/phosphate cotransporter), member 2 | 87.30599499 | 25.10668964 | 0.287571199 | -1.798008902 | 0.023022455 |
| ENSG00000113600 | C9 | complement component 9 | 9.941830373 | 2.920275237 | 0.293736176 | -1.767407138 | 0.033966724 |
| ENSG00000250292 | RP11-451F20.1 |  | 8.530731455 | 2.506793898 | 0.293854508 | -1.766826062 | 0.041925178 |
| ENSG00000250331 | LINC01340 | long intergenic non-protein coding RNA 1340 | 19.16290982 | 5.643515082 | 0.29450199 | -1.763650711 | 0.007705855 |
| ENSG00000214922 | HLA-F-AS1 | HLA-F antisense RNA 1 | 13.23152925 | 3.901289489 | 0.294847966 | -1.761956852 | 0.027147308 |
| ENSG00000257527 | MIR3179-3 |  | 7.920310874 | 2.341574733 | 0.295641771 | -1.758077974 | 0.045477985 |
| ENSG00000237194 | SNAI1P1 | snail family zinc finger 1 pseudogene 1 | 13.63604612 | 4.035376303 | 0.295934486 | -1.756650267 | 0.028076367 |
| ENSG00000124249 | KCNK15 | potassium channel, two pore domain subfamily K, member 15 | 8.460605355 | 2.530906928 | 0.29914017 | -1.741106437 | 0.038185879 |
| ENSG00000269067 | ZNF728 | zinc finger protein 728 | 160.0355716 | 48.09215019 | 0.300509129 | -1.734519278 | 0.00379116 |
| ENSG00000268758 | ADGRE4P | adhesion G protein-coupled receptor E4, pseudogene | 10.10550787 | 3.055782998 | 0.30238787 | -1.725527828 | 0.033974941 |
| ENSG00000253741 | CTD-2292P10.4 |  | 35.12251517 | 10.6237561 | 0.302477088 | -1.725102231 | 0.026677884 |
| ENSG00000255372 | CTD-3064C13.1 |  | 56.29152182 | 17.16985788 | 0.305016765 | -1.713039553 | 0.002753354 |
| ENSG00000115956 | PLEK | pleckstrin | 12.37291952 | 3.782843224 | 0.305735701 | -1.709643068 | 0.02290163 |
| ENSG00000172965 | MIR4435-2HG | MIR4435-2 host gene | 423.9911733 | 131.6692364 | 0.310547117 | -1.687115922 | 0.031789216 |
| ENSG00000258521 | RP11-638I2.9 |  | 16.54556049 | 5.16142201 | 0.31195208 | -1.680603666 | 0.012901414 |
| ENSG00000260799 | KRT8P50 | keratin 8 pseudogene 50 | 9.605507998 | 3.002979386 | 0.312630981 | -1.677467343 | 0.038912684 |
| ENSG00000042062 | FAM65C | family with sequence similarity 65, member C | 270.5514708 | 85.10291826 | 0.314553523 | -1.668622575 | 0.048785026 |
| ENSG00000118785 | SPP1 | secreted phosphoprotein 1 | 1955.722104 | 619.5140756 | 0.316769992 | -1.65849242 | 0.004696785 |
| ENSG00000092068 | SLC7A8 | solute carrier family 7 (amino acid transporter light chain, L system), member 8 | 1409.400717 | 448.4896773 | 0.318213033 | -1.651935169 | 0.01644057 |
| ENSG00000228692 | RP5-826L7.1 |  | 18.96093651 | 6.048832301 | 0.319015482 | -1.648301654 | 0.0113591 |
| ENSG00000104689 | TNFRSF10A | tumor necrosis factor receptor superfamily, member 10a | 24.44266031 | 7.901138756 | 0.323251997 | -1.629268812 | 0.029940834 |
| ENSG00000255277 | ABCC6P2 | ATP-binding cassette, sub-family C, member 6 pseudogene 2 | 23.07935355 | 7.464299607 | 0.32341892 | -1.628524015 | 0.039875015 |
| ENSG00000139549 | DHH | desert hedgehog | 11.90127421 | 3.865451717 | 0.324793098 | -1.622407121 | 0.031338707 |
| ENSG00000179256 | SMCO3 | single-pass membrane protein with coiled-coil domains 3 | 18.33525782 | 6.000467937 | 0.327263898 | -1.611473636 | 0.026878628 |
| ENSG00000236714 | AC005592.1 |  | 25.93384419 | 8.512641918 | 0.328244508 | -1.607157223 | 0.017084851 |
| ENSG00000230499 | AC108463.1 |  | 11.382613 | 3.786138243 | 0.332624701 | -1.588032788 | 0.038144095 |
| ENSG00000164106 | SCRG1 | stimulator of chondrogenesis 1 | 1403.500244 | 469.4670566 | 0.33449731 | -1.579933485 | 0.047541192 |
| ENSG00000100285 | NEFH | neurofilament, heavy polypeptide | 1090.203562 | 366.04294 | 0.335756507 | -1.574512735 | 0.026393968 |
| ENSG00000170345 | FOS | FBJ murine osteosarcoma viral oncogene homolog | 902.2141594 | 308.0266695 | 0.341411921 | -1.550414661 | 0.029469826 |
| ENSG00000128564 | VGF | VGF nerve growth factor inducible | 1107.710188 | 380.1864169 | 0.343218308 | -1.542801582 | 0.000204313 |
| ENSG00000138772 | ANXA3 | annexin A3 | 132.2553538 | 45.43548979 | 0.343543671 | -1.541434589 | 0.034852495 |
| ENSG00000266933 | AC005775.2 |  | 14.68873363 | 5.061627669 | 0.344592515 | -1.537036729 | 0.032378387 |
| ENSG00000188993 | LRRC66 | leucine rich repeat containing 66 | 82.15148204 | 28.33095575 | 0.344862382 | -1.53590733 | 0.001717247 |
| ENSG00000164344 | KLKB1 | kallikrein B, plasma (Fletcher factor) 1 | 23.4630209 | 8.102005988 | 0.345309584 | -1.534037718 | 0.013252289 |
| ENSG00000197355 | UAP1L1 | UDP-N-acetylglucosamine pyrophosphorylase 1 like 1 | 954.8628586 | 331.0179697 | 0.346665457 | -1.528384005 | 0.001131664 |
| ENSG00000264015 | RP11-176N18.2 |  | 43.74726101 | 15.28192656 | 0.349323048 | -1.517366261 | 0.005605221 |
| ENSG00000144115 | THNSL2 | threonine synthase-like 2 | 763.0640544 | 267.2822894 | 0.350275036 | -1.513439922 | 0.00540395 |
| ENSG00000246363 | RP11-13A1.1 |  | 17.82333298 | 6.256922817 | 0.351052344 | -1.510241935 | 0.035230789 |
| ENSG00000126861 | OMG | oligodendrocyte myelin glycoprotein | 613.0083817 | 215.2619086 | 0.35115655 | -1.509813748 | 0.0011905 |
| ENSG00000266497 | RP11-995C19.2 |  | 14.22989453 | 5.034111045 | 0.353770088 | -1.499116023 | 0.04677019 |
| ENSG00000177459 | ERICH5 | glutamate-rich 5 | 100.1347496 | 35.56312336 | 0.355152667 | -1.493488777 | 0.016115805 |
| ENSG00000081923 | ATP8B1 | ATPase, aminophospholipid transporter, class I, type 8B, member 1 | 72.89431944 | 25.9069043 | 0.35540361 | -1.492469758 | 0.029218353 |
| ENSG00000104361 | NIPAL2 | NIPA-like domain containing 2 | 417.716048 | 150.4664735 | 0.360212336 | -1.473080505 | 0.041933701 |
| ENSG00000242349 | NPPA-AS1 | NPPA antisense RNA 1 | 24.3973707 | 8.796261073 | 0.360541354 | -1.471763348 | 0.008123616 |
| ENSG00000158748 | HTR6 | 5-hydroxytryptamine (serotonin) receptor 6, G protein-coupled | 24.05946451 | 8.68986274 | 0.361182716 | -1.469199239 | 0.013624784 |
| ENSG00000188039 | NWD1 | NACHT and WD repeat domain containing 1 | 235.8799844 | 85.72504441 | 0.363426531 | -1.460264352 | 0.041544258 |
| ENSG00000112812 | PRSS16 | protease, serine, 16 (thymus) | 115.4458424 | 42.32724727 | 0.366641591 | -1.447557646 | 0.001492773 |
| ENSG00000164619 | BMPER | BMP binding endothelial regulator | 387.575725 | 144.1556026 | 0.371941774 | -1.426851305 | 0.044770519 |
| ENSG00000266369 | RP11-344E13.4 |  | 24.30387708 | 9.056155279 | 0.372621835 | -1.424215878 | 0.023809371 |
| ENSG00000182057 | OGFRP1 | opioid growth factor receptor pseudogene 1 | 14.72387606 | 5.497166132 | 0.373350476 | -1.421397525 | 0.03736774 |
| ENSG00000227825 | SLC9A7P1 | solute carrier family 9, subfamily A (NHE7, cation proton antiporter 7), member 7 pseudogene 1 | 14.45773756 | 5.420357031 | 0.374910459 | -1.41538202 | 0.029070032 |
| ENSG00000228222 | AC074363.1 |  | 28.70720952 | 10.79107951 | 0.375901374 | -1.411573905 | 0.019474618 |
| ENSG00000139410 | SDSL | serine dehydratase-like | 344.7910071 | 129.7747583 | 0.376386726 | -1.409712345 | 0.015036715 |
| ENSG00000135046 | ANXA1 | annexin A1 | 3617.876821 | 1363.239796 | 0.376806581 | -1.408103932 | 0.024053162 |
| ENSG00000232755 |  |  | 55.26355224 | 20.87372612 | 0.377712349 | -1.404640144 | 0.031997858 |
| ENSG00000206532 | RP11-553A10.1 |  | 200.2154759 | 75.69198428 | 0.378052615 | -1.40334106 | 0.035687983 |
| ENSG00000238021 | ARMC4P1 | armadillo repeat containing 4 pseudogene 1 | 48.36002198 | 18.4685328 | 0.3818967 | -1.388745642 | 0.010097942 |
| ENSG00000246898 | LINC00920 | long intergenic non-protein coding RNA 920 | 77.80265738 | 29.73673473 | 0.382207186 | -1.387573192 | 0.033307829 |
| ENSG00000181215 | C4orf50 | chromosome 4 open reading frame 50 | 226.4738011 | 86.73857198 | 0.382996053 | -1.38459857 | 0.022577902 |
| ENSG00000223477 |  |  | 21.39095309 | 8.208342486 | 0.383729629 | -1.381837932 | 0.047239466 |
| ENSG00000260910 | LINC00565 | long intergenic non-protein coding RNA 565 | 85.05190552 | 32.90085 | 0.386832603 | -1.370218702 | 0.003118473 |
| ENSG00000075073 | TACR2 | tachykinin receptor 2 | 46.06328973 | 17.8640444 | 0.387815211 | -1.366558706 | 0.007886936 |
| ENSG00000205596 | RP5-1132H15.2 |  | 26.62505086 | 10.37697139 | 0.38974466 | -1.359398839 | 0.035647388 |
| ENSG00000204929 | AC074391.1 |  | 36.33184561 | 14.26355831 | 0.392591075 | -1.348900721 | 0.013613271 |
| ENSG00000235448 | LURAP1L-AS1 | LURAP1L antisense RNA 1 | 53.39658844 | 20.99657808 | 0.393219468 | -1.346593346 | 0.007362522 |
| ENSG00000237892 | KLF7-IT1 | KLF7 intronic transcript 1 | 23.92470204 | 9.459320937 | 0.39537884 | -1.338692433 | 0.03321988 |
| ENSG00000205663 | RP11-706O15.5 |  | 2002.67617 | 794.4534263 | 0.3966959 | -1.333894607 | 0.00192684 |
| ENSG00000238290 | RP11-431K24.1 |  | 23.19390298 | 9.23926763 | 0.398348982 | -1.327895209 | 0.049801363 |
| ENSG00000183346 | C10orf107 | chromosome 10 open reading frame 107 | 84.96038212 | 33.92657367 | 0.399322282 | -1.324374519 | 0.040111808 |
| ENSG00000139278 | GLIPR1 | GLI pathogenesis-related 1 | 782.7826077 | 312.9929747 | 0.399846613 | -1.322481426 | 0.007195258 |
| ENSG00000081052 | COL4A4 | collagen, type IV, alpha 4 | 67.65009055 | 27.12863445 | 0.401014015 | -1.318275436 | 0.007541179 |
| ENSG00000248027 | CTD-2383M3.1 |  | 26.22451421 | 10.62084323 | 0.404996758 | -1.304017736 | 0.037019254 |
| ENSG00000248596 | RP11-844P9.2 |  | 30.39501537 | 12.3176025 | 0.405250741 | -1.303113272 | 0.010959287 |
| ENSG00000156466 | GDF6 | growth differentiation factor 6 | 145.3810642 | 58.91981275 | 0.405278453 | -1.303014621 | 0.008055791 |
| ENSG00000234449 | RP11-706O15.3 |  | 4203.754418 | 1718.63791 | 0.408834042 | -1.290412766 | 0.001449036 |
| ENSG00000131471 | AOC3 | amine oxidase, copper containing 3 | 217.8253456 | 89.35519017 | 0.410214844 | -1.285548397 | 0.030345003 |
| ENSG00000236914 | RP11-1008C21.2 |  | 70.86374018 | 29.23195765 | 0.412509382 | -1.277501164 | 0.009639243 |
| ENSG00000206341 | HLA-H | major histocompatibility complex, class I, H (pseudogene) | 611.4556475 | 252.9436454 | 0.413674559 | -1.27343186 | 0.015695468 |
| ENSG00000187678 | SPRY4 | sprouty RTK signaling antagonist 4 | 1944.51286 | 811.7987235 | 0.417481797 | -1.2602148 | 0.045923327 |
| ENSG00000060140 | STYK1 | serine/threonine/tyrosine kinase 1 | 192.0537097 | 80.2867504 | 0.418043216 | -1.258276004 | 0.017990784 |
| ENSG00000081148 | IMPG2 | interphotoreceptor matrix proteoglycan 2 | 151.6962843 | 63.45216937 | 0.418284269 | -1.257444354 | 0.009433782 |
| ENSG00000164761 | TNFRSF11B | tumor necrosis factor receptor superfamily, member 11b | 239.1172592 | 100.0779678 | 0.418530925 | -1.256593867 | 0.043503469 |
| ENSG00000235847 | LDHAP7 | lactate dehydrogenase A pseudogene 7 | 151.5527146 | 65.00709558 | 0.428940489 | -1.22115059 | 0.014709819 |
| ENSG00000183773 | AIFM3 | apoptosis-inducing factor, mitochondrion-associated, 3 | 81.48398838 | 35.04607436 | 0.43009768 | -1.217263746 | 0.010760765 |
| ENSG00000106066 | CPVL | carboxypeptidase, vitellogenic-like | 636.7176901 | 274.2404472 | 0.43070964 | -1.215212481 | 0.046393366 |
| ENSG00000266865 | RP11-848P1.9 |  | 45.19871871 | 19.63835182 | 0.434489127 | -1.202608021 | 0.025409486 |
| ENSG00000238243 | OR2W3 | olfactory receptor, family 2, subfamily W, member 3 | 88.07177221 | 38.31823416 | 0.435079631 | -1.200648618 | 0.042940176 |
| ENSG00000236558 |  |  | 28.77387497 | 12.61599225 | 0.438453015 | -1.189505843 | 0.025581468 |
| ENSG00000248202 | RP11-455B3.1 |  | 22.81178651 | 10.00710008 | 0.438681121 | -1.188755474 | 0.041706085 |
| ENSG00000160307 | S100B | S100 calcium binding protein B | 1956.826915 | 859.7887491 | 0.439379049 | -1.186462016 | 0.004848701 |
| ENSG00000244405 | ETV5 | ets variant 5 | 2248.181415 | 990.0855866 | 0.440393991 | -1.183133309 | 0.00514616 |
| ENSG00000241679 | RP11-80H8.4 |  | 23.70090532 | 10.55653936 | 0.445406588 | -1.166805199 | 0.046097541 |
| ENSG00000189280 | GJB5 | gap junction protein, beta 5, 31.1kDa | 17.70117223 | 7.9464056 | 0.448919738 | -1.155470565 | 0.049285646 |
| ENSG00000143466 |  |  | 263.3889616 | 118.8793477 | 0.451345216 | -1.14769678 | 0.028299781 |
| ENSG00000179344 | HLA-DQB1 | major histocompatibility complex, class II, DQ beta 1 | 106.1559096 | 47.98369384 | 0.452011518 | -1.145568559 | 0.044489107 |
| ENSG00000170122 | FOXD4 | forkhead box D4 | 112.8998218 | 51.16664927 | 0.453203986 | -1.141767545 | 0.018192005 |
| ENSG00000228203 | RNF144A-AS1 | RNF144A antisense RNA 1 | 638.0390663 | 289.2393581 | 0.453325468 | -1.141380881 | 0.00907533 |
| ENSG00000166592 | RRAD | Ras-related associated with diabetes | 41.39254151 | 18.83786908 | 0.455102982 | -1.135735055 | 0.018579649 |
| ENSG00000162722 | TRIM58 | tripartite motif containing 58 | 219.4291629 | 100.3488026 | 0.457317529 | -1.128731876 | 0.031927842 |
| ENSG00000246523 | RP11-736K20.6 |  | 32.63538653 | 14.99218405 | 0.459384296 | -1.122226557 | 0.033374397 |
| ENSG00000203724 | C1orf53 | chromosome 1 open reading frame 53 | 51.55612747 | 23.69687544 | 0.459632571 | -1.121447058 | 0.035258161 |
| ENSG00000266709 | RP11-214O1.2 |  | 180.3731223 | 83.15072052 | 0.460992854 | -1.117183706 | 0.009632473 |
| ENSG00000118508 | RAB32 | RAB32, member RAS oncogene family | 705.2863136 | 325.2042278 | 0.461095333 | -1.116863031 | 0.041896152 |
| ENSG00000196353 | CPNE4 | copine IV | 1639.187576 | 758.8385426 | 0.462935758 | -1.111116093 | 0.010536611 |
| ENSG00000255406 | RP11-713P17.5 |  | 33.3850596 | 15.54688706 | 0.465683969 | -1.102576876 | 0.031858709 |
| ENSG00000175040 | CHST2 | carbohydrate (N-acetylglucosamine-6-O) sulfotransferase 2 | 3409.593211 | 1599.450535 | 0.469103038 | -1.09202325 | 0.017524468 |
| ENSG00000163235 | TGFA | transforming growth factor, alpha | 131.0778485 | 61.64035179 | 0.47025758 | -1.088476897 | 0.018245225 |
| ENSG00000138207 | RBP4 | retinol binding protein 4, plasma | 61.08047711 | 28.74786699 | 0.470655574 | -1.087256415 | 0.018426638 |
| ENSG00000183671 | GPR1 | G protein-coupled receptor 1 | 566.7295154 | 266.7767607 | 0.47073031 | -1.087027344 | 0.037315655 |
| ENSG00000263958 | RP11-676J15.1 |  | 36.16185264 | 17.07366521 | 0.472145755 | -1.082695796 | 0.042880998 |
| ENSG00000180318 | ALX1 | ALX homeobox 1 | 98.97698501 | 46.78003559 | 0.472635488 | -1.081200137 | 0.029343984 |
| ENSG00000185742 | C11orf87 | chromosome 11 open reading frame 87 | 4265.305901 | 2021.020887 | 0.473827888 | -1.077564981 | 0.007979133 |
| ENSG00000197406 | DIO3 | deiodinase, iodothyronine, type III | 380.2283794 | 180.3125512 | 0.474221707 | -1.076366392 | 0.010186479 |
| ENSG00000113749 | HRH2 | histamine receptor H2 | 72.59004594 | 34.57576579 | 0.476315524 | -1.070010525 | 0.033420091 |
| ENSG00000171488 | LRRC8C | leucine rich repeat containing 8 family, member C | 772.8671972 | 368.3634476 | 0.476619332 | -1.069090624 | 0.008320898 |
| ENSG00000071282 | LMCD1 | LIM and cysteine-rich domains 1 | 490.7783404 | 234.4904336 | 0.477792955 | -1.065542513 | 0.030278948 |
| ENSG00000197445 | C16orf47 | chromosome 16 open reading frame 47 | 54.65465586 | 26.16814666 | 0.478790805 | -1.06253265 | 0.027169354 |
| ENSG00000214960 | ISPD | isoprenoid synthase domain containing | 110.2364988 | 53.47866771 | 0.485126689 | -1.043566543 | 0.031001919 |
| ENSG00000161249 | DMKN | dermokine | 79.57335055 | 38.79797256 | 0.487574952 | -1.036304083 | 0.027513663 |
| ENSG00000075429 | CACNG5 | calcium channel, voltage-dependent, gamma subunit 5 | 357.8277173 | 174.5955772 | 0.487931954 | -1.035248129 | 0.030198228 |
| ENSG00000163803 | PLB1 | phospholipase B1 | 227.6811169 | 111.6538551 | 0.490395763 | -1.02798158 | 0.013043853 |
| ENSG00000182253 | SYNM | synemin, intermediate filament protein | 5396.562314 | 2652.059457 | 0.491434973 | -1.024927563 | 0.039926899 |
| ENSG00000245812 | RP11-175K6.1 |  | 83.44474738 | 41.04846146 | 0.491923851 | -1.023493089 | 0.019796716 |
| ENSG00000259705 | RP11-227D13.1 |  | 51.64793823 | 25.48657519 | 0.493467427 | -1.018973237 | 0.047440642 |
| ENSG00000225206 | MIR137HG | MIR137 host gene | 133.3681908 | 65.86500146 | 0.493858401 | -1.017830643 | 0.033544658 |
| ENSG00000185567 | AHNAK2 | AHNAK nucleoprotein 2 | 6674.460295 | 3299.787676 | 0.494390187 | -1.016277986 | 0.020285393 |
| ENSG00000264660 | RP11-381P6.1 |  | 68.31755537 | 33.91225287 | 0.496391487 | -1.010449722 | 0.047546494 |
| ENSG00000254510 | RP11-867G23.10 |  | 183.1368886 | 90.98433226 | 0.496810517 | -1.009232382 | 0.012508923 |
| ENSG00000231028 | LINC00271 | long intergenic non-protein coding RNA 271 | 76.79407423 | 38.30629594 | 0.498818383 | -1.00341346 | 0.026935911 |
| ENSG00000072952 | MRVI1 | murine retrovirus integration site 1 homolog | 702.0509587 | 351.2014812 | 0.500250697 | -0.999276824 | 0.013517458 |
| ENSG00000116745 | RPE65 | retinal pigment epithelium-specific protein 65kDa | 1024.936705 | 513.990893 | 0.501485497 | -0.995720116 | 0.040663026 |
| ENSG00000196083 | IL1RAP | interleukin 1 receptor accessory protein | 997.6500525 | 500.3707533 | 0.501549368 | -0.995536381 | 0.025343181 |
| ENSG00000184492 | FOXD4L1 | forkhead box D4-like 1 | 116.3265291 | 58.42264626 | 0.502229773 | -0.99358054 | 0.036794533 |
| ENSG00000231752 | EMBP1 | embigin pseudogene 1 | 68.9709051 | 35.17308704 | 0.509969921 | -0.971515938 | 0.048383756 |
| ENSG00000156486 | KCNS2 | potassium voltage-gated channel, modifier subfamily S, member 2 | 895.9776158 | 457.0014739 | 0.51005903 | -0.971263872 | 0.042953179 |
| ENSG00000183935 | HTR7P1 | 5-hydroxytryptamine (serotonin) receptor 7 pseudogene 1 | 433.0303312 | 221.9085064 | 0.51245488 | -0.964503109 | 0.018650773 |
| ENSG00000254122 | PCDHGB7 | protocadherin gamma subfamily B, 7 | 3877.758177 | 1996.607476 | 0.514887052 | -0.957672104 | 0.022767099 |
| ENSG00000087253 | LPCAT2 | lysophosphatidylcholine acyltransferase 2 | 470.9264492 | 242.8901535 | 0.515770889 | -0.955197747 | 0.03225245 |
| ENSG00000158406 | HIST1H4H | histone cluster 1, H4h | 130.175625 | 67.97973906 | 0.522215577 | -0.937282604 | 0.047699628 |
| ENSG00000144452 | ABCA12 | ATP-binding cassette, sub-family A (ABC1), member 12 | 115.0498002 | 60.40910665 | 0.525069201 | -0.929420522 | 0.033424604 |
| ENSG00000132329 | RAMP1 | receptor (G protein-coupled) activity modifying protein 1 | 247.6350989 | 130.2683812 | 0.526049747 | -0.926728857 | 0.028301656 |
| ENSG00000064601 | CTSA | cathepsin A | 3459.146124 | 1838.363951 | 0.531450215 | -0.911993545 | 0.019578855 |
| ENSG00000149972 | CNTN5 | contactin 5 | 2015.050444 | 1073.897729 | 0.532938385 | -0.907959347 | 0.048589764 |
| ENSG00000254221 | PCDHGB1 | protocadherin gamma subfamily B, 1 | 308.7015413 | 164.658563 | 0.533390803 | -0.906735145 | 0.025199711 |
| ENSG00000185669 | SNAI3 | snail family zinc finger 3 | 208.7882742 | 112.1490817 | 0.537142625 | -0.896622883 | 0.045095454 |
| ENSG00000184261 | KCNK12 | potassium channel, two pore domain subfamily K, member 12 | 207.7294498 | 112.7301073 | 0.542677542 | -0.881832888 | 0.032014576 |
| ENSG00000185668 | POU3F1 | POU class 3 homeobox 1 | 1562.599065 | 852.3066376 | 0.545441666 | -0.874503183 | 0.024600456 |
| ENSG00000138759 | FRAS1 | Fraser extracellular matrix complex subunit 1 | 5735.120606 | 3137.153711 | 0.547007452 | -0.870367607 | 0.042248079 |
| ENSG00000166750 | SLFN5 | schlafen family member 5 | 1971.872151 | 1086.810537 | 0.551156694 | -0.859465558 | 0.042407481 |
| ENSG00000134996 | OSTF1 | osteoclast stimulating factor 1 | 360.391379 | 198.8778022 | 0.5518384 | -0.857682243 | 0.036906215 |
| ENSG00000151491 | EPS8 | epidermal growth factor receptor pathway substrate 8 | 5671.113175 | 3156.551358 | 0.556601722 | -0.845282723 | 0.02689567 |
| ENSG00000151623 | NR3C2 | nuclear receptor subfamily 3, group C, member 2 | 586.8349442 | 331.8859693 | 0.565552499 | -0.822267142 | 0.030817591 |
| ENSG00000144959 | NCEH1 | neutral cholesterol ester hydrolase 1 | 498.2687866 | 282.2262385 | 0.566413643 | -0.82007208 | 0.038364002 |
| ENSG00000176697 | BDNF | brain-derived neurotrophic factor | 412.850255 | 234.3536762 | 0.567648132 | -0.81693117 | 0.035113148 |
| ENSG00000249158 | PCDHA11 | protocadherin alpha 11 | 1007.664451 | 572.7387598 | 0.568382421 | -0.81506616 | 0.046630741 |
| ENSG00000119917 | IFIT3 | interferon-induced protein with tetratricopeptide repeats 3 | 513.6745517 | 292.1777893 | 0.568799424 | -0.81400809 | 0.036520884 |
| ENSG00000177628 | GBA | glucosidase, beta, acid | 3010.503668 | 1714.122254 | 0.569380557 | -0.812534866 | 0.034205248 |
| ENSG00000113070 | HBEGF | heparin-binding EGF-like growth factor | 1241.791043 | 707.6233647 | 0.569840932 | -0.811368841 | 0.04312668 |
| ENSG00000180573 | HIST1H2AC | histone cluster 1, H2ac | 1744.525849 | 997.1091259 | 0.571564547 | -0.807011664 | 0.049060942 |
| ENSG00000198756 | COLGALT2 | collagen beta(1-O)galactosyltransferase 2 | 3870.174024 | 2216.365028 | 0.57267839 | -0.804202931 | 0.037838356 |
| ENSG00000109686 | SH3D19 | SH3 domain containing 19 | 2033.068675 | 1173.353975 | 0.577134451 | -0.793020641 | 0.043319158 |
| ENSG00000182752 | PAPPA | pregnancy-associated plasma protein A, pappalysin 1 | 822.0265643 | 478.1998803 | 0.58173288 | -0.781571247 | 0.048078292 |
| ENSG00000145632 | PLK2 | polo-like kinase 2 | 7370.57532 | 4313.420082 | 0.585221627 | -0.772945009 | 0.037206762 |
| ENSG00000166342 | NETO1 | neuropilin (NRP) and tolloid (TLL)-like 1 | 1154.078131 | 692.8187605 | 0.600322233 | -0.736190996 | 0.046508273 |
| ENSG00000159200 | RCAN1 | regulator of calcineurin 1 | 7566.869678 | 4543.019553 | 0.600382952 | -0.736045084 | 0.049849017 |
| ENSG00000114805 | PLCH1 | phospholipase C, eta 1 | 2287.606227 | 4020.843892 | 1.757664342 | 0.813659588 | 0.045065475 |
| ENSG00000137857 | DUOX1 | dual oxidase 1 | 219.8676 | 387.8807525 | 1.76415603 | 0.818978165 | 0.046694892 |
| ENSG00000099954 | CECR2 | cat eye syndrome chromosome region, candidate 2 | 1455.064577 | 2587.630967 | 1.778361599 | 0.830548701 | 0.036045922 |
| ENSG00000188783 | PRELP | proline/arginine-rich end leucine-rich repeat protein | 99.93301536 | 180.5192838 | 1.806402851 | 0.853119668 | 0.04950286 |
| ENSG00000197044 | ZNF441 | zinc finger protein 441 | 873.0316742 | 1585.927166 | 1.816574602 | 0.861220614 | 0.040849866 |
| ENSG00000181085 | MAPK15 | mitogen-activated protein kinase 15 | 330.0875967 | 601.4500345 | 1.822092198 | 0.865595961 | 0.044296459 |
| ENSG00000141314 | RHBDL3 | rhomboid, veinlet-like 3 (Drosophila) | 2197.742397 | 4114.593332 | 1.872190908 | 0.904727554 | 0.034743087 |
| ENSG00000253230 | LINC00599 | long intergenic non-protein coding RNA 599 | 1497.208506 | 2805.938543 | 1.874113412 | 0.90620826 | 0.045550459 |
| ENSG00000165309 | ARMC3 | armadillo repeat containing 3 | 157.6255717 | 299.1329156 | 1.897743572 | 0.924285065 | 0.042068929 |
| ENSG00000145020 | AMT | aminomethyltransferase | 290.7803205 | 552.4331024 | 1.899829745 | 0.925870136 | 0.029444544 |
| ENSG00000182957 | SPATA13 | spermatogenesis associated 13 | 3509.871619 | 6739.494713 | 1.920154195 | 0.941222169 | 0.043372451 |
| ENSG00000118557 | PMFBP1 | polyamine modulated factor 1 binding protein 1 | 63.27698614 | 122.7478387 | 1.939849639 | 0.955944831 | 0.026474816 |
| ENSG00000253300 | RP11-108E14.1 |  | 31.87402699 | 62.43859414 | 1.958917653 | 0.970056752 | 0.041565803 |
| ENSG00000185522 | LMNTD2 | lamin tail domain containing 2 | 36.23468047 | 72.52847522 | 2.001631428 | 1.001176347 | 0.045074614 |
| ENSG00000167524 | SGK494 | uncharacterized serine/threonine-protein kinase SgK494 | 41.0513051 | 82.52448846 | 2.010276854 | 1.007394202 | 0.03688241 |
| ENSG00000163755 | HPS3 | Hermansky-Pudlak syndrome 3 | 598.1834484 | 1212.55809 | 2.02706727 | 1.019393967 | 0.012906439 |
| ENSG00000103184 | SEC14L5 | SEC14-like lipid binding 5 | 47.23643101 | 97.03019793 | 2.054139059 | 1.038533851 | 0.029761305 |
| ENSG00000197146 |  |  | 23.39982249 | 48.19695988 | 2.059714765 | 1.042444563 | 0.039112101 |
| ENSG00000247157 | LINC01252 | long intergenic non-protein coding RNA 1252 | 26.38033104 | 54.79915384 | 2.077273168 | 1.054690948 | 0.036487897 |
| ENSG00000259715 | CTD-3110H11.1 |  | 102.9682122 | 214.5006998 | 2.083173975 | 1.05878333 | 0.018771445 |
| ENSG00000137561 | TTPA | tocopherol (alpha) transfer protein | 44.33192936 | 93.02175383 | 2.098301499 | 1.06922199 | 0.031484978 |
| ENSG00000239704 | CDRT4 | CMT1A duplicated region transcript 4 | 24.23436248 | 51.44189886 | 2.122684222 | 1.085889767 | 0.035660042 |
| ENSG00000225889 | AC074289.1 |  | 75.88021868 | 161.2269629 | 2.124756171 | 1.087297292 | 0.024033933 |
| ENSG00000139364 | TMEM132B | transmembrane protein 132B | 5222.764097 | 11152.03697 | 2.135274878 | 1.094421803 | 0.040971559 |
| ENSG00000254815 | RP11-496I9.1 |  | 19.35218538 | 41.55228606 | 2.147162464 | 1.102431356 | 0.049823109 |
| ENSG00000101160 | CTSZ | cathepsin Z | 18.42997287 | 39.61603225 | 2.149543709 | 1.104030447 | 0.044344447 |
| ENSG00000213777 | CTD-2224J9.8 |  | 38.91864651 | 83.86080131 | 2.154771782 | 1.107535078 | 0.025420362 |
| ENSG00000204420 | C6orf25 | chromosome 6 open reading frame 25 | 31.74315438 | 69.00990331 | 2.174009 | 1.120357913 | 0.045405561 |
| ENSG00000225205 | AC093818.1 |  | 16.74931799 | 36.46597529 | 2.177161799 | 1.122448627 | 0.028540709 |
| ENSG00000177432 | NAP1L5 | nucleosome assembly protein 1-like 5 | 2212.658998 | 4846.468714 | 2.190336929 | 1.13115281 | 0.020456899 |
| ENSG00000226416 | MRPL23-AS1 | MRPL23 antisense RNA 1 | 47.46984832 | 104.2545173 | 2.196226047 | 1.135026552 | 0.02189921 |
| ENSG00000236233 |  |  | 44.2711832 | 97.52296233 | 2.202854211 | 1.139374018 | 0.027300186 |
| ENSG00000115353 | TACR1 | tachykinin receptor 1 | 515.7804942 | 1137.425302 | 2.205250711 | 1.140942683 | 0.043081915 |
| ENSG00000158887 | MPZ | myelin protein zero | 16.22820369 | 35.85301831 | 2.209302951 | 1.143591262 | 0.025592176 |
| ENSG00000236871 | LINC00106 | long intergenic non-protein coding RNA 106 | 18.07554324 | 40.14354388 | 2.220876205 | 1.151128977 | 0.030185762 |
| ENSG00000260695 | RP11-513N24.1 |  | 19.05135805 | 42.41502919 | 2.226352005 | 1.154681713 | 0.047522725 |
| ENSG00000166959 | MS4A8 | membrane-spanning 4-domains, subfamily A, member 8 | 8.73138394 | 19.48844634 | 2.231999701 | 1.158336834 | 0.04104481 |
| ENSG00000235436 | DPY19L2P4 | DPY19L2 pseudogene 4 | 121.548024 | 273.5756212 | 2.25076157 | 1.170413236 | 0.006060082 |
| ENSG00000187726 | DNAJB13 | DnaJ (Hsp40) homolog, subfamily B, member 13 | 16.42207663 | 36.96455723 | 2.25090639 | 1.17050606 | 0.045892198 |
| ENSG00000239552 | HOXB-AS2 | HOXB cluster antisense RNA 2 | 111.1900199 | 250.4568269 | 2.252511756 | 1.171534635 | 0.020223407 |
| ENSG00000246174 | KCTD21-AS1 | KCTD21 antisense RNA 1 | 95.2773559 | 214.7840998 | 2.25430374 | 1.172681914 | 0.007353828 |
| ENSG00000139832 | RAB20 | RAB20, member RAS oncogene family | 31.16991627 | 70.66128141 | 2.266970524 | 1.180765633 | 0.043737484 |
| ENSG00000248201 |  |  | 36.1995857 | 82.11948565 | 2.268520041 | 1.181751405 | 0.0149349 |
| ENSG00000254226 | CTB-12O2.1 | uncharacterized LOC101927115 | 45.39916635 | 103.0889595 | 2.270723622 | 1.183152121 | 0.029106282 |
| ENSG00000122254 | HS3ST2 | heparan sulfate (glucosamine) 3-O-sulfotransferase 2 | 215.6866837 | 490.7076402 | 2.275094742 | 1.185926625 | 0.016308251 |
| ENSG00000249437 | NAIP | NLR family, apoptosis inhibitory protein | 672.8112856 | 1537.317093 | 2.284915734 | 1.192140961 | 0.011108829 |
| ENSG00000249835 | VCAN-AS1 | VCAN antisense RNA 1 | 23.61000427 | 54.49784722 | 2.308252324 | 1.206800939 | 0.026598043 |
| ENSG00000231997 |  |  | 26.54741889 | 61.50055618 | 2.316630345 | 1.212027858 | 0.024560787 |
| ENSG00000223945 | RP11-458I7.1 |  | 13.20056513 | 30.61257988 | 2.319035555 | 1.213524939 | 0.030252999 |
| ENSG00000127362 | TAS2R3 | taste receptor, type 2, member 3 | 21.30255388 | 50.01527249 | 2.34785335 | 1.231342299 | 0.025133509 |
| ENSG00000213721 | HMGN2P30 | high mobility group nucleosomal binding domain 2 pseudogene 30 | 12.29309187 | 29.02481604 | 2.361067202 | 1.239439105 | 0.029633347 |
| ENSG00000263146 | RP11-849I19.1 |  | 126.903035 | 300.5693381 | 2.368496058 | 1.243971271 | 0.003087398 |
| ENSG00000174948 | GPR149 | G protein-coupled receptor 149 | 39.00092039 | 92.46148576 | 2.370751378 | 1.245344375 | 0.041853308 |
| ENSG00000256463 | SALL3 | spalt-like transcription factor 3 | 386.8882034 | 917.8256032 | 2.372327704 | 1.246303312 | 0.015381693 |
| ENSG00000231473 | LINC00441 | long intergenic non-protein coding RNA 441 | 16.69046182 | 39.66022167 | 2.376220747 | 1.248668867 | 0.024412853 |
| ENSG00000075643 | MOCOS | molybdenum cofactor sulfurase | 39.69524799 | 94.73260394 | 2.386497345 | 1.254894731 | 0.01555328 |
| ENSG00000237672 | KRR1P1 | KRR1, small subunit (SSU) processome component, homolog (yeast) pseudogene 1 | 13.82134786 | 33.00679283 | 2.388102316 | 1.255864649 | 0.029311282 |
| ENSG00000204305 | AGER | advanced glycosylation end product-specific receptor | 99.50402354 | 237.6391317 | 2.388236407 | 1.255945654 | 0.004134302 |
| ENSG00000231702 | RP11-54O7.10 |  | 22.30597385 | 53.4282961 | 2.395246066 | 1.260173873 | 0.01810856 |
| ENSG00000225880 | LINC00115 | long intergenic non-protein coding RNA 115 | 45.60351944 | 109.3698082 | 2.398275605 | 1.26199746 | 0.013149466 |
| ENSG00000237916 | RP11-537E18.1 |  | 13.53296579 | 32.46738536 | 2.39913304 | 1.262513162 | 0.032077993 |
| ENSG00000255710 | RP11-667M19.9 |  | 10.63279023 | 25.51126968 | 2.399301514 | 1.262614469 | 0.033798705 |
| ENSG00000101438 | SLC32A1 | solute carrier family 32 (GABA vesicular transporter), member 1 | 1051.764405 | 2538.810606 | 2.413858649 | 1.271341197 | 0.030432441 |
| ENSG00000264109 | MIR4712 | microRNA 4712 | 55.67480859 | 136.8467414 | 2.45796519 | 1.297464484 | 0.006746515 |
| ENSG00000248832 |  |  | 37.67266196 | 92.63790189 | 2.459021929 | 1.298084601 | 0.038881711 |
| ENSG00000223508 | RPL23AP53 | ribosomal protein L23a pseudogene 53 | 63.49906127 | 156.6369623 | 2.466760282 | 1.302617523 | 0.008014409 |
| ENSG00000164161 | HHIP | hedgehog interacting protein | 726.5687387 | 1795.199956 | 2.470791626 | 1.304973346 | 0.025458176 |
| ENSG00000197921 | HES5 | hes family bHLH transcription factor 5 | 705.7961418 | 1750.357007 | 2.479975313 | 1.310325759 | 0.01510799 |
| ENSG00000237550 | RPL9P9 | ribosomal protein L9 pseudogene 9 | 2125.391381 | 5305.825879 | 2.496399452 | 1.3198488 | 0.005569897 |
| ENSG00000204588 | LINC01123 | long intergenic non-protein coding RNA 1123 | 9.349302805 | 23.41909669 | 2.504903005 | 1.32475474 | 0.049893522 |
| ENSG00000256552 | RP11-113C12.4 |  | 5.212521914 | 13.12231055 | 2.517459066 | 1.33196832 | 0.046572642 |
| ENSG00000172748 | ZNF596 | zinc finger protein 596 | 192.3833856 | 485.7431693 | 2.524870678 | 1.336209496 | 0.043167374 |
| ENSG00000140479 | PCSK6 | proprotein convertase subtilisin/kexin type 6 | 114.8525647 | 290.1450181 | 2.526238912 | 1.336991084 | 0.031456883 |
| ENSG00000225489 | RP11-390F4.3 |  | 7.371957639 | 18.96024304 | 2.571941398 | 1.362857771 | 0.034785575 |
| ENSG00000250917 | RP4-785G19.5 |  | 12.48701548 | 32.36620438 | 2.591988809 | 1.37405949 | 0.020075711 |
| ENSG00000230002 | ALMS1-IT1 | ALMS1 intronic transcript 1 | 12.32028206 | 31.97435762 | 2.595261817 | 1.375880089 | 0.027294119 |
| ENSG00000239265 | CLRN1-AS1 | CLRN1 antisense RNA 1 | 6.828500722 | 17.82643857 | 2.610593349 | 1.384377747 | 0.045456793 |
| ENSG00000128242 | GAL3ST1 | galactose-3-O-sulfotransferase 1 | 16.51556349 | 43.27871017 | 2.620480384 | 1.38983131 | 0.008090516 |
| ENSG00000159648 | TEPP | testis, prostate and placenta expressed | 25.94612286 | 68.11169845 | 2.625120478 | 1.392383636 | 0.029646088 |
| ENSG00000256064 | RP11-117L5.4 |  | 6.951273683 | 18.32724208 | 2.636530069 | 1.39864045 | 0.043027345 |
| ENSG00000116791 | CRYZ | crystallin, zeta (quinone reductase) | 415.9184037 | 1103.32662 | 2.652747774 | 1.407487509 | 0.023100333 |
| ENSG00000265519 | CTD-3157E16.1 |  | 6.837988029 | 18.23807385 | 2.667169608 | 1.41530957 | 0.025077249 |
| ENSG00000269227 | RP11-345P4.6 |  | 22.54293989 | 60.16215918 | 2.668780535 | 1.416180672 | 0.048218577 |
| ENSG00000267731 | RP11-147L13.8 |  | 35.15598824 | 93.84657223 | 2.669433485 | 1.416533602 | 0.024336214 |
| ENSG00000225425 |  |  | 6.062262587 | 16.19003326 | 2.670625534 | 1.4171777 | 0.045123366 |
| ENSG00000203462 | RP11-814E24.1 |  | 7.26097574 | 19.40818841 | 2.672944946 | 1.418430123 | 0.045204565 |
| ENSG00000175772 | LINC01106 | long intergenic non-protein coding RNA 1106 | 9.129014746 | 24.40320002 | 2.673147179 | 1.418539272 | 0.019616486 |
| ENSG00000196302 | RP11-497H16.5 |  | 13.49262209 | 36.27323541 | 2.688375557 | 1.426734692 | 0.010910807 |
| ENSG00000248668 | OXCT1-AS1 | OXCT1 antisense RNA 1 | 11.7654475 | 31.7759964 | 2.700789443 | 1.43338117 | 0.014026229 |
| ENSG00000214289 | RPL39P5 | ribosomal protein L39 pseudogene 5 | 8.296572586 | 22.53628408 | 2.716336638 | 1.441662285 | 0.022605019 |
| ENSG00000185985 | SLITRK2 | SLIT and NTRK-like family, member 2 | 1471.821744 | 4018.238583 | 2.730112257 | 1.448960273 | 0.001484301 |
| ENSG00000260470 | RP11-834C11.11 |  | 23.90477127 | 65.76620071 | 2.75117465 | 1.460047727 | 0.04217505 |
| ENSG00000244151 | RP11-148K1.12 |  | 6.334527869 | 17.59518603 | 2.77766337 | 1.473871767 | 0.045031087 |
| ENSG00000125804 | FAM182A | family with sequence similarity 182, member A | 51.67690913 | 143.6506436 | 2.779783969 | 1.474972768 | 0.030349519 |
| ENSG00000162623 | TYW3 | tRNA-yW synthesizing protein 3 homolog (S. cerevisiae) | 213.3532128 | 593.6760646 | 2.782597257 | 1.476432113 | 0.006447986 |
| ENSG00000093072 | CECR1 | cat eye syndrome chromosome region, candidate 1 | 288.4062241 | 802.6380504 | 2.783012236 | 1.476647251 | 0.016109782 |
| ENSG00000174680 | GRIK1-AS1 | GRIK1 antisense RNA 1 | 7.038541473 | 19.64040315 | 2.790408101 | 1.480476134 | 0.024712459 |
| ENSG00000105427 | CNFN | cornifelin | 6.447755741 | 18.04682027 | 2.798930511 | 1.48487567 | 0.041184948 |
| ENSG00000213394 | RPSAP46 | ribosomal protein SA pseudogene 46 | 6.868596882 | 19.29396481 | 2.80901109 | 1.49006232 | 0.027213676 |
| ENSG00000226101 | AC007461.2 |  | 5.403844277 | 15.24813556 | 2.821720016 | 1.496574844 | 0.048458494 |
| ENSG00000267480 | RP11-703I16.1 |  | 7.245874459 | 21.01526932 | 2.900308229 | 1.53620623 | 0.041161684 |
| ENSG00000254459 | RP11-91P24.7 |  | 5.014371629 | 14.68974379 | 2.92952834 | 1.550668407 | 0.046166971 |
| ENSG00000235010 | RP11-140A10.3 |  | 6.214917705 | 18.29554746 | 2.9438117 | 1.557685393 | 0.040575542 |
| ENSG00000253047 | SNORA40 | Small nucleolar RNA SNORA40 | 4.960561119 | 14.64397651 | 2.952080654 | 1.561732138 | 0.029879225 |
| ENSG00000237945 | LINC00649 | long intergenic non-protein coding RNA 649 | 120.6530652 | 356.5831337 | 2.955441979 | 1.563373898 | 0.002805001 |
| ENSG00000204805 | FAM27E4 | family with sequence similarity 27, member E4 | 25.18151957 | 74.60344896 | 2.962626967 | 1.566876984 | 0.004106621 |
| ENSG00000095777 | MYO3A | myosin IIIA | 79.88364009 | 237.4279714 | 2.972172665 | 1.57151793 | 0.010956035 |
| ENSG00000230922 | RP4-753M9.1 |  | 5.088989676 | 15.13002976 | 2.97309107 | 1.571963655 | 0.030522433 |
| ENSG00000255753 | RP11-22B23.2 |  | 6.364665891 | 19.05961597 | 2.994598035 | 1.582362363 | 0.016075166 |
| ENSG00000260035 | CTD-2651B20.6 |  | 4.127450045 | 12.47211908 | 3.021749251 | 1.595383949 | 0.046470091 |
| ENSG00000250770 | RP5-1063M23.1 |  | 6.901285845 | 20.93730412 | 3.033826534 | 1.601138599 | 0.017761256 |
| ENSG00000232855 | AF131217.1 |  | 4.665498552 | 14.16538811 | 3.036200301 | 1.60226697 | 0.041906265 |
| ENSG00000183837 | PNMA3 | paraneoplastic Ma antigen 3 | 292.0139902 | 887.3689496 | 3.038789165 | 1.603496582 | 0.021483558 |
| ENSG00000179855 | GIPC3 | GIPC PDZ domain containing family, member 3 | 4.748687444 | 14.56325833 | 3.066796563 | 1.616732468 | 0.024674319 |
| ENSG00000264290 | RP11-68I3.4 |  | 5.064202282 | 15.55110675 | 3.070790993 | 1.618610322 | 0.026182239 |
| ENSG00000204791 | CTD-3065J16.6 |  | 17.05988657 | 52.91223383 | 3.10155836 | 1.632993272 | 0.002403442 |
| ENSG00000229465 | ACTG1P11 | actin gamma 1 pseudogene 11 | 8.306572345 | 25.9130335 | 3.119581992 | 1.641352728 | 0.012067221 |
| ENSG00000266176 | RP11-855A2.5 |  | 4.183605569 | 13.07774799 | 3.125951473 | 1.644295382 | 0.04008348 |
| ENSG00000262877 | RP11-1055B8.4 |  | 190.3840135 | 596.1828972 | 3.131475623 | 1.646842648 | 0.030988006 |
| ENSG00000237198 |  |  | 33.90719086 | 108.5555194 | 3.2015486 | 1.678769911 | 0.001127604 |
| ENSG00000177133 | LINC00982 | long intergenic non-protein coding RNA 982 | 93.24679245 | 299.8774958 | 3.215955079 | 1.685247255 | 0.022734984 |
| ENSG00000248174 | RP11-148L24.1 |  | 7.028137704 | 22.86231719 | 3.252969442 | 1.70175727 | 0.0391048 |
| ENSG00000154646 | TMPRSS15 | transmembrane protease, serine 15 | 31.08402106 | 101.3436114 | 3.260312145 | 1.705010096 | 0.012984892 |
| ENSG00000269311 |  |  | 4.780872995 | 15.63456441 | 3.270232116 | 1.709393039 | 0.02999727 |
| ENSG00000182368 |  |  | 9.441475591 | 31.28293919 | 3.313352759 | 1.728291809 | 0.007575185 |
| ENSG00000120903 | CHRNA2 | cholinergic receptor, nicotinic, alpha 2 (neuronal) | 42.60749584 | 142.0610853 | 3.334180583 | 1.737332244 | 0.032042367 |
| ENSG00000146722 | AC006014.8 |  | 3.404817095 | 11.37501095 | 3.340858151 | 1.740218729 | 0.038975253 |
| ENSG00000108439 | PNPO | pyridoxamine 5'-phosphate oxidase | 72.5876134 | 243.0551475 | 3.348438338 | 1.743488401 | 0.007865248 |
| ENSG00000241484 | ARHGAP8 | Rho GTPase activating protein 8 | 17.72922147 | 59.95131559 | 3.38149736 | 1.757662227 | 0.01040525 |
| ENSG00000207234 | RNU6-125P | RNA, U6 small nuclear 125, pseudogene | 3.805353748 | 12.88381012 | 3.385706289 | 1.759456825 | 0.016864618 |
| ENSG00000261212 | RP3-507I15.2 |  | 8.652431131 | 29.59477685 | 3.420400164 | 1.774165121 | 0.0038107 |
| ENSG00000231688 | RPL21P43 | ribosomal protein L21 pseudogene 43 | 2.510141725 | 8.682362166 | 3.458913128 | 1.79031878 | 0.041152721 |
| ENSG00000127903 | ZNF835 | zinc finger protein 835 | 98.06923514 | 340.3939369 | 3.470955355 | 1.795332809 | 0.006025298 |
| ENSG00000252755 | RNU6-703P | RNA, U6 small nuclear 703, pseudogene | 3.323361724 | 11.68215522 | 3.515162112 | 1.813591225 | 0.024835171 |
| ENSG00000253764 | RP11-439C15.4 |  | 34.35005988 | 123.3346392 | 3.59052181 | 1.844193526 | 0.038451134 |
| ENSG00000142698 | C1orf94 | chromosome 1 open reading frame 94 | 11.71750719 | 42.18422534 | 3.600102365 | 1.848037928 | 0.023160185 |
| ENSG00000146858 | ZC3HAV1L | zinc finger CCCH-type, antiviral 1-like | 45.74239111 | 165.6387722 | 3.621121857 | 1.856436727 | 0.014978697 |
| ENSG00000196092 | PAX5 | paired box 5 | 246.3646723 | 905.8384484 | 3.67681957 | 1.878458382 | 0.037311443 |
| ENSG00000249834 | PGBD4P3 | piggyBac transposable element derived 4 pseudogene 3 | 3.900020056 | 14.35098137 | 3.679719889 | 1.879595948 | 0.012831887 |
| ENSG00000228663 | PSMD10P1 | proteasome 26S subunit, non-ATPase, 10 pseudogene 1 | 3.824229321 | 14.12889134 | 3.694572202 | 1.885407323 | 0.021488976 |
| ENSG00000187144 | SPATA21 | spermatogenesis associated 21 | 2.057528083 | 7.685446886 | 3.735281646 | 1.901217029 | 0.049330659 |
| ENSG00000237917 | PARP4P1 | poly (ADP-ribose) polymerase family, member 4 pseudogene 1 | 4.379923292 | 16.57643192 | 3.784639778 | 1.920155991 | 0.009863625 |
| ENSG00000232894 | MRPS31P2 | mitochondrial ribosomal protein S31 pseudogene 2 | 2.226812439 | 8.466119985 | 3.801900797 | 1.926720888 | 0.040077244 |
| ENSG00000116157 | GPX7 | glutathione peroxidase 7 | 26.79616851 | 106.6277495 | 3.979216263 | 1.992484309 | 0.033548321 |
| ENSG00000262703 | RP11-485G7.6 |  | 2.094048758 | 8.419625605 | 4.020740001 | 2.007461048 | 0.047543592 |
| ENSG00000189398 | OR7E12P | olfactory receptor, family 7, subfamily E, member 12 pseudogene | 6.903531819 | 28.25875364 | 4.093376315 | 2.033291304 | 0.002560858 |
| ENSG00000259595 | RP11-516C1.1 |  | 4.182432881 | 17.15587186 | 4.101888148 | 2.036288152 | 0.004969869 |
| ENSG00000222306 |  |  | 2.792810776 | 11.45937248 | 4.103168243 | 2.03673831 | 0.014054407 |
| ENSG00000233670 | PIRT | phosphoinositide-interacting regulator of transient receptor potential channels | 12.41055583 | 50.92924911 | 4.103704122 | 2.036926716 | 0.049432583 |
| ENSG00000231459 | LINC00032 | long intergenic non-protein coding RNA 32 | 4.309127916 | 18.03789324 | 4.18597303 | 2.065563017 | 0.029448607 |
| ENSG00000171540 | OTP | orthopedia homeobox | 463.0035178 | 1943.290604 | 4.197140041 | 2.069406601 | 0.031613775 |
| ENSG00000164530 | PI16 | peptidase inhibitor 16 | 163.5947402 | 690.8960239 | 4.223216608 | 2.078342245 | 0.022816384 |
| ENSG00000215796 | RP11-551G24.2 |  | 1.663976537 | 7.269601962 | 4.368812781 | 2.127241283 | 0.038115648 |
| ENSG00000225009 |  |  | 3.705692442 | 16.25343003 | 4.386070967 | 2.132929155 | 0.043391057 |
| ENSG00000136698 | CFC1 | cripto, FRL-1, cryptic family 1 | 24.43336313 | 108.4908796 | 4.440276152 | 2.150649404 | 0.01189628 |
| ENSG00000198734 | F5 | coagulation factor V (proaccelerin, labile factor) | 117.2226954 | 521.4897804 | 4.448710025 | 2.153387064 | 0.042939907 |
| ENSG00000266471 |  |  | 2.707632137 | 12.1711749 | 4.495136076 | 2.16836479 | 0.0119913 |
| ENSG00000214688 | C10orf105 | chromosome 10 open reading frame 105 | 27.34722202 | 123.1382899 | 4.502771424 | 2.170813244 | 0.00012558 |
| ENSG00000259255 | RP11-627D16.1 |  | 2.896608761 | 13.09331027 | 4.520220488 | 2.176393146 | 0.026767527 |
| ENSG00000232833 |  |  | 9.037107226 | 41.08796645 | 4.546583925 | 2.184782984 | 0.000384257 |
| ENSG00000243970 | PPIEL | peptidylprolyl isomerase E-like pseudogene | 13.18884729 | 60.13365314 | 4.559432059 | 2.188854128 | 0.021783573 |
| ENSG00000129514 | FOXA1 | forkhead box A1 | 270.5854357 | 1242.550722 | 4.592082786 | 2.199148651 | 0.037308714 |
| ENSG00000223642 | AC008277.1 |  | 2.549568609 | 11.7437633 | 4.606176611 | 2.203569728 | 0.006927376 |
| ENSG00000227394 | AC007386.3 |  | 4.345549188 | 20.11964645 | 4.629943325 | 2.210994533 | 0.002930446 |
| ENSG00000231258 | ZSWIM5P2 | zinc finger, SWIM-type containing 5 pseudogene 2 | 1.51631714 | 7.031127478 | 4.63697685 | 2.213184524 | 0.038699263 |
| ENSG00000199545 | RNA5SP195 | RNA, 5S ribosomal pseudogene 195 | 1.630618298 | 7.5900021 | 4.654677376 | 2.218681175 | 0.031429379 |
| ENSG00000183242 | WT1-AS | WT1 antisense RNA | 5.33457722 | 24.96574214 | 4.679985145 | 2.22650395 | 0.049666322 |
| ENSG00000258815 | RP11-408B11.2 |  | 11.71572456 | 54.94888914 | 4.69018274 | 2.229644134 | 0.048759941 |
| ENSG00000259485 | CTD-2147F2.1 |  | 8.0959201 | 38.16533428 | 4.714144138 | 2.23699587 | 0.012208334 |
| ENSG00000125618 | PAX8 | paired box 8 | 82.65998072 | 392.7873409 | 4.75184409 | 2.248487502 | 0.006062569 |
| ENSG00000222493 |  |  | 0.959549884 | 4.570150351 | 4.762806425 | 2.251811914 | 0.049599969 |
| ENSG00000202515 | VTRNA1-3 | vault RNA 1-3 | 1.707738545 | 8.255209436 | 4.834000766 | 2.273217702 | 0.029436538 |
| ENSG00000058866 | DGKG | diacylglycerol kinase, gamma 90kDa | 76.10909957 | 369.8420973 | 4.859367663 | 2.280768592 | 0.002459868 |
| ENSG00000213328 | RP11-281O15.2 |  | 2.290110255 | 11.3150783 | 4.940844342 | 2.304757605 | 0.025612503 |
| ENSG00000256564 | RP11-424M22.3 |  | 7.261635976 | 36.10529137 | 4.972060221 | 2.31384377 | 0.014131071 |
| ENSG00000196690 |  |  | 2.68483449 | 13.39672122 | 4.989775448 | 2.318974892 | 0.045483262 |
| ENSG00000264581 |  |  | 1.309595648 | 6.537885233 | 4.992293034 | 2.319702619 | 0.041101386 |
| ENSG00000250138 | RP11-848G14.5 |  | 5.375894447 | 27.57291089 | 5.128990378 | 2.358674865 | 0.03736326 |
| ENSG00000258099 | RP11-686G8.2 |  | 1.556916713 | 8.050425251 | 5.170748817 | 2.370373224 | 0.019971167 |
| ENSG00000167711 | SERPINF2 | serpin peptidase inhibitor, clade F (alpha-2 antiplasmin, pigment epithelium derived factor), member 2 | 20.02284039 | 103.7502581 | 5.181595421 | 2.373396374 | 0.010821316 |
| ENSG00000232608 | TIMM9P2 | TIMM9 pseudogene 2 | 1.770780135 | 9.223264186 | 5.208588014 | 2.380892328 | 0.014434127 |
| ENSG00000109851 | DBX1 | developing brain homeobox 1 | 78.39730293 | 415.3060705 | 5.297453547 | 2.405299032 | 0.026789473 |
| ENSG00000259797 | RP11-96D1.3 |  | 1.239056499 | 6.617599401 | 5.34083749 | 2.417065987 | 0.041092884 |
| ENSG00000251158 | RP11-98J23.1 |  | 17.01373009 | 91.9974271 | 5.407246185 | 2.434894042 | 0.037248719 |
| ENSG00000215156 | RP11-1023L17.2 |  | 11.29992752 | 61.47570497 | 5.440362767 | 2.443702854 | 0.004562092 |
| ENSG00000231838 | RP11-449H15.2 |  | 3.502389612 | 19.44154795 | 5.550938102 | 2.472731606 | 0.0139999 |
| ENSG00000140968 | IRF8 | interferon regulatory factor 8 | 6.359109699 | 35.5393034 | 5.588723121 | 2.482518702 | 0.042360514 |
| ENSG00000231527 | RP11-374M1.2 |  | 4.975861207 | 27.83819153 | 5.594647916 | 2.484047342 | 0.000773135 |
| ENSG00000230068 | CDC42-IT1 | CDC42 intronic transcript 1 | 1.134242648 | 6.450030141 | 5.686640466 | 2.507576594 | 0.035295777 |
| ENSG00000225353 |  |  | 8.296572947 | 47.95828702 | 5.780493624 | 2.531192697 | 2.92560672071043e-05 |
| ENSG00000221870 | TMEM257 | transmembrane protein 257 | 26.40759388 | 152.9742378 | 5.79281242 | 2.534263949 | 0.000109933 |
| ENSG00000160180 | TFF3 | trefoil factor 3 (intestinal) | 53.19090635 | 314.8659899 | 5.919545492 | 2.565486409 | 0.004750455 |
| ENSG00000184937 | WT1 | Wilms tumor 1 | 16.98180148 | 103.2675713 | 6.081072814 | 2.604325864 | 0.012496026 |
| ENSG00000233974 | RP11-823P9.3 |  | 7.42918681 | 45.57161512 | 6.134132347 | 2.616859294 | 0.011739607 |
| ENSG00000236485 | RP1-156L9.1 |  | 0.781034448 | 4.800235014 | 6.145996539 | 2.619646955 | 0.048461971 |
| ENSG00000139865 | TTC6 | tetratricopeptide repeat domain 6 | 29.33041822 | 180.7568699 | 6.16277847 | 2.623580932 | 0.001212996 |
| ENSG00000197284 |  |  | 3.685240172 | 22.75499586 | 6.174630363 | 2.626352774 | 0.029287243 |
| ENSG00000181965 | NEUROG1 | neurogenin 1 | 90.23638257 | 560.3065891 | 6.209320156 | 2.63443532 | 0.032582824 |
| ENSG00000233265 | MICF | MHC class I polypeptide-related sequence F (pseudogene) | 1.378145051 | 8.605256393 | 6.244086128 | 2.642490437 | 0.01099829 |
| ENSG00000232818 | RPS2P32 | ribosomal protein S2 pseudogene 32 | 4.059817105 | 25.43101537 | 6.264079075 | 2.647102425 | 0.003529206 |
| ENSG00000180221 | TPT1P10 | tumor protein, translationally-controlled 1 pseudogene 10 | 0.639699922 | 4.116980786 | 6.43580004 | 2.686119502 | 0.044861096 |
| ENSG00000127412 | TRPV5 | transient receptor potential cation channel, subfamily V, member 5 | 1.162192478 | 7.501558788 | 6.45466128 | 2.69034139 | 0.025836413 |
| ENSG00000233022 |  |  | 0.777872012 | 5.391388391 | 6.930945331 | 2.793052139 | 0.041818062 |
| ENSG00000186481 | ANKRD20A5P | ankyrin repeat domain 20 family, member A5, pseudogene | 4.154226824 | 28.85460781 | 6.94584312 | 2.796149826 | 0.00098959 |
| ENSG00000165409 | TSHR | thyroid stimulating hormone receptor | 33.02674385 | 229.413534 | 6.946295859 | 2.79624386 | 0.000213845 |
| ENSG00000106038 | EVX1 | even-skipped homeobox 1 | 9.741889147 | 68.24175424 | 7.004981601 | 2.808381261 | 0.023922803 |
| ENSG00000248528 | CTC-458G6.2 |  | 0.812146713 | 5.696615936 | 7.014269519 | 2.810292866 | 0.028999664 |
| ENSG00000214263 | RPSAP53 | ribosomal protein SA pseudogene 53 | 32.81778242 | 232.48972 | 7.08426051 | 2.824617265 | 0.011925405 |
| ENSG00000204807 |  |  | 1.310768337 | 9.418655109 | 7.185598587 | 2.845108344 | 0.007012146 |
| ENSG00000265787 | CYP4F35P | cytochrome P450, family 4, subfamily F, polypeptide 35, pseudogene | 0.569160773 | 4.242525216 | 7.454001431 | 2.898015096 | 0.049280806 |
| ENSG00000231827 | RP11-216N14.5 |  | 21.28714571 | 159.327464 | 7.484679541 | 2.903940548 | 0.022348058 |
| ENSG00000212396 | RNA5SP323 | RNA, 5S ribosomal pseudogene 323 | 0.569160773 | 4.38963451 | 7.71246846 | 2.947192684 | 0.035440332 |
| ENSG00000165970 | SLC6A5 | solute carrier family 6 (neurotransmitter transporter), member 5 | 535.566389 | 4223.615076 | 7.886258665 | 2.979341031 | 0.014373032 |
| ENSG00000149021 | SCGB1A1 | secretoglobin, family 1A, member 1 (uteroglobin) | 9.234745059 | 74.39731518 | 8.056239204 | 3.01010652 | 0.021913255 |
| ENSG00000249267 | LINC00939 | long intergenic non-protein coding RNA 939 | 1.096805511 | 9.010579306 | 8.215293608 | 3.038312137 | 0.040387661 |
| ENSG00000081041 | CXCL2 | chemokine (C-X-C motif) ligand 2 | 1.480052692 | 12.53802241 | 8.471335161 | 3.08258937 | 0.021717388 |
| ENSG00000134330 | IAH1 | isoamyl acetate-hydrolyzing esterase 1 homolog | 35.35602248 | 307.3337 | 8.692541707 | 3.119778084 | 0.01290254 |
| ENSG00000232358 | RP5-955M13.4 |  | 0.916044102 | 7.978444207 | 8.709672591 | 3.122618487 | 0.007999914 |
| ENSG00000152093 | CFC1B | cripto, FRL-1, cryptic family 1B | 9.566072074 | 85.80836374 | 8.970072886 | 3.165119708 | 7.30207897893805e-05 |
| ENSG00000253839 | RP11-431D12.1 |  | 1.275320947 | 11.52893087 | 9.040023136 | 3.176326465 | 0.017748849 |
| ENSG00000125144 | MT1G | metallothionein 1G | 0.884931836 | 8.009067181 | 9.050490503 | 3.177995983 | 0.043830348 |
| ENSG00000266853 | ITM2BP1 | integral membrane protein 2B pseudogene 1 | 0.498621623 | 4.755753585 | 9.537800531 | 3.253656611 | 0.033921781 |
| ENSG00000216863 | LY86-AS1 | LY86 antisense RNA 1 | 0.492296752 | 4.711209748 | 9.569857465 | 3.258497437 | 0.034437947 |
| ENSG00000182489 | XKRX | XK, Kell blood group complex subunit-related, X-linked | 2.267155785 | 23.14755061 | 10.2099515 | 3.351904108 | 0.002666228 |
| ENSG00000237810 | CTD-2571E19.3 |  | 0.458022051 | 4.697968977 | 10.25708035 | 3.358548225 | 0.027809746 |
| ENSG00000181234 | TMEM132C | transmembrane protein 132C | 279.4478845 | 2876.372345 | 10.29305464 | 3.363599285 | 0.000403052 |
| ENSG00000207195 | Y_RNA | Y RNA | 0.495459188 | 5.127242198 | 10.34846527 | 3.37134492 | 0.01912514 |
| ENSG00000226292 | RP11-7G12.1 |  | 0.884931836 | 9.224636215 | 10.42412063 | 3.38185378 | 0.003378492 |
| ENSG00000101460 | MAP1LC3A | microtubule-associated protein 1 light chain 3 alpha | 22.15723358 | 232.2665267 | 10.4826501 | 3.389931583 | 0.011453076 |
| ENSG00000171517 | LPAR3 | lysophosphatidic acid receptor 3 | 8.784633979 | 95.15179397 | 10.83161737 | 3.437176776 | 0.002624963 |
| ENSG00000247516 | MIR4458HG | MIR4458 host gene | 15.16245411 | 173.0237163 | 11.41132663 | 3.512394617 | 4.44717246095538e-11 |
| ENSG00000232073 | RP1-302D9.3 |  | 0.458022051 | 5.755391636 | 12.56575229 | 3.651425141 | 0.010108349 |
| ENSG00000206356 | RP11-93O17.2 |  | 0.498621623 | 6.289750357 | 12.61427516 | 3.656985403 | 0.024445198 |
| ENSG00000239087 |  |  | 0.319849961 | 4.378604188 | 13.68955673 | 3.775003827 | 0.016983686 |
| ENSG00000213068 | RP11-277B15.1 |  | 0.246148376 | 3.585067816 | 14.56466166 | 3.864400283 | 0.04369007 |
| ENSG00000235548 | AC073551.1 |  | 0.246148376 | 3.595385102 | 14.60657657 | 3.868546179 | 0.038567145 |
| ENSG00000259402 | RP11-30K9.4 |  | 0.246148376 | 3.672873991 | 14.92138217 | 3.899309274 | 0.047935026 |
| ENSG00000207457 | RNU6-476P | RNA, U6 small nuclear 476, pseudogene | 0.458022051 | 6.87194063 | 15.00351482 | 3.90722861 | 0.004051511 |
| ENSG00000253678 | RP11-981G7.3 |  | 0.498621623 | 7.520278244 | 15.08213421 | 3.914768688 | 0.004844173 |
| ENSG00000240184 | PCDHGC3 | protocadherin gamma subfamily C, 3 | 56.32550255 | 892.2043157 | 15.84014834 | 3.985513941 | 2.07730403523971e-11 |
| ENSG00000233783 | AP001442.2 |  | 0.246148376 | 3.907353911 | 15.87397802 | 3.988591808 | 0.03404008 |
| ENSG00000251532 | CTD-2245E15.3 |  | 0.211873675 | 3.632640498 | 17.14531312 | 4.099742347 | 0.043343734 |
| ENSG00000157211 | CDCP2 | CUB domain containing protein 2 | 0.246148376 | 4.288400038 | 17.42201231 | 4.122839365 | 0.018071215 |
| ENSG00000169436 | COL22A1 | collagen, type XXII, alpha 1 | 34.57390112 | 609.1195691 | 17.61790106 | 4.138970151 | 0.007876202 |
| ENSG00000243350 | RP11-379F12.3 |  | 0.211873675 | 3.827203623 | 18.0636109 | 4.17501441 | 0.035499161 |
| ENSG00000250543 | RP11-442N1.1 |  | 0.246148376 | 4.52710118 | 18.39175725 | 4.200987424 | 0.049212222 |
| ENSG00000205565 |  |  | 1.555744024 | 31.13361043 | 20.01203922 | 4.32279628 | 0.023455246 |
| ENSG00000088320 | REM1 | RAS (RAD and GEM)-like GTP-binding 1 | 0.319849961 | 6.660861739 | 20.82495716 | 4.380241623 | 0.01161569 |
| ENSG00000255160 | RP11-428C19.5 |  | 0.211873675 | 4.490835734 | 21.19581744 | 4.405707702 | 0.016805373 |
| ENSG00000253308 | RP1-170O19.17 |  | 1.276493636 | 28.37425239 | 22.22827564 | 4.47432413 | 0.014439143 |
| ENSG00000138161 | CUZD1 | CUB and zona pellucida-like domains 1 | 1.343870349 | 31.22806837 | 23.23741154 | 4.538377468 | 2.6703101033415e-05 |
| ENSG00000134463 | ECHDC3 | enoyl CoA hydratase domain containing 3 | 0.531723636 | 12.88395774 | 24.23055299 | 4.598755426 | 0.018005827 |
| ENSG00000256417 | RP11-1038A11.3 |  | 2.165760596 | 75.78571164 | 34.99265421 | 5.128980193 | 0.044310552 |
| ENSG00000255366 | RP11-1134I14.8 |  | 3.938374017 | 141.9883115 | 36.05252089 | 5.172028235 | 0.001344434 |
| ENSG00000204556 | CTD-2514C3.1 |  | 1.063447272 | 39.74561313 | 37.37431481 | 5.223975225 | 6.20349902118999e-07 |
| ENSG00000254348 | RP11-1134I14.4 |  | 0.492296752 | 19.85600575 | 40.33340797 | 5.333903406 | 0.00074711 |
| ENSG00000258702 | RP11-433J8.1 |  | 0.458022051 | 21.77481055 | 47.54096556 | 5.5710993 | 0.000192779 |
| ENSG00000231081 | RP4-760C5.3 |  | 0.211873675 | 13.55135188 | 63.95958293 | 5.999088626 | 4.52750191636109e-06 |
| ENSG00000175868 | CALCB | calcitonin-related polypeptide beta | 0.635621025 | 44.51751355 | 70.03782414 | 6.130062359 | 0.002391262 |
| ENSG00000053438 | NNAT | neuronatin | 257.4962239 | 36651.90372 | 142.3395775 | 7.153193049 | 0.019222626 |
| ENSG00000147724 | FAM135B | family with sequence similarity 135, member B | 5.355945952 | 1019.295681 | 190.3110468 | 7.572215497 | 2.51751161397466e-09 |
| ENSG00000174080 | CTSF | cathepsin F | 1.304443465 | 561.2440446 | 430.255553 | 8.749050002 | 0.001514888 |
| ENSG00000151365 | THRSP | thyroid hormone responsive | 0 | 2.828603402 | inf | inf | 0.036240234 |
| ENSG00000200428 | Y_RNA | Y RNA | 0 | 2.620864663 | inf | inf | 0.046449593 |
| ENSG00000223408 |  |  | 0 | 2.950839892 | inf | inf | 0.02970109 |
| ENSG00000223484 | TRPC6P | transient receptor potential cation channel, subfamily C, member 6 pseudogene | 0 | 3.463679452 | inf | inf | 0.015218044 |
| ENSG00000223634 | AC012506.3 |  | 0 | 3.215357049 | inf | inf | 0.02576738 |
| ENSG00000228467 | RP11-402N8.1 |  | 0 | 4.882859176 | inf | inf | 0.003863814 |
| ENSG00000250677 | RP11-576N17.2 |  | 0 | 3.81025013 | inf | inf | 0.01452691 |
| ENSG00000251957 | RNU6-1095P | RNA, U6 small nuclear 1095, pseudogene | 0 | 3.320734097 | inf | inf | 0.021302157 |

**Supplementary Table S8.** DEG genes (fold-change ≥ 2, *P*-value < 0.01) found in iPSC-derived neurons from AN compared to unaffected controls. Validated genes (qRT-PCR) where selected from this list and are highlighted in grey.

| **Ensembl ID** | **Symbol** | **Description** | **Expression in control** | **Expression in AN** | **Fold-Change** | **Log2 (Fold-Change)** | ***P*-value** |
| --- | --- | --- | --- | --- | --- | --- | --- |
| ENSG00000260409 | RP11-403B2.7 |  | 30.24548953 | 0.189548881 | 0.006267013 | -7.318006263 | 0.003677783 |
| ENSG00000249780 | RP11-352E6.2 |  | 60.12390041 | 0.460145093 | 0.007653281 | -7.029705949 | 0.003511925 |
| ENSG00000240661 | RP11-174O3.3 |  | 33.26027299 | 0.273720714 | 0.008229659 | -6.924951583 | 0.002783834 |
| ENSG00000247765 | RP11-32B5.7 |  | 27.80904939 | 0.349122932 | 0.012554292 | -6.315675494 | 0.007127375 |
| ENSG00000251521 | IMPA1P | inositol(myo)-1(or 4)-monophosphatase 1 pseudogene | 110.3172462 | 1.646887312 | 0.014928648 | -6.065772696 | 0.00279095 |
| ENSG00000197416 | FABP12 | fatty acid binding protein 12 | 19.70587237 | 0.349122932 | 0.017716695 | -5.818746683 | 0.009123694 |
| ENSG00000227496 | RP11-145A3.1 |  | 12.38934356 | 0.574678959 | 0.04638494 | -4.43019971 | 0.003112029 |
| ENSG00000234665 | RP11-262H14.3 |  | 40.34466562 | 3.996768559 | 0.099065601 | -3.335471995 | 0.008778453 |
| ENSG00000231429 | RP11-343N15.2 |  | 8.990339244 | 1.142211872 | 0.127048807 | -2.97654527 | 0.003511457 |
| ENSG00000163239 | TDRD10 | tudor domain containing 10 | 21.41486565 | 2.796522145 | 0.130587891 | -2.936906966 | 0.00021248 |
| ENSG00000232560 | LINC01549 | long intergenic non-protein coding RNA 1549 | 12.31446061 | 1.858151246 | 0.15089181 | -2.728413596 | 0.002952257 |
| ENSG00000152936 | LMNTD1 | lamin tail domain containing 1 | 22.92945722 | 3.799083681 | 0.165685722 | -2.593478809 | 0.004182218 |
| ENSG00000184459 | BPIFC | BPI fold containing family C | 9.408578781 | 1.642865767 | 0.174613595 | -2.517762205 | 0.008810324 |
| ENSG00000171227 | TMEM37 | transmembrane protein 37 | 44.86469261 | 8.084558894 | 0.180198691 | -2.472339561 | 0.009763147 |
| ENSG00000148702 | HABP2 | hyaluronan binding protein 2 | 19.67066349 | 3.632935831 | 0.184688017 | -2.436837832 | 0.000278269 |
| ENSG00000223489 | NEFHP1 | neurofilament, heavy polypeptide pseudogene 1 | 14.76340234 | 2.819570362 | 0.190983778 | -2.388477989 | 0.003207999 |
| ENSG00000236090 | LDHAP3 | lactate dehydrogenase A pseudogene 3 | 64.28398496 | 13.49022532 | 0.209853595 | -2.25254492 | 0.000358855 |
| ENSG00000118523 | CTGF | connective tissue growth factor | 7619.724875 | 1640.699927 | 0.215322725 | -2.215427504 | 0.004610977 |
| ENSG00000237512 | UNC5B-AS1 | UNC5B antisense RNA 1 | 12.10290058 | 2.608319918 | 0.215511968 | -2.214160108 | 0.005719645 |
| ENSG00000006128 | TAC1 | tachykinin, precursor 1 | 9908.007864 | 2494.84013 | 0.251800378 | -1.989647643 | 0.002477374 |
| ENSG00000134007 | ADAM20 | ADAM metallopeptidase domain 20 | 20.45884023 | 5.476658285 | 0.267691532 | -1.901356592 | 0.007435009 |
| ENSG00000254768 | CTD-2140G10.2 |  | 194.463804 | 52.46706336 | 0.269803749 | -1.8900177 | 0.001032923 |
| ENSG00000141934 | PLPP2 | phospholipid phosphatase 2 | 69.17356313 | 18.97167188 | 0.274261886 | -1.86637395 | 0.000694695 |
| ENSG00000091664 | SLC17A6 | solute carrier family 17 (vesicular glutamate transporter), member 6 | 7836.69017 | 2208.499828 | 0.281815381 | -1.82717774 | 0.007710461 |
| ENSG00000259378 | DCAF13P3 | DDB1 and CUL4 associated factor 13 pseudogene 3 | 26.64009436 | 7.560427965 | 0.283798843 | -1.817059386 | 0.003093099 |
| ENSG00000250331 | LINC01340 | long intergenic non-protein coding RNA 1340 | 19.16290982 | 5.643515082 | 0.29450199 | -1.763650711 | 0.007705855 |
| ENSG00000269067 | ZNF728 | zinc finger protein 728 | 160.0355716 | 48.09215019 | 0.300509129 | -1.734519278 | 0.00379116 |
| ENSG00000255372 | CTD-3064C13.1 |  | 56.29152182 | 17.16985788 | 0.305016765 | -1.713039553 | 0.002753354 |
| ENSG00000118785 | SPP1 | secreted phosphoprotein 1 | 1955.722104 | 619.5140756 | 0.316769992 | -1.65849242 | 0.004696785 |
| ENSG00000128564 | VGF | VGF nerve growth factor inducible | 1107.710188 | 380.1864169 | 0.343218308 | -1.542801582 | 0.000204313 |
| ENSG00000188993 | LRRC66 | leucine rich repeat containing 66 | 82.15148204 | 28.33095575 | 0.344862382 | -1.53590733 | 0.001717247 |
| ENSG00000197355 | UAP1L1 | UDP-N-acetylglucosamine pyrophosphorylase 1 like 1 | 954.8628586 | 331.0179697 | 0.346665457 | -1.528384005 | 0.001131664 |
| ENSG00000264015 | RP11-176N18.2 |  | 43.74726101 | 15.28192656 | 0.349323048 | -1.517366261 | 0.005605221 |
| ENSG00000144115 | THNSL2 | threonine synthase-like 2 | 763.0640544 | 267.2822894 | 0.350275036 | -1.513439922 | 0.00540395 |
| ENSG00000126861 | OMG | oligodendrocyte myelin glycoprotein | 613.0083817 | 215.2619086 | 0.35115655 | -1.509813748 | 0.0011905 |
| ENSG00000242349 | NPPA-AS1 | NPPA antisense RNA 1 | 24.3973707 | 8.796261073 | 0.360541354 | -1.471763348 | 0.008123616 |
| ENSG00000112812 | PRSS16 | protease, serine, 16 (thymus) | 115.4458424 | 42.32724727 | 0.366641591 | -1.447557646 | 0.001492773 |
| ENSG00000260910 | LINC00565 | long intergenic non-protein coding RNA 565 | 85.05190552 | 32.90085 | 0.386832603 | -1.370218702 | 0.003118473 |
| ENSG00000075073 | TACR2 | tachykinin receptor 2 | 46.06328973 | 17.8640444 | 0.387815211 | -1.366558706 | 0.007886936 |
| ENSG00000235448 | LURAP1L-AS1 | LURAP1L antisense RNA 1 | 53.39658844 | 20.99657808 | 0.393219468 | -1.346593346 | 0.007362522 |
| ENSG00000205663 | RP11-706O15.5 |  | 2002.67617 | 794.4534263 | 0.3966959 | -1.333894607 | 0.00192684 |
| ENSG00000139278 | GLIPR1 | GLI pathogenesis-related 1 | 782.7826077 | 312.9929747 | 0.399846613 | -1.322481426 | 0.007195258 |
| ENSG00000081052 | COL4A4 | collagen, type IV, alpha 4 | 67.65009055 | 27.12863445 | 0.401014015 | -1.318275436 | 0.007541179 |
| ENSG00000156466 | GDF6 | growth differentiation factor 6 | 145.3810642 | 58.91981275 | 0.405278453 | -1.303014621 | 0.008055791 |
| ENSG00000234449 | RP11-706O15.3 |  | 4203.754418 | 1718.63791 | 0.408834042 | -1.290412766 | 0.001449036 |
| ENSG00000236914 | RP11-1008C21.2 |  | 70.86374018 | 29.23195765 | 0.412509382 | -1.277501164 | 0.009639243 |
| ENSG00000081148 | IMPG2 | interphotoreceptor matrix proteoglycan 2 | 151.6962843 | 63.45216937 | 0.418284269 | -1.257444354 | 0.009433782 |
| ENSG00000160307 | S100B | S100 calcium binding protein B | 1956.826915 | 859.7887491 | 0.439379049 | -1.186462016 | 0.004848701 |
| ENSG00000244405 | ETV5 | ets variant 5 | 2248.181415 | 990.0855866 | 0.440393991 | -1.183133309 | 0.00514616 |
| ENSG00000228203 | RNF144A-AS1 | RNF144A antisense RNA 1 | 638.0390663 | 289.2393581 | 0.453325468 | -1.141380881 | 0.00907533 |
| ENSG00000266709 | RP11-214O1.2 |  | 180.3731223 | 83.15072052 | 0.460992854 | -1.117183706 | 0.009632473 |
| ENSG00000185742 | C11orf87 | chromosome 11 open reading frame 87 | 4265.305901 | 2021.020887 | 0.473827888 | -1.077564981 | 0.007979133 |
| ENSG00000171488 | LRRC8C | leucine rich repeat containing 8 family, member C | 772.8671972 | 368.3634476 | 0.476619332 | -1.069090624 | 0.008320898 |
| ENSG00000235436 | DPY19L2P4 | DPY19L2 pseudogene 4 | 121.548024 | 273.5756212 | 2.25076157 | 1.170413236 | 0.006060082 |
| ENSG00000246174 | KCTD21-AS1 | KCTD21 antisense RNA 1 | 95.2773559 | 214.7840998 | 2.25430374 | 1.172681914 | 0.007353828 |
| ENSG00000263146 | RP11-849I19.1 |  | 126.903035 | 300.5693381 | 2.368496058 | 1.243971271 | 0.003087398 |
| ENSG00000204305 | AGER | advanced glycosylation end product-specific receptor | 99.50402354 | 237.6391317 | 2.388236407 | 1.255945654 | 0.004134302 |
| ENSG00000264109 | MIR4712 | microRNA 4712 | 55.67480859 | 136.8467414 | 2.45796519 | 1.297464484 | 0.006746515 |
| ENSG00000223508 | RPL23AP53 | ribosomal protein L23a pseudogene 53 | 63.49906127 | 156.6369623 | 2.466760282 | 1.302617523 | 0.008014409 |
| ENSG00000237550 | RPL9P9 | ribosomal protein L9 pseudogene 9 | 2125.391381 | 5305.825879 | 2.496399452 | 1.3198488 | 0.005569897 |
| ENSG00000128242 | GAL3ST1 | galactose-3-O-sulfotransferase 1 | 16.51556349 | 43.27871017 | 2.620480384 | 1.38983131 | 0.008090516 |
| ENSG00000185985 | SLITRK2 | SLIT and NTRK-like family, member 2 | 1471.821744 | 4018.238583 | 2.730112257 | 1.448960273 | 0.001484301 |
| ENSG00000162623 | TYW3 | tRNA-yW synthesizing protein 3 homolog (S. cerevisiae) | 213.3532128 | 593.6760646 | 2.782597257 | 1.476432113 | 0.006447986 |
| ENSG00000237945 | LINC00649 | long intergenic non-protein coding RNA 649 | 120.6530652 | 356.5831337 | 2.955441979 | 1.563373898 | 0.002805001 |
| ENSG00000204805 | FAM27E4 | family with sequence similarity 27, member E4 | 25.18151957 | 74.60344896 | 2.962626967 | 1.566876984 | 0.004106621 |
| ENSG00000204791 | CTD-3065J16.6 |  | 17.05988657 | 52.91223383 | 3.10155836 | 1.632993272 | 0.002403442 |
| ENSG00000237198 |  |  | 33.90719086 | 108.5555194 | 3.2015486 | 1.678769911 | 0.001127604 |
| ENSG00000182368 |  |  | 9.441475591 | 31.28293919 | 3.313352759 | 1.728291809 | 0.007575185 |
| ENSG00000108439 | PNPO | pyridoxamine 5'-phosphate oxidase | 72.5876134 | 243.0551475 | 3.348438338 | 1.743488401 | 0.007865248 |
| ENSG00000261212 | RP3-507I15.2 |  | 8.652431131 | 29.59477685 | 3.420400164 | 1.774165121 | 0.0038107 |
| ENSG00000127903 | ZNF835 | zinc finger protein 835 | 98.06923514 | 340.3939369 | 3.470955355 | 1.795332809 | 0.006025298 |
| ENSG00000237917 | PARP4P1 | poly (ADP-ribose) polymerase family, member 4 pseudogene 1 | 4.379923292 | 16.57643192 | 3.784639778 | 1.920155991 | 0.009863625 |
| ENSG00000189398 | OR7E12P | olfactory receptor, family 7, subfamily E, member 12 pseudogene | 6.903531819 | 28.25875364 | 4.093376315 | 2.033291304 | 0.002560858 |
| ENSG00000259595 | RP11-516C1.1 |  | 4.182432881 | 17.15587186 | 4.101888148 | 2.036288152 | 0.004969869 |
| ENSG00000214688 | C10orf105 | chromosome 10 open reading frame 105 | 27.34722202 | 123.1382899 | 4.502771424 | 2.170813244 | 0.00012558 |
| ENSG00000232833 |  |  | 9.037107226 | 41.08796645 | 4.546583925 | 2.184782984 | 0.000384257 |
| ENSG00000223642 | AC008277.1 |  | 2.549568609 | 11.7437633 | 4.606176611 | 2.203569728 | 0.006927376 |
| ENSG00000227394 | AC007386.3 |  | 4.345549188 | 20.11964645 | 4.629943325 | 2.210994533 | 0.002930446 |
| ENSG00000125618 | PAX8 | paired box 8 | 82.65998072 | 392.7873409 | 4.75184409 | 2.248487502 | 0.006062569 |
| ENSG00000058866 | DGKG | diacylglycerol kinase, gamma 90kDa | 76.10909957 | 369.8420973 | 4.859367663 | 2.280768592 | 0.002459868 |
| ENSG00000215156 | RP11-1023L17.2 |  | 11.29992752 | 61.47570497 | 5.440362767 | 2.443702854 | 0.004562092 |
| ENSG00000231527 | RP11-374M1.2 |  | 4.975861207 | 27.83819153 | 5.594647916 | 2.484047342 | 0.000773135 |
| ENSG00000225353 |  |  | 8.296572947 | 47.95828702 | 5.780493624 | 2.531192697 | 2.92560672071043e-05 |
| ENSG00000221870 | TMEM257 | transmembrane protein 257 | 26.40759388 | 152.9742378 | 5.79281242 | 2.534263949 | 0.000109933 |
| ENSG00000160180 | TFF3 | trefoil factor 3 (intestinal) | 53.19090635 | 314.8659899 | 5.919545492 | 2.565486409 | 0.004750455 |
| ENSG00000139865 | TTC6 | tetratricopeptide repeat domain 6 | 29.33041822 | 180.7568699 | 6.16277847 | 2.623580932 | 0.001212996 |
| ENSG00000232818 | RPS2P32 | ribosomal protein S2 pseudogene 32 | 4.059817105 | 25.43101537 | 6.264079075 | 2.647102425 | 0.003529206 |
| ENSG00000186481 | ANKRD20A5P | ankyrin repeat domain 20 family, member A5, pseudogene | 4.154226824 | 28.85460781 | 6.94584312 | 2.796149826 | 0.00098959 |
| ENSG00000165409 | TSHR | thyroid stimulating hormone receptor | 33.02674385 | 229.413534 | 6.946295859 | 2.79624386 | 0.000213845 |
| ENSG00000204807 |  |  | 1.310768337 | 9.418655109 | 7.185598587 | 2.845108344 | 0.007012146 |
| ENSG00000232358 | RP5-955M13.4 |  | 0.916044102 | 7.978444207 | 8.709672591 | 3.122618487 | 0.007999914 |
| ENSG00000152093 | CFC1B | cripto, FRL-1, cryptic family 1B | 9.566072074 | 85.80836374 | 8.970072886 | 3.165119708 | 7.30207897893805e-05 |
| ENSG00000182489 | XKRX | XK, Kell blood group complex subunit-related, X-linked | 2.267155785 | 23.14755061 | 10.2099515 | 3.351904108 | 0.002666228 |
| ENSG00000181234 | TMEM132C | transmembrane protein 132C | 279.4478845 | 2876.372345 | 10.29305464 | 3.363599285 | 0.000403052 |
| ENSG00000226292 | RP11-7G12.1 |  | 0.884931836 | 9.224636215 | 10.42412063 | 3.38185378 | 0.003378492 |
| ENSG00000171517 | LPAR3 | lysophosphatidic acid receptor 3 | 8.784633979 | 95.15179397 | 10.83161737 | 3.437176776 | 0.002624963 |
| ENSG00000247516 | MIR4458HG | MIR4458 host gene | 15.16245411 | 173.0237163 | 11.41132663 | 3.512394617 | 4.44717246095538e-11 |
| ENSG00000207457 | RNU6-476P | RNA, U6 small nuclear 476, pseudogene | 0.458022051 | 6.87194063 | 15.00351482 | 3.90722861 | 0.004051511 |
| ENSG00000253678 | RP11-981G7.3 |  | 0.498621623 | 7.520278244 | 15.08213421 | 3.914768688 | 0.004844173 |
| ENSG00000240184 | PCDHGC3 | protocadherin gamma subfamily C, 3 | 56.32550255 | 892.2043157 | 15.84014834 | 3.985513941 | 2.07730403523971e-11 |
| ENSG00000169436 | COL22A1 | collagen, type XXII, alpha 1 | 34.57390112 | 609.1195691 | 17.61790106 | 4.138970151 | 0.007876202 |
| ENSG00000138161 | CUZD1 | CUB and zona pellucida-like domains 1 | 1.343870349 | 31.22806837 | 23.23741154 | 4.538377468 | 2.6703101033415e-05 |
| ENSG00000255366 | RP11-1134I14.8 |  | 3.938374017 | 141.9883115 | 36.05252089 | 5.172028235 | 0.001344434 |
| ENSG00000204556 | CTD-2514C3.1 |  | 1.063447272 | 39.74561313 | 37.37431481 | 5.223975225 | 6.20349902118999e-07 |
| ENSG00000254348 | RP11-1134I14.4 |  | 0.492296752 | 19.85600575 | 40.33340797 | 5.333903406 | 0.00074711 |
| ENSG00000258702 | RP11-433J8.1 |  | 0.458022051 | 21.77481055 | 47.54096556 | 5.5710993 | 0.000192779 |
| ENSG00000231081 | RP4-760C5.3 |  | 0.211873675 | 13.55135188 | 63.95958293 | 5.999088626 | 4.52750191636109e-06 |
| ENSG00000175868 | CALCB | calcitonin-related polypeptide beta | 0.635621025 | 44.51751355 | 70.03782414 | 6.130062359 | 0.002391262 |
| ENSG00000147724 | FAM135B | family with sequence similarity 135, member B | 5.355945952 | 1019.295681 | 190.3110468 | 7.572215497 | 2.51751161397466e-09 |
| ENSG00000174080 | CTSF | cathepsin F | 1.304443465 | 561.2440446 | 430.255553 | 8.749050002 | 0.001514888 |

**Supplementary Table S9.** Functional annotation enrichment analysis of differentially expressed genes in AN neurons. Two functional annotation database were used: Gene Ontology and Reactome.

| **GO ID** | **GO Term** | **# of Genes** | **% of Associated Genes** | ***P*-value** | **Adjusted *P*-value** | **Associated Genes** |
| --- | --- | --- | --- | --- | --- | --- |
| REACTOME:16963 | Tachykinin receptors bind tachykinins | 3 | 60.0 | 6.77323E-06 | 0.000738283 | TAC1, TACR1, TACR2 |
| GO:0032224 | positive regulation of synaptic transmission, cholinergic | 3 | 42.9 | 2.3398E-05 | 0.002503587 | TAC1, TACR1, TACR2 |
| GO:0007217 | tachykinin receptor signaling pathway | 3 | 37.5 | 3.71927E-05 | 0.003905237 | TAC1, TACR1, TACR2 |
| GO:0032222 | regulation of synaptic transmission, cholinergic | 4 | 33.3 | 2.79807E-06 | 0.000310586 | HTR6, TAC1, TACR1, TACR2 |
| GO:0072050 | S-shaped body morphogenesis | 3 | 30.0 | 7.86634E-05 | 0.00802367 | HES5, PAX8, WT1 |
| GO:2000047 | regulation of cell-cell adhesion mediated by cadherin | 3 | 27.3 | 0.000107458 | 0.010853241 | FOXA1, RGCC, SERPINF2 |
| GO:0044331 | cell-cell adhesion mediated by cadherin | 3 | 21.4 | 0.000232461 | 0.022083782 | FOXA1, RGCC, SERPINF2 |
| GO:0072216 | positive regulation of metanephros development | 3 | 21.4 | 0.000232461 | 0.022083782 | PAX8, PDGFB, WT1 |
| GO:0009071 | serine family amino acid catabolic process | 3 | 20.0 | 0.000288687 | 0.026559185 | AMT, SDSL, THNSL2 |
| GO:0048670 | regulation of collateral sprouting | 3 | 20.0 | 0.000288687 | 0.026559185 | LPAR3, OMG, SPP1 |
| GO:0051917 | regulation of fibrinolysis | 3 | 16.7 | 0.000507708 | 0.042139734 | KLKB1, SERPINF2, THBS1 |
| GO:0006855 | drug transmembrane transport | 3 | 15.8 | 0.000598989 | 0.046721154 | ATP8B1, SH3D19, SLC22A2 |
| GO:0031639 | plasminogen activation | 3 | 15.8 | 0.000598989 | 0.046721154 | KLKB1, SERPINF2, THBS1 |
| GO:0007271 | synaptic transmission, cholinergic | 4 | 13.3 | 0.000136779 | 0.013677899 | HTR6, TAC1, TACR1, TACR2 |
| GO:0051496 | positive regulation of stress fiber assembly | 5 | 11.6 | 3.78951E-05 | 0.003941089 | CTGF, RGCC, SERPINF2, TAC1, TACR1 |
| GO:0072207 | metanephric epithelium development | 4 | 11.4 | 0.000252473 | 0.023732496 | HES5, PAX8, PDGFB, WT1 |
| GO:0003338 | metanephros morphogenesis | 4 | 11.1 | 0.000282082 | 0.026233645 | FRAS1, HES5, PAX8, WT1 |
| GO:0032233 | positive regulation of actin filament bundle assembly | 5 | 10.6 | 5.8687E-05 | 0.006044762 | CTGF, RGCC, SERPINF2, TAC1, TACR1 |
| GO:0051492 | regulation of stress fiber assembly | 5 | 8.1 | 0.000222468 | 0.021356917 | CTGF, RGCC, SERPINF2, TAC1, TACR1 |
| GO:0010634 | positive regulation of epithelial cell migration | 8 | 8.0 | 3.00145E-06 | 0.000330159 | ANXA3, HBEGF, ITGB3, PDGFB, TAC1, TACR1, TEK, THBS1 |
| GO:0032355 | response to estradiol | 8 | 8.0 | 3.00145E-06 | 0.000330159 | ANXA1, CTGF, FOXA1, HTR6, MAPK15, PDGFB, SLC34A2, TACR1 |
| GO:0010595 | positive regulation of endothelial cell migration | 5 | 7.5 | 0.000320417 | 0.028837516 | ANXA3, ITGB3, PDGFB, TEK, THBS1 |
| GO:0043627 | response to estrogen | 13 | 7.4 | 5.46521E-09 | 6.17569E-07 | ANXA1, CTGF, FOXA1, GBA, HTR6, KRT19, MAPK15, PDGFB, RCAN1, SLC34A2, TACR1, TEK, TNFRSF11B |
| GO:0032231 | regulation of actin filament bundle assembly | 5 | 7.4 | 0.000343396 | 0.030218833 | CTGF, RGCC, SERPINF2, TAC1, TACR1 |
| GO:0042490 | mechanoreceptor differentiation | 5 | 7.4 | 0.000343396 | 0.030218833 | ATP8B1, BDNF, CECR2, HES5, TSHR |
| GO:1901343 | negative regulation of vasculature development | 5 | 6.7 | 0.000540973 | 0.0443598 | KLKB1, RGCC, TEK, THBS1, WT1 |
| GO:0043149 | stress fiber assembly | 5 | 6.6 | 0.000574972 | 0.045997772 | CTGF, RGCC, SERPINF2, TAC1, TACR1 |
| GO:0050806 | positive regulation of synaptic transmission | 6 | 5.9 | 0.000296634 | 0.026993671 | HTR6, PLK2, S100B, TAC1, TACR1, TACR2 |
| GO:0007613 | memory | 6 | 5.7 | 0.000346886 | 0.030179088 | NETO1, PLK2, RCAN1, S100B, TAC1, TACR1 |
| GO:0010632 | regulation of epithelial cell migration | 9 | 5.6 | 1.37317E-05 | 0.001483026 | ANXA3, HBEGF, ITGB3, PDGFB, RGCC, TAC1, TACR1, TEK, THBS1 |
| GO:0010594 | regulation of endothelial cell migration | 6 | 5.4 | 0.000467252 | 0.039249162 | ANXA3, ITGB3, PDGFB, RGCC, TEK, THBS1 |
| GO:0007612 | learning | 7 | 5.3 | 0.000168779 | 0.016371579 | BDNF, FOS, HTR6, NETO1, TAC1, TACR1, TACR2 |
| GO:0051017 | actin filament bundle assembly | 6 | 5.2 | 0.000564043 | 0.045687476 | CTGF, EPS8, RGCC, SERPINF2, TAC1, TACR1 |
| GO:0061572 | actin filament bundle organization | 6 | 5.2 | 0.000564043 | 0.045687476 | CTGF, EPS8, RGCC, SERPINF2, TAC1, TACR1 |
| GO:0045931 | positive regulation of mitotic cell cycle | 6 | 5.2 | 0.000590512 | 0.046650472 | ANXA1, FOXA1, NEUROG1, PDGFB, RGCC, TGFA |
| GO:0048545 | response to steroid hormone | 20 | 5.0 | 4.86096E-10 | 5.5415E-08 | ANXA1, CTGF, FOS, FOXA1, GBA, HTR6, KRT19, MAPK15, NR3C2, PDGFB, RCAN1, S100B, SCGB1A1, SLC34A2, SPP1, TACR1, TEK, THBS1, TMEM37, TNFRSF11B |
| GO:0031032 | actomyosin structure organization | 7 | 4.8 | 0.0003283 | 0.029218682 | CNN1, CTGF, KRT19, RGCC, SERPINF2, TAC1, TACR1 |
| GO:1903035 | negative regulation of response to wounding | 7 | 4.5 | 0.00045312 | 0.038515233 | GBA, KLKB1, PDGFB, SERPINF2, SPP1, TEK, THBS1 |
| GO:0007611 | learning or memory | 10 | 4.5 | 2.85809E-05 | 0.003029577 | BDNF, FOS, HTR6, NETO1, PLK2, RCAN1, S100B, TAC1, TACR1, TACR2 |
| GO:0097305 | response to alcohol | 14 | 4.4 | 8.55067E-07 | 9.57675E-05 | ANXA1, CTGF, EPS8, FOS, FOXA1, GBA, HTR6, MAPK15, PDGFB, RBP4, SLC34A2, SPP1, TACR1, THBS1 |
| GO:0010631 | epithelial cell migration | 9 | 4.1 | 0.000150536 | 0.014903047 | ANXA3, HBEGF, ITGB3, PDGFB, RGCC, TAC1, TACR1, TEK, THBS1 |
| GO:0048839 | inner ear development | 8 | 4.1 | 0.000355981 | 0.030614339 | ATP8B1, BDNF, CECR2, HES5, MYO3A, NEUROG1, PAX8, TSHR |
| GO:0090132 | epithelium migration | 9 | 4.1 | 0.00016674 | 0.016340488 | ANXA3, HBEGF, ITGB3, PDGFB, RGCC, TAC1, TACR1, TEK, THBS1 |
| GO:0001938 | positive regulation of endothelial cell proliferation | 3 | 4.0 | 0.029111988 | 0.029111988 | ITGB3, PDGFB, TEK |

*Term *P*-Value corrected with Bonferroni step down
